# CSNK2B Expression Aligns with a GDF15-Associated Immune Molecular State in Hepatocellular Carcinoma

**DOI:** 10.3390/biomedicines14091909

**Published:** 2026-08-26

**Authors:** Huiru Dai, Yajie Li, Junjie Zhang, Minling Liu, Tingwei Li, Yafei You, Jiancheng Wang, Shuo Fang

**Affiliations:** 1 Department of Oncology, The Seventh Affiliated Hospital of Sun Yat-sen University, Shenzhen 518107, China; daihuiru@sysush.com (H.D.); curtisneal250@gmail.com (Y.L.); zhangjj269@mail2.sysu.edu.cn (J.Z.); liuminling@sysush.com (M.L.); litingwei@sysush.com (T.L.); 2 Scientific Research Center, The Seventh Affiliated Hospital of Sun Yat-sen University, Shenzhen 518107, China

**Keywords:** hepatocellular carcinoma, CSNK2B, tumor immune microenvironment, GDF15

## Abstract

**Background:** Hepatocellular carcinoma (HCC) remains a major cause of cancer-related mortality, and responses to immune checkpoint blockade are often limited. Casein kinase 2 beta (CK2beta), encoded by CSNK2B, has established tumor-intrinsic functions, but its relationship with immune states in HCC remains unclear. **Methods:** We integrated TCGA-LIHC, six GEO cohort-platform combinations, GSE149614 single-cell RNA sequencing data, CIBERSORTx deconvolution, broad-compartment CellChat inference, and public proteomic resources. We evaluated CSNK2B expression, overall survival, a prespecified GDF15-associated Treg/checkpoint transcriptional module, tumor microenvironment features, compartment-level localization, and protein-level support. **Results:** CSNK2B was upregulated in TCGA-LIHC and all six external cohort-platform comparisons but was not a robust prognostic marker. The strongest immune association was with the GDF15-associated Treg/checkpoint module (Spearman rho = 0.371, q = 2.9 × 10^−12^); the direction was positive in all six GEO analyses and four passed BH-FDR. Deconvolution results were small and heterogeneous. CSNK2B and GDF15 were most prominent in hepatocyte-like compartments, while the candidate hepatocyte-to-T/NK GDF15–TGFBR2 pair was not reproduced in pooled data or in any of 10 patient-stratified CellChat runs. Public proteomic datasets supported tumor-side elevation of both proteins. **Conclusions:** CSNK2B expression aligns with a reproducible GDF15-associated transcriptional state in HCC, accompanied by selective stromal, endothelial, and immune-context changes. The evidence is associative and provides hypotheses for perturbation, spatial, and co-culture validation rather than a causal or treatment-prediction claim.

## 1. Introduction

Hepatocellular carcinoma (HCC) is a common malignancy with high mortality and poor clinical outcomes [1]. In China, almost 400,000 new cases are diagnosed annually [2]. Five-year survival was approximately 14.4% in 2019–2021 [3], and nearly half of patients are diagnosed at an advanced stage. Immunotherapy has shown antitumor activity in advanced HCC and has become an important first-line treatment option [4]. However, many patients have limited or transient responses. Defining the molecular features associated with immune states and treatment sensitivity therefore remains an important question in HCC research.

The tumor immune microenvironment (TIME) represents a major determinant of immune tolerance and therapeutic resistance in HCC. It is shaped by a complex network of cellular and molecular interactions, including impaired infiltration and activation of cytotoxic CD8^+^ T cells, accumulation of immunosuppressive populations such as myeloid-derived suppressor cells (MDSCs) and regulatory T cells (Tregs), and activation of immune checkpoint pathways including PD-1/PD-L1, CTLA-4, and TIGIT [5,6]. Collectively, these mechanisms constrain antitumor immune responses and contribute to resistance to immune checkpoint blockade. However, current understanding of immune escape mechanisms in HCC has largely focused on immune cell composition, immune checkpoint expression, and inflammatory cytokine networks. In contrast, how tumor-cell-intrinsic molecular programs actively shape the immune ecosystem remains incompletely understood.

CK2β, encoded by the CSNK2B gene, is a regulatory subunit of protein kinase CK2, consisting of 215 amino acids with a molecular weight of approximately 24.9 kDa. Within the CK2 holoenzyme complex, CK2β not only stabilizes the catalytic subunits and modulates kinase activity and substrate specificity but also functions as a scaffold protein that facilitates interactions among multiple substrates and regulatory factors [7]. Previous studies of CK2β have predominantly focused on its intrinsic oncogenic functions in tumor cells, including regulation of cell proliferation, apoptosis, DNA damage repair, and genomic stability [8]. However, the potential role of CK2β in shaping the tumor immune microenvironment remains largely unexplored.

Our preliminary analyses showed that CSNK2B was upregulated in HCC and associated with several immune-related features, whereas survival findings were inconsistent. Evidence from other systems also suggests that CK2β can participate in regulatory T-cell differentiation, B-cell function, and inflammatory programs [9,10]. These observations raise the possibility that CK2β is linked to immune regulatory networks, but its relevance to the HCC immune microenvironment remains uncertain.

We therefore integrated public transcriptomic, single-cell, deconvolution, and proteomic resources to test whether CSNK2B expression is associated with a reproducible immune-regulatory state in HCC. The analysis was designed to distinguish robust cross-cohort transcriptional findings from weaker deconvolution, survival, and immunotherapy-response signals.

## 2. Methods

### 2.1. Cohort Collection and Preprocessing

The discovery analysis used TCGA-LIHC data obtained from the GDC Data Portal [11], including 424 samples: 371 primary tumors, 50 solid-tissue normal samples, and three recurrent tumors. The prespecified gene panel comprised CSNK2B, GDF15, FOXP3, IL2RA, CTLA4, TIGIT, LAYN, TGFB1, CD8A, CD8B, PDCD1, CD274, CXCL12, and CCL22; all 14 genes were present. Clinical annotations were deduplicated at the patient level before integration with expression data.

External validation used six GEO cohort-platform combinations from NCBI GEO [12,13]: GSE14520-GPL3921, GSE14520-GPL571, GSE25097-GPL10687, GSE36376-GPL10558, GSE54236-GPL6480, and GSE76427-GPL10558. A cohort-platform combination was included when the expression matrix was accessible, tumor/reference labels were traceable, platform annotation was available, the prespecified panel could be mapped, and duplicate patient counting could be avoided. The two GSE14520 platforms were analyzed as platform-specific measurements of the same series, not as independent patient cohorts. GPL3921 annotations were matched before gene-level extraction. When multiple probes mapped to a gene, the available probe with the highest sample-wise variance was selected; all candidate probes, the selected probe, and its variance were recorded. For GSE76427, if the selected-probe 95th percentile exceeded 100, we used offset = max(0, 1 − min(x)) and transformed values as log2(x + offset); otherwise no log transformation was applied. This platform-specific rule handled high-intensity values with non-positive observations and did not constitute cross-platform harmonization.

### 2.2. Expression, Survival and Signature Analyses

CSNK2B expression was compared between TCGA-LIHC tumors and normal tissues using a two-sided Mann–Whitney U test. GEO tumor-reference comparisons used cohort-specific reference groups, including adjacent non-tumor, cirrhotic, or healthy tissues when available. Overall survival was the only usable local TCGA endpoint. The continuous z-scored CSNK2B Cox model was the primary survival analysis; a median split was used for visualization. Median, upper-tertile, upper-quartile, and upper-versus-lower-quartile comparisons were prespecified exploratory sensitivity analyses, no data-driven optimal cutoff was selected, and BH correction was applied across that four-test family.

Bulk immune and tumor-microenvironment (TME) modules were scored from standardized expression-based signatures. The prespecified GDF15-associated Treg/checkpoint transcriptional module contained CSNK2B, GDF15, FOXP3, IL2RA, CTLA4, TIGIT, LAYN, and TGFB1 and was scored as the mean of gene-wise standardized expression. It is a bulk transcriptomic module, not a direct measurement of Treg abundance or a single-cell Treg signature. The focused immune layer also included Treg-marker and checkpoint modules together with selected Reactome, Hallmark, GO, and MSigDB signatures related to PD-1/CTLA4 co-inhibition, TGF-beta signaling, EMT, hypoxia, IL6-STAT3, NF-kappaB, and PI3K-AKT-mTOR biology [14,15]. TME modules included endothelial, fibroblast/CAF, extracellular-matrix, angiogenesis, broad stromal, broad suppressive, broad effector, and escape-balance composites. Continuous associations used Spearman correlation. High-low comparisons used Mann–Whitney tests and rank-biserial effect sizes.

### 2.3. CIBERSORTx, Public Single-Cell Data and Protein-Level Validation

Online CIBERSORTx Digital Cytometry was used to estimate LM22 immune-cell fractions from TCGA-LIHC primary tumors and GEO tumor-only mixtures [16,17]. Quantile normalization was disabled for the TCGA RNA-seq mixture because quantile normalization is not recommended for RNA-seq-derived mixtures, whereas it was enabled for the GEO microarray mixtures to reduce platform-specific distributional effects for which the LM22 workflow was originally developed. These platform-adapted settings were specified before downstream comparisons and were used as a technical sensitivity analysis, not to force RNA-seq and microarray data onto a common scale. Returned files were aligned with continuous CSNK2B expression and the descriptive median split. Because LM22 fractions are bounded, often non-normal, and may contain many zeros, analyses used rank-based statistics. These values were treated as computational estimates rather than direct measurements of immune infiltration and are presented as supplementary sensitivity analyses.

Public GSE149614 count and normalized-expression matrices [18] were summarized using the author-provided cell annotations. The downloaded files did not contain validated UMAP coordinates, malignant-cell assignments, or spatial information. The single-cell objective was therefore compartment-level localization rather than de novo integration, embedding, trajectory, or lineage inference. To avoid batch-dependent re-embedding, no de novo Seurat integration/embedding [19] or Monocle trajectory/pseudotime inference [20] was performed. CellChat v2.2.0.9001 [21] was used only as an exploratory broad-compartment check in pooled tumor-site cells and 10 patient-stratified runs, using six author-defined compartments and a combined T/NK receiver label. The analysis used a deterministic cap of 200 cells per patient-cell-type stratum, CellChatDB.human, a triMean estimator, and a *p* < 0.05 communication gate. The focal candidate pair was hepatocyte-to-T/NK GDF15–TGFBR2. Because the analysis is expression-based, lacks spatial coordinates, and does not contain a Treg-specific receiver label, it cannot establish physical proximity, direct crosstalk, or Treg-specific communication. Protein-level validation used UALCAN, PDC/CPTAC, and Human Protein Atlas resources [22,23,24]. These resources supported tumor-side protein abundance, not phosphosite-level mechanisms.

### 2.4. Immunotherapy-Related Exploratory Analyses

GSE202069 [25] and GSE285963 [26] were treated as exploratory public HCC immunotherapy cohorts and interpreted alongside recent studies of atezolizumab–bevacizumab response patterns and genomic biomarkers [27,28]. Sample-level RECIST labels for GSE202069 yielded 17 anti-PD-1-treated cases. The available GSE285963 supplements reported aggregate BOR, ORR, and PFS but did not provide a CU24-to-GSM response map, so no sample-level expression-response test was performed for that cohort. TIDEpy and TCIA/IPS were used only as exploratory context because neither provides validated HCC-specific CSNK2B response prediction [29,30]. These analyses are confined to Supplementary Figure S1 and are not used to make predictive or therapeutic claims [31].

### 2.5. Statistical, Reporting and Reproducibility Principles

All tests were two-sided. We report nominal *p* values together with BH-FDR q values, using q < 0.05 as the corrected significance threshold. BH correction was applied within prespecified families defined by endpoint and analytic scope: four survival-cutoff sensitivity tests; TCGA signature correlations; TCGA signature high-low tests; six GEO GDF15-associated module replication tests; TCGA TME correlations; TCGA TME high-low tests; 22 LM22 cell types within each continuous or high-low analysis; 18 selected GEO cohort-by-cell LM22 tests; focused paired-proteomic comparisons; and 20 GSE202069 response-feature tests. CellChat communication gates were descriptive pooled and patient-stratified checks rather than a Treg-specific inferential family. Unrelated evidence layers were not pooled into one correction family. Nominal findings that did not pass BH-FDR were described as nonsignificant after correction. Continuous associations are reported as Spearman rho, high–low contrasts as rank-biserial r, survival models as hazard ratios with 95% confidence intervals, and exploratory response effects as AUCs. The value rho^2^ was used only as the arithmetic square of the rank-correlation coefficient to describe magnitude; no linear-regression coefficient of determination (R^2^) or variance-explained estimate was fitted. Core data processing and statistical analyses were run in Python 3.12.6 with pandas 2.3.3, NumPy 2.3.3, SciPy 1.15.3, and statsmodels 0.14.5. CellChat v2.2.0.9001 was run in R with CellChatDB.human. Submission figures were generated from archived ggplot2-based scripts in a Dockerized R environment configured for R 4.2 or later. Raw public data were retained unchanged, and derived outputs were generated by reproducible local scripts.

## 3. Results

### 3.1. CSNK2B Is Consistently Upregulated in Hepatocellular Carcinoma but Shows Limited Evidence as a Conventional Prognostic Determinant

**Figure 1:**
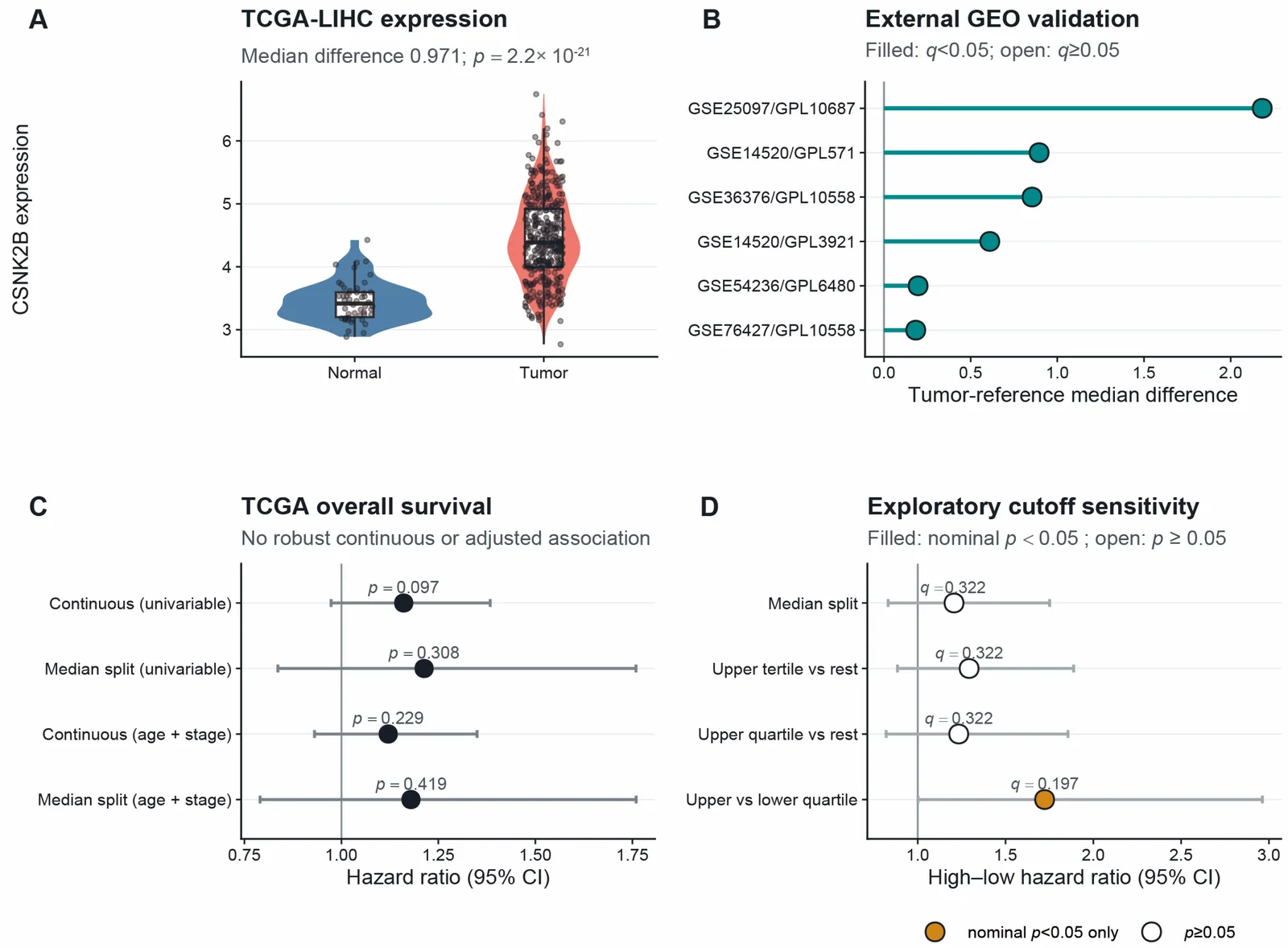
CSNK2B expression and overall-survival boundaries in HCC. (**A**) TCGA-LIHC primary-tumor and solid-tissue-normal expression. (**B**) Tumor-reference median differences in six GEO cohort-platform comparisons; all effects were tumor-high. (**C**) Unadjusted and adjusted continuous and median-split Cox models, shown as hazard ratios with 95% confidence intervals. (**D**) Four exploratory cutoff comparisons. BH correction was applied across the four cutoffs; the nominal upper-versus-lower-quartile result did not retain significance (*p* = 0.049, *q* = 0.197), and no cutoff passed *q* < 0.05. Points in (**A**) represent individual samples; filled and open circles in (**B**) indicate BH-FDR *q* < 0.05 and *q* ≥ 0.05, respectively; in (**D**), fill denotes nominal *p* < 0.05 versus *p* ≥ 0.05.

CSNK2B expression was higher in TCGA-LIHC primary tumors than in solid-tissue normal samples (median 4.386 versus 3.415; median difference 0.971; Mann–Whitney *p* = 2.2 × 10^−21^; Figure 1A). The tumor-high direction was reproduced in all six GEO cohort-platform comparisons (Figure 1B). Survival results were weaker (Figure 1C). The local TCGA analysis included 346 patients and 112 events. Continuous CSNK2B was not associated with overall survival after age and stage adjustment (HR = 1.121, *p* = 0.229), and the median split was also nonsignificant. The upper-versus-lower-quartile comparison was nominally significant (*p* = 0.049) but failed BH correction across the four exploratory cutoffs (*q* = 0.197); no cutoff retained *q* < 0.05 (Figure 1D).

### 3.2. CSNK2B Aligns with a GDF15-Associated Transcriptional Program in HCC

**Figure 2:**
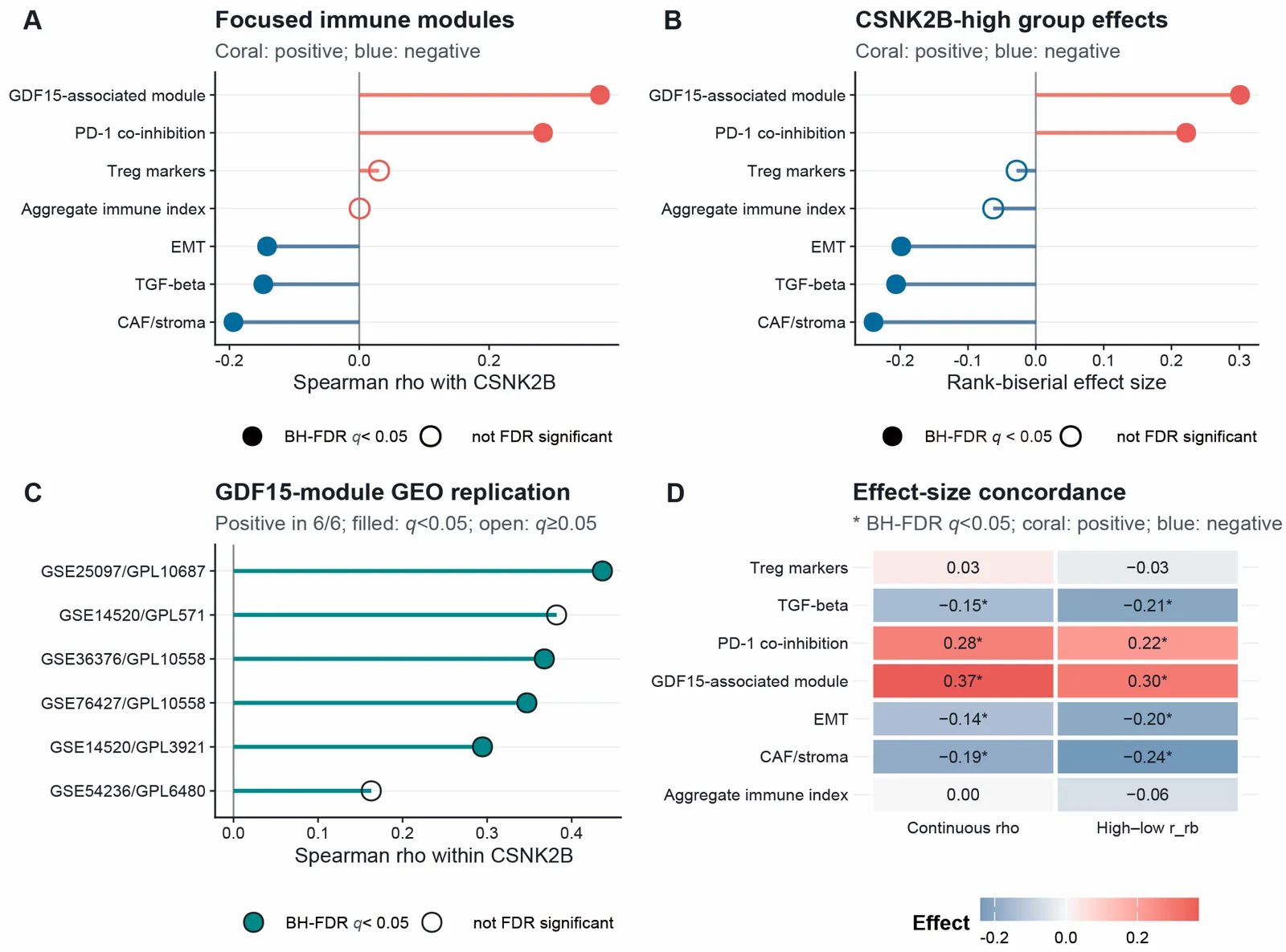
Replicated GDF15-associated Treg/checkpoint transcriptional module. (**A**) Spearman associations between CSNK2B and prespecified bulk signatures in TCGA-LIHC. BH correction was applied within the prespecified TCGA signature-correlation family. (**B**) Rank-biserial effects for CSNK2B high-low signature comparisons. BH correction was applied across the prespecified CSNK2B-high versus CSNK2B-low signature-comparison family. (**C**) Module replication across six GEO cohort-platform combinations; the direction was positive in 6/6 and 4/6 tests passed BH-FDR. (**D**) Concordance of continuous and high–low effect estimates. The module contains CSNK2B, GDF15, FOXP3, IL2RA, CTLA4, TIGIT, LAYN, and TGFB1; its reduced-module sensitivity analysis is reported in the text. In (**A**) and (**B**), coral and blue indicate positive and negative effects, respectively; filled and open circles indicate BH-FDR *q* < 0.05 and *q* ≥ 0.05, respectively.

In TCGA-LIHC primary tumors, the clearest immune association was the GDF15-associated Treg/checkpoint transcriptional module (Spearman rho = 0.371, *q* = 2.9 × 10^−12^; Figure 2A). This was a moderate rank association. Its arithmetic square (rho^2^ approximately 0.138) is reported only as a descriptive transformation of rho; it is not a linear-model R^2^ and does not represent an estimate of explained variance. CSNK2B-high tumors had higher module scores, whereas the aggregate immune index was not associated with CSNK2B and several stromal or matrix modules moved in the opposite direction (Figure 2B). The direction was positive in all six GEO cohort-platform combinations; four passed BH-FDR (Figure 2C). GSE14520-GPL571 (*n* = 22, *q* = 0.2352) and GSE54236-GPL6480 (*n* = 81, *q* = 0.3723) remained positive but did not pass the replication-family threshold, consistent with limited power and platform, reference-tissue, or patient-composition differences. As a sensitivity analysis, removing CSNK2B from the module reduced its association with CSNK2B to rho = 0.122 (*q* = 0.0545), and the high-low comparison was nonsignificant (*q* = 0.543), whereas GDF15 remained associated with the reduced module (rho = 0.336, *q* = 1.31 × 10^−9^). These results show that the signal is not mechanically driven by the inclusion of CSNK2B, but they do not establish that CSNK2B regulates GDF15.

### 3.3. CSNK2B-Associated Tumors Show Selective Bulk TME Remodeling Rather than Global Immunosuppression

**Figure 3:**
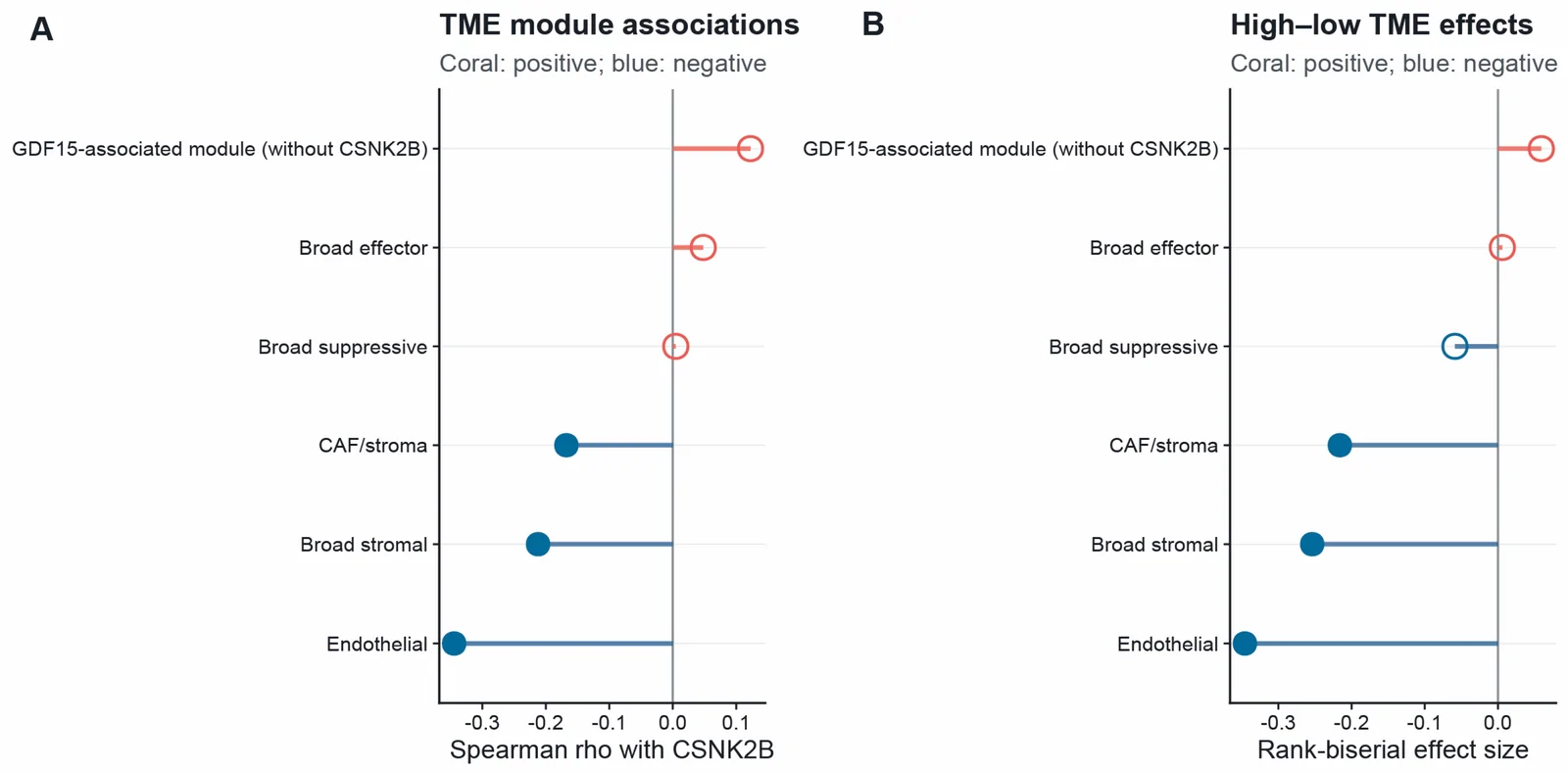
Selective bulk TME remodeling in CSNK2B-associated HCC. (**A**) Continuous Spearman associations between CSNK2B and prespecified bulk TME modules. (**B**) Rank-biserial effects for CSNK2B high–low module comparisons. The GDF15-associated module shown in panel (**A**) excludes CSNK2B. Detailed CIBERSORTx LM22 sensitivity analyses are provided in Supplementary Figure S3 and are not direct measurements of immune-cell infiltration. Coral and blue indicate positive and negative effects, respectively; filled and open circles indicate BH-FDR *q* < 0.05 and *q* ≥ 0.05, respectively.

TME signature analysis did not support a generalized immunosuppressive state. CSNK2B was associated with lower endothelial, broad stromal, and CAF/stromal scores in continuous and high-low analyses (Figure 3A,B). These associations describe a selective bulk transcriptional context and do not demonstrate reduced vascular density, matrix depletion, or treatment sensitivity. CIBERSORTx LM22 was retained as a secondary sensitivity layer: TCGA continuous estimates were small, the Treg median-split contrast was nonsignificant (rank-biserial r = 0.085, *q* = 0.315), and none of 18 selected GEO cohort-cell tests passed global BH-FDR. The full LM22 results are shown in Supplementary Figure S3 and are not interpreted as measured immune-cell infiltration.

### 3.4. Single-Cell Localization, Broad-Compartment Communication Checks and Proteomic Support

**Figure 4:**
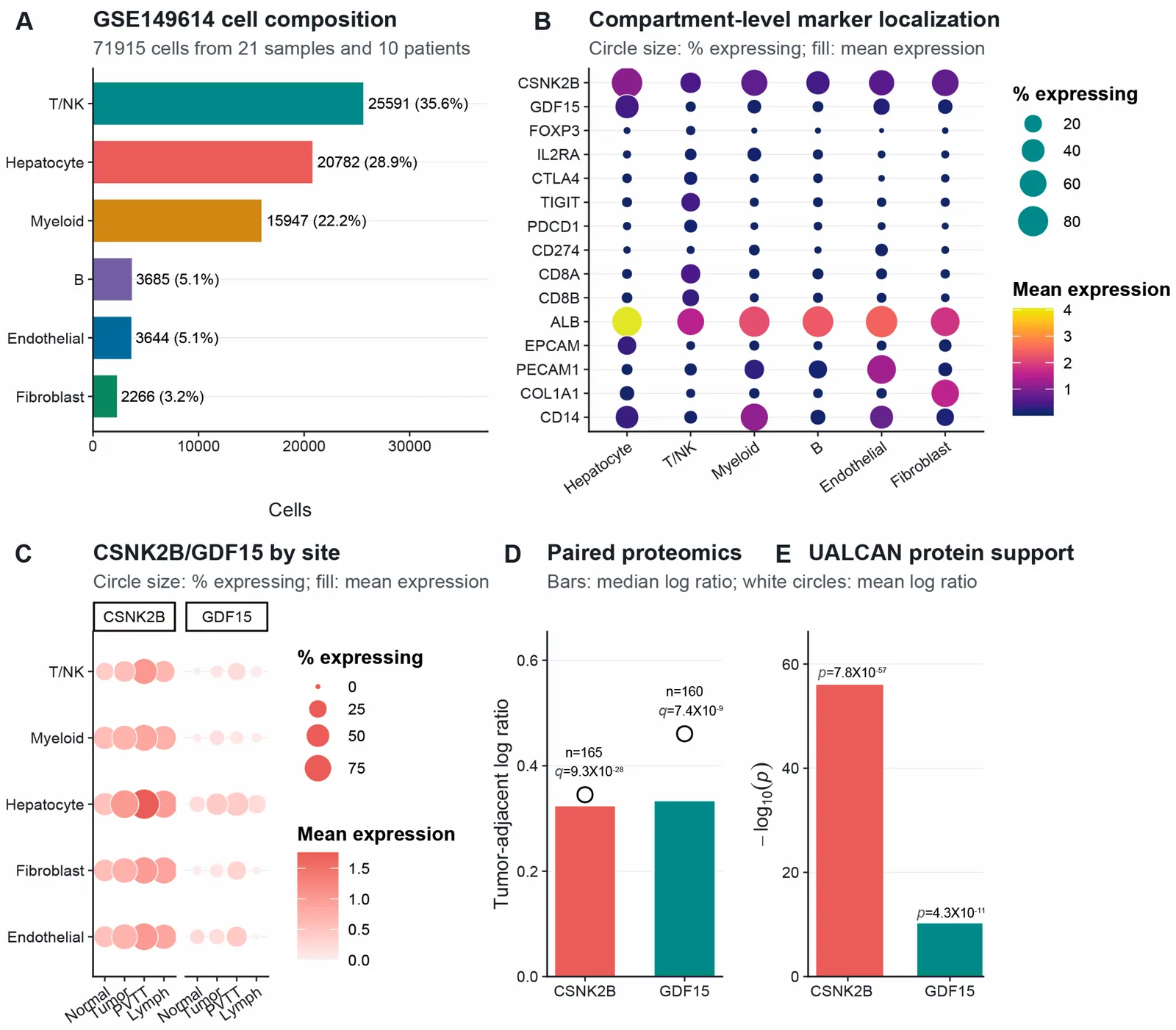
Compartment-level single-cell localization and protein support. (**A**) Author-annotated cell composition in GSE149614. (**B**) Target-gene expression across six annotated cell types. (**C**) Site-aware CSNK2B and GDF15 expression summaries. These panels show compartment-level localization only and do not assign malignant status. (**D**) Paired PDC/CPTAC tumor-adjacent protein differences for CSNK2B (*n* = 165) and GDF15 (*n* = 160). (**E**) UALCAN protein-level support. Broad-compartment CellChat results are provided in Supplementary Figure S2. In the dot plots, circle size denotes the percentage of cells expressing the gene and fill color denotes mean normalized expression; in (**D**), bars show median tumor-adjacent log ratios and white circles show means.

GSE149614 comprised 71,915 cells from 21 samples, 10 patients, and six author-annotated cell types (Figure 4A). CSNK2B and GDF15 were most prominent in hepatocyte-like compartments, which cannot be assumed to be malignant cells, with lower signals in other annotated compartments. T-cell markers and checkpoint genes were concentrated mainly in T/NK cells, while CD274 was higher in endothelial and myeloid compartments (Figure 4B,C). This is compartment-level localization only. The candidate hepatocyte-to-T/NK GDF15–TGFBR2 pair was not detected in the pooled CellChat run and was not reproduced in any of the 10 patient-stratified analyses. This is a sensitivity boundary under the current labels and data structure, not evidence that communication is absent. Because T/NK is not a Treg-specific population and the data lack spatial coordinates, the analysis cannot establish physical proximity, signaling direction, or direct tumor-Treg communication. Detailed CellChat results are provided in Supplementary Figure S2. Focused BH-FDR correction retained paired PDC/CPTAC tumor-side increases for CSNK2B (*n* = 165 pairs) and GDF15 (*n* = 160 pairs), and UALCAN provided concordant protein-level support (Figure 4D,E).

### 3.5. Evidence Matrix and Cross-Cohort Replication Define the Association Boundary

**Figure 5:**
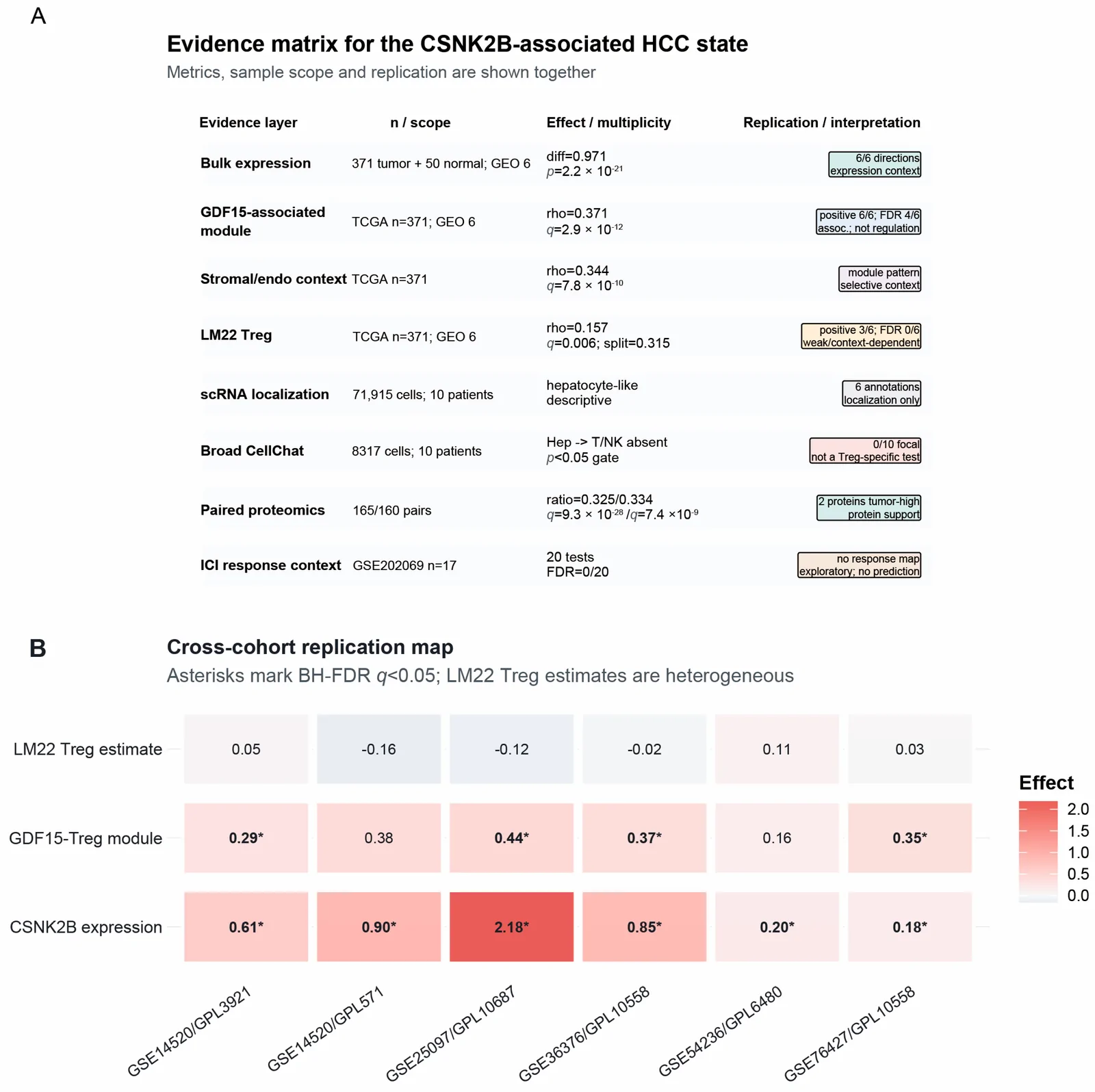
Evidence matrix and cross-cohort replication for the CSNK2B-associated HCC state. (**A**) Evidence layers are shown with sample scope, effect, *p* or *q* values, replication, and the appropriate interpretive boundary. (**B**) Cohort-level effects for CSNK2B expression, the GDF15-associated module, and heterogeneous LM22 Treg estimates. These panels summarize co-expression and functional context; they do not establish a regulatory network, protein interaction, causal direction, or direct cell–cell communication.

The evidence matrix summarized CSNK2B-associated transcriptional and TME patterns without treating them as a regulatory network (Figure 5A). It reports sample scope, effect estimates, *p* or *q* values, cross-cohort replication, and an interpretive boundary. The strongest retained associations included tumor-normal expression, the GDF15-associated Treg/checkpoint module, and negative endothelial, stromal, and CAF-related associations; effect scales differ across analysis types. The replication map contrasts stable tumor-expression and module directions with heterogeneous LM22 estimates (Figure 5B). These entries are co-expression and functional-context summaries; they do not establish protein interaction, molecular regulation, or causal direction.

### 3.6. Association Model and Prospective Validation Roadmap

**Figure 6:**
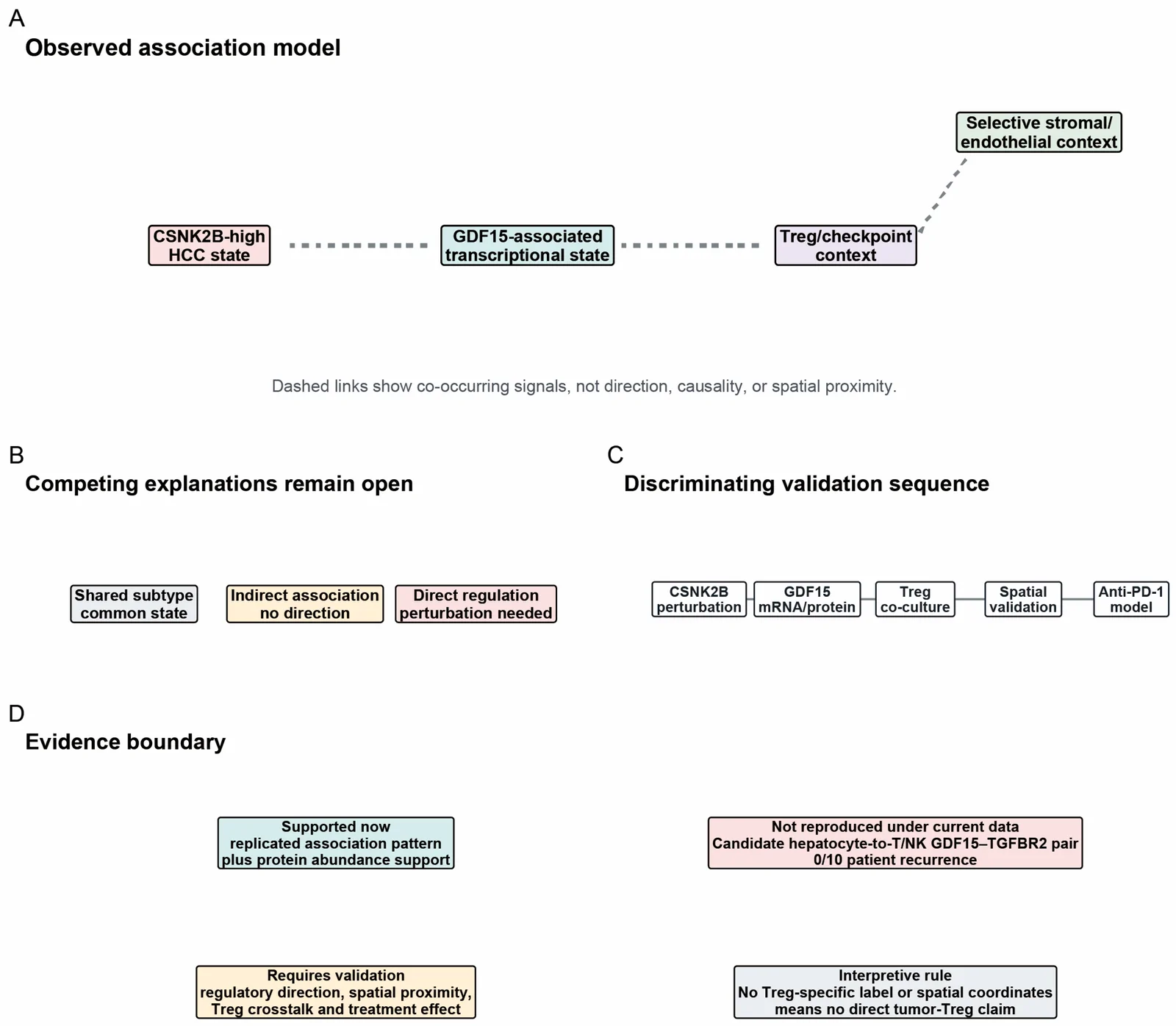
Co-expression model, competing explanations, and validation roadmap. (**A**) Non-directional dashed connectors depict co-occurring signals only; they do not represent causality, spatial proximity, or cell–cell communication. (**B**) Alternative explanations compatible with current public data. (**C**) Discriminating validation sequence. (**D**) Evidence supported now, evidence requiring validation, and the broad-compartment CellChat boundary.

The integrated summary treats the CSNK2B-high, GDF15-associated Treg/checkpoint pattern as a testable co-expression model rather than an established mechanism (Figure 6A). It distinguishes shared HCC subtype and indirect co-expression explanations from the direct-regulation hypothesis that requires perturbation evidence. SMAD-, STAT-, and NF-kappaB-related processes are literature-informed candidates for future testing, not pathways demonstrated by the current analyses. The prospective sequence begins with CSNK2B perturbation and GDF15 mRNA or protein measurement, followed by Treg co-culture and spatial tissue analysis, before any anti-PD-1 combination experiment. Each hypothesis can be rejected by experiment.

## 4. Discussion

### 4.1. CSNK2B Marks an Immune-Contextual Molecular State Rather than a Conventional Prognostic Factor

Prior studies have linked CSNK2B to tumor-cell proliferation, apoptosis, DNA repair, and genomic stability [32,33], with emphasis on tumor-intrinsic biology rather than the immune microenvironment. Its HCC immune context is therefore less defined. Our analyses place CSNK2B within a reproducible immune-related molecular state while avoiding assumptions about its causal role.

CSNK2B was consistently upregulated across TCGA-LIHC and six GEO cohort-platform comparisons, but survival analyses did not support independent prognostic value. Continuous and median-split models were nonsignificant, and the nominal quartile result failed correction. The present data therefore support CSNK2B as a molecular-context marker rather than a conventional survival biomarker. Studies in other tumor settings likewise distinguish molecular-state classification from validated individual-level prognosis [34,35].

### 4.2. CSNK2B Aligns with a GDF15-Associated Co-Expression Pattern

GDF15, a member of the transforming growth factor-beta superfamily, has been implicated in tumor-associated immune regulation, immune-cell recruitment, T-cell function, and checkpoint-blockade response [36,37,38].

The strongest observation was the cross-cohort alignment of CSNK2B with a GDF15-associated Treg/checkpoint state. The moderate rho = 0.371 association places CSNK2B within a broader co-expression pattern, while shared HCC subtype, stress, or inflammatory programs remain plausible alternatives. STRING [39] and related resources can organize functional context, but their heterogeneous evidence cannot establish regulation, directionality, disease-specific activity, or causality. SMAD-, STAT-, and NF-kappaB-related processes therefore remain candidates for testing rather than demonstrated mechanisms.

The data therefore support a testable co-expression hypothesis, not direct CK2beta control of GDF15, GDF15-mediated TME remodeling, or tumor-Treg crosstalk. Perturbation, co-culture, imaging, and spatial assays are needed to distinguish regulation from shared-subtype or broader transcriptional explanations.

### 4.3. CSNK2B-Associated Tumors Show Selective TME Features Rather than Global Immunosuppression

The TME cannot be reduced to one activation-suppression axis [40,41]. CSNK2B-high tumors combined a positive GDF15-associated module with lower CAF, stromal, and endothelial scores, whereas LM22 estimates were smaller and heterogeneous. This pattern describes a GDF15-enriched, relatively low-stromal transcriptional context, not generalized immunosuppression, treatment sensitivity, or a validated target.

Single-cell summaries localized CSNK2B and GDF15 mainly to hepatocyte-like compartments and Treg/checkpoint markers to immune compartments. The candidate GDF15–TGFBR2 pair was not reproduced in 10 patient-stratified CellChat analyses, but broad T/NK labels and absent spatial coordinates preclude conclusions about proximity, direction, or tumor-Treg communication.

A main strength is the agreement between TCGA and GEO cohorts for CSNK2B upregulation and the GDF15-associated module, supported by an independent protein layer. The analysis also distinguishes these findings from weaker survival, LM22, immunotherapy, and CellChat evidence. Reporting negative and heterogeneous results alongside the replicated findings helps define the evidential boundary rather than forcing a single mechanistic narrative. This preserves biological context while keeping interpretation proportional to the evidence.

### 4.4. Limitations

This retrospective public-data study is limited by residual batch effects, incomplete covariates, platform-specific probes, and lack of experimental validation. LM22 fractions are inferred and often zero-inflated; GEO estimates were heterogeneous. The single-cell data lacked malignant-cell labels and spatial coordinates, and 0/10 CellChat recurrence does not prove absence. The immunotherapy cohort was small (*n* = 17), with no BH-FDR-positive feature. STRING provides functional-association context only; its evidence channels do not quantify manuscript reliability or replace disease-specific perturbation and protein-interaction experiments.

### 4.5. Testable Predictions

Three experiments would discriminate among the current explanations: test whether CSNK2B perturbation alters GDF15 expression or secretion; determine whether GDF15 rescue or blockade changes Treg-related co-culture effects; and use spatial tissue assays to establish whether relevant tumor and immune compartments can interact. Negative results would narrow or reject the working model before combination-treatment studies.

## 5. Conclusions

CSNK2B is consistently upregulated in hepatocellular carcinoma and aligns with a replicated GDF15-associated Treg/checkpoint transcriptional state and selective bulk TME changes. Survival, LM22 deconvolution, immunotherapy-response, and broad-compartment communication analyses are weaker or exploratory and do not support prognostic, direct-interaction, or treatment-prediction claims. The study defines a reproducible association pattern and testable experiments, not a causal regulatory network.

The cross-cohort consistency supports a testable co-expression framework, while perturbation, spatial, and tumor-immune co-culture studies are required to determine whether the CSNK2B/GDF15 pattern reflects a functional relationship or a broader HCC transcriptional program.

## Data Availability

All datasets analyzed in this study were obtained from public resources. TCGA-LIHC transcriptomic and clinical data were obtained from the GDC Data Portal [11]. GEO datasets were obtained from NCBI GEO [13], including GSE14520, GSE25097, GSE36376, GSE54236, and GSE76427; GSE149614 [18], GSE202069 [25], and GSE285963 [26] were used for single-cell or exploratory immunotherapy analyses. CIBERSORTx Digital Cytometry was accessed through its web resource [42]. Gene sets and pathway annotations were obtained from MSigDB/GSEA [43] and Reactome [44]. Exploratory immune-response resources included TIDE [44] and TCIA/IPS [45]. Protein and association resources included UALCAN [46], Proteomic Data Commons [47], Human Protein Atlas [48], STRING [49], and LinkedOmics [50]. CellChat was run locally on processed GSE149614 matrices using CellChatDB.human [18]. Derived matrices, CellChat outputs, result tables, source-data indexes, and analysis scripts are included in Supplementary File S1.

## Supplementary Materials

The following supporting information can be downloaded at https://www.mdpi.com/article/10.3390/biomedicines14091909/s1. Supplementary Figure S1. Exploratory immunotherapy-response context. Response-feature AUCs in 17 GSE202069 anti-PD-1-treated cases; none of the 20 selected feature tests passed BH-FDR. Local TIDEpy outputs are not validated for HCC treatment prediction. Supplementary Figure S2. Broad-compartment CellChat evidence boundary. The combined T/NK label is not Treg-specific, and expression-based inference without spatial coordinates cannot establish physical contact or direct crosstalk. Supplementary Figure S3. CIBERSORTx LM22 sensitivity analyses. Continuous estimated-fraction associations were small, the Treg median-split contrast was nonsignificant, and no selected GEO cohort-cell test passed global BH-FDR; LM22 values are computational estimates rather than measured infiltration. Supplementary File S1. Source data and reproducibility package containing derived result tables, source-data indexes, and scripts used for the public-data analyses.

## Abbreviations

The following abbreviations are used in this manuscript:

CK2B	casein kinase II subunit beta
HCC	hepatocellular carcinoma
TCGA	The Cancer Genome Atlas
TME	tumor-microenvironment
