# Supplementary material for: CSNK2B Expression Aligns with a GDF15-Associated Immune Molecular State in Hepatocellular Carcinoma
Source: Biomedicines. 2026 Aug 26;14(9):1909. doi: 10.3390/biomedicines14091909 (PMC13604900; doi:10.3390/biomedicines14091909)
Supplement: Supplementary file 1 [file biomedicines-14-01909-s001.zip › Supplemental file S1/docker_cellchat/CellChat-main-http1.tar.gz!/CellChat-main/tutorial/CellChat-vignette.html]

Inference and analysis of cell-cell communication using CellChat


# Inference and analysis of cell-cell communication using CellChat

#### Suoqin Jin

#### 14 February, 2024

- Load the required
  libraries
- Part
  I: Data input & processing and initialization of CellChat object
  - Prepare
    required input data for CellChat analysis
    - (A) Starting from a count
      data matrix
    - (B) Starting from a Seurat
      object
    - (C) Starting from
      a SingleCellExperiment object
    - (D) Starting from an Anndata
      object
  - Create a CellChat object
    - (A)
      Starting from the digital gene expression matrix and cell label
      information
    - (B) Starting from a Seurat
      object
    - (C) Starting
      from a SingleCellExperiment object
    - (D) Starting from an
      AnnData object
  - Set the
    ligand-receptor interaction database
  - Preprocessing
    the expression data for cell-cell communication analysis
- Part II:
  Inference of cell-cell communication network
  - Compute
    the communication probability and infer cellular communication
    network
  - Extract
    the inferred cellular communication network as a data frame
  - Infer
    the cell-cell communication at a signaling pathway level
  - Calculate
    the aggregated cell-cell communication network
- Part
  III: Visualization of cell-cell communication network
  - Visualize
    each signaling pathway using Hierarchy plot, Circle plot or Chord
    diagram
    - Compute
      the contribution of each ligand-receptor pair to the overall signaling
      pathway and visualize cell-cell communication mediated by a single
      ligand-receptor pair
    - Automatically
      save the plots of the all inferred network for quick
      exploration
  - Visualize
    cell-cell communication mediated by multiple ligand-receptors or
    signaling pathways
    - (A) Bubble
      plot
    - (B) Chord
      diagram
  - Plot
    the signaling gene expression distribution using violin/dot
    plot
- Part
  IV: Systems analysis of cell-cell communication network
  - Identify
    signaling roles (e.g., dominant senders, receivers) of cell groups as
    well as the major contributing signaling
    - (A)
      Compute and visualize the network centrality scores
    - (B)
      Visualize dominant senders (sources) and receivers (targets) in a 2D
      space
    - (C)
      Identify signals contributing the most to outgoing or incoming signaling
      of certain cell groups
  - Identify
    global communication patterns to explore how multiple cell types and
    signaling pathways coordinate together
    - (A)
      Identify and visualize outgoing communication pattern of secreting
      cells
    - (B)
      Identify and visualize incoming communication pattern of target
      cells
  - Manifold
    and classification learning analysis of signaling networks
    - Identify
      signaling groups based on their functional similarity
    - Identify
      signaling groups based on structure similarity
- Part V: Save the CellChat
  object
- Part
  VI: Explore the cell-cell communication through the Interactive CellChat
  Explorer

This vignette outlines the steps of inference, analysis and
visualization of cell-cell communication network for **a single
dataset using CellChat**. We showcase CellChat’s diverse
functionalities by applying it to a scRNA-seq data on cells from
lesional (LS, diseased) human skin from patients.

CellChat requires gene expression data of cells as the user input and
models the probability of cell-cell communication by integrating gene
expression with prior knowledge of the interactions between signaling
ligands, receptors and their cofactors.

Upon infering the intercellular communication network, CellChat
provides functionality for further data exploration, analysis, and
visualization.

## Load the required libraries

```
library(CellChat)
library(patchwork)
options(stringsAsFactors = FALSE)
# reticulate::use_python("/Users/suoqinjin/anaconda3/bin/python", required=T)
```

# Part I: Data input & processing and initialization of CellChat object

CellChat requires two user inputs: one is the gene expression data of
cells, and the other is the user assigned cell labels.

## Prepare required input data for CellChat analysis

*For the gene expression data matrix*, genes should be in rows
with rownames and cells in columns with colnames. Normalized data (e.g.,
library-size normalization and then log-transformed with a pseudocount
of 1) is required as input for CellChat analysis. If user provides count
data, we provide a `normalizeData` function to account for
library size and then do log-transformed.

*For the cell group information*, a dataframe with rownames is
required as input for CellChat.

In addition to taking a count data matrix as an input, we also
provide instructions for how to prepare CellChat input files from other
existing single-cell analysis toolkits, including Seurat,
SingleCellExperiment and Scanpy. Please start to prepare the input data
by following option A when the normalized count data and meta data are
available, option B when the Seurat object is available, option C when
the SingleCellExperiment object is available, and option D when the
Anndata object is available. See details in the tutorial
on Interface\_with\_other\_single-cell\_analysis\_toolkits.

### (A) Starting from a count data matrix

```
ptm = Sys.time()
# Here we load a scRNA-seq data matrix and its associated cell meta data
# This is a combined data from two biological conditions: normal and diseases
load("/Users/suoqinjin/Library/CloudStorage/OneDrive-Personal/works/CellChat/tutorial/data_humanSkin_CellChat.rda")
data.input = data_humanSkin$data # normalized data matrix
meta = data_humanSkin$meta # a dataframe with rownames containing cell mata data
cell.use = rownames(meta)[meta$condition == "LS"] # extract the cell names from disease data

# Subset the input data for CelChat analysis
data.input = data.input[, cell.use]
meta = meta[cell.use, ]
# meta = data.frame(labels = meta$labels[cell.use], row.names = colnames(data.input)) # manually create a dataframe consisting of the cell labels
unique(meta$labels) # check the cell labels
#>  [1] Inflam. FIB  FBN1+ FIB    APOE+ FIB    COL11A1+ FIB cDC2        
#>  [6] LC           Inflam. DC   cDC1         CD40LG+ TC   Inflam. TC  
#> [11] TC           NKT         
#> 12 Levels: APOE+ FIB FBN1+ FIB COL11A1+ FIB Inflam. FIB cDC1 cDC2 ... NKT
```

### (B) Starting from a Seurat object

```
data.input <- seurat_object[["RNA"]]@data # normalized data matrix
# For Seurat version >= “5.0.0”, get the normalized data via `seurat_object[["RNA"]]$data`
labels <- Idents(seurat_object)
meta <- data.frame(labels = labels, row.names = names(labels)) # create a dataframe of the cell labels
```

### (C) Starting from a SingleCellExperiment object

```
data.input <- SingleCellExperiment::logcounts(object) # normalized data matrix
meta <- as.data.frame(SingleCellExperiment::colData(object)) # extract a dataframe of the cell labels
meta$labels <- meta[["sce.clusters"]]
```

### (D) Starting from an Anndata object

```
# read the data into R using anndata R package
install.packages("anndata")
library(anndata)
ad <- read_h5ad("scanpy_object.h5ad")
# access count data matrix
counts <- t(as.matrix(ad$X))
# normalize the count data if the normalized data is not available in the .h5ad file
library.size <- Matrix::colSums(counts)
data.input <- as(log1p(Matrix::t(Matrix::t(counts)/library.size) * 10000), "dgCMatrix")
# access meta data
meta <- ad$obs 
meta$labels <- meta[["ad_clusters"]]
```

## Create a CellChat object

**USERS can create a new CellChat object from a data matrix,
Seurat, SingleCellExperiment or AnnData object.** If input is a
Seurat or SingleCellExperiment object, the meta data in the object will
be used by default and USER must provide `group.by` to define
the cell groups. e.g, group.by = “ident” for the default cell identities
in Seurat object.

Create a CellChat object by following option A when taking the
digital gene expression matrix and cell label information as input,
option B when taking a Seurat object as input, option C when taking a
SingleCellExperiment object as input, and option D when taking a AnnData
object as input.

**NB: If USERS load previously calculated CellChat object
(version < 0.5.0), please update the object via
`updateCellChat`**

### (A) Starting from the digital gene expression matrix and cell label information

```
cellchat <- createCellChat(object = data.input, meta = meta, group.by = "labels")
#> [1] "Create a CellChat object from a data matrix"
#> Set cell identities for the new CellChat object 
#> The cell groups used for CellChat analysis are  APOE+ FIB, FBN1+ FIB, COL11A1+ FIB, Inflam. FIB, cDC1, cDC2, LC, Inflam. DC, TC, Inflam. TC, CD40LG+ TC, NKT
```

### (B) Starting from a Seurat object

```
cellChat <- createCellChat(object = seurat.obj, group.by = "ident", assay = "RNA")
```

### (C) Starting from a SingleCellExperiment object

```
cellChat <- createCellChat(object = sce.obj, group.by = "sce.clusters")
```

### (D) Starting from an AnnData object

```
sce <- zellkonverter::readH5AD(file = "adata.h5ad")
# retrieve all the available assays within sce object
assayNames(sce)
# added a new assay entry "logcounts" if not available
counts <- assay(sce, "X") # make sure this is the original count data matrix
library.size <- Matrix::colSums(counts)
logcounts(sce) <- log1p(Matrix::t(Matrix::t(counts)/library.size) * 10000)
# extract a cell meta data
meta <- as.data.frame(SingleCellExperiment::colData(sce)) #
cellChat <- createCellChat(object = sce, group.by = "sce.clusters")
```

If cell mata information is not added when creating CellChat object,
USERS can also add it later using `addMeta`, and set the
default cell identities using `setIdent`.

```
cellchat <- addMeta(cellchat, meta = meta)
cellchat <- setIdent(cellchat, ident.use = "labels") # set "labels" as default cell identity
levels(cellchat@idents) # show factor levels of the cell labels
groupSize <- as.numeric(table(cellchat@idents)) # number of cells in each cell group
```

## Set the ligand-receptor interaction database

Before users can employ CellChat to infer cell-cell communication,
they need to set the ligand-receptor interaction database and identify
over-expressed ligands or receptors.

Our database CellChatDB is a manually curated database of
literature-supported ligand-receptor interactions in both human and
mouse. CellChatDB v2 contains ~3,300 validated molecular interactions,
including ~40% of secrete autocrine/paracrine signaling interactions,
~17% of extracellular matrix (ECM)-receptor interactions, ~13% of
cell-cell contact interactions and ~30% non-protein signaling. Compared
to CellChatDB v1, CellChatDB v2 adds more than 1000 protein and
non-protein interactions such as metabolic and synaptic signaling. It
should be noted that for molecules that are not directly related to
genes measured in scRNA-seq, CellChat v2 estimates the expression of
ligands and receptors using those molecules’ key mediators or enzymes
for potential communication mediated by non-proteins.

CellChatDB v2 also adds additional functional annotations of
ligand-receptor pairs, such as UniProtKB keywords (including biological
process, molecular function, functional class, disease, etc),
subcellular location and relevance to neurotransmitter.

Users can update CellChatDB by adding their own curated
ligand-receptor pairs. Please check the tutorial
on updating the ligand-receptor interaction database CellChatDB.

When analyzing human samples, use the database
**`CellChatDB.human`**; when analyzing mouse
samples, use the database
**`CellChatDB.mouse`**. CellChatDB categorizes
ligand-receptor pairs into different types, including “Secreted
Signaling”, “ECM-Receptor”, “Cell-Cell Contact” and “Non-protein
Signaling”. By default, the “Non-protein Signaling” are not used.

```
CellChatDB <- CellChatDB.human # use CellChatDB.mouse if running on mouse data
showDatabaseCategory(CellChatDB)
```

```
# Show the structure of the database
dplyr::glimpse(CellChatDB$interaction)
#> Rows: 3,234
#> Columns: 28
#> $ interaction_name         <chr> "TGFB1_TGFBR1_TGFBR2", "TGFB2_TGFBR1_TGFBR2",…
#> $ pathway_name             <chr> "TGFb", "TGFb", "TGFb", "TGFb", "TGFb", "TGFb…
#> $ ligand                   <chr> "TGFB1", "TGFB2", "TGFB3", "TGFB1", "TGFB1", …
#> $ receptor                 <chr> "TGFbR1_R2", "TGFbR1_R2", "TGFbR1_R2", "ACVR1…
#> $ agonist                  <chr> "TGFb agonist", "TGFb agonist", "TGFb agonist…
#> $ antagonist               <chr> "TGFb antagonist", "TGFb antagonist", "TGFb a…
#> $ co_A_receptor            <chr> "", "", "", "", "", "", "", "", "", "", "", "…
#> $ co_I_receptor            <chr> "TGFb inhibition receptor", "TGFb inhibition …
#> $ evidence                 <chr> "KEGG: hsa04350", "KEGG: hsa04350", "KEGG: hs…
#> $ annotation               <chr> "Secreted Signaling", "Secreted Signaling", "…
#> $ interaction_name_2       <chr> "TGFB1 - (TGFBR1+TGFBR2)", "TGFB2 - (TGFBR1+T…
#> $ is_neurotransmitter      <lgl> FALSE, FALSE, FALSE, FALSE, FALSE, FALSE, FAL…
#> $ ligand.symbol            <chr> "TGFB1", "TGFB2", "TGFB3", "TGFB1", "TGFB1", …
#> $ ligand.family            <chr> "TGF-beta", "TGF-beta", "TGF-beta", "TGF-beta…
#> $ ligand.location          <chr> "Extracellular matrix, Secreted, Extracellula…
#> $ ligand.keyword           <chr> "Disease variant, Signal, Reference proteome,…
#> $ ligand.secreted_type     <chr> "growth factor", "growth factor", "cytokine;g…
#> $ ligand.transmembrane     <lgl> FALSE, FALSE, TRUE, FALSE, FALSE, FALSE, FALS…
#> $ receptor.symbol          <chr> "TGFBR2, TGFBR1", "TGFBR2, TGFBR1", "TGFBR2, …
#> $ receptor.family          <chr> "Protein kinase superfamily, TKL Ser/Thr prot…
#> $ receptor.location        <chr> "Cell membrane, Secreted, Membrane raft, Cell…
#> $ receptor.keyword         <chr> "Membrane, Secreted, Disulfide bond, Kinase, …
#> $ receptor.surfaceome_main <chr> "Receptors", "Receptors", "Receptors", "Recep…
#> $ receptor.surfaceome_sub  <chr> "Act.TGFB;Kinase", "Act.TGFB;Kinase", "Act.TG…
#> $ receptor.adhesome        <chr> "", "", "", "", "", "", "", "", "", "", "", "…
#> $ receptor.secreted_type   <chr> "", "", "", "", "", "", "", "", "", "", "", "…
#> $ receptor.transmembrane   <lgl> TRUE, TRUE, TRUE, TRUE, TRUE, TRUE, TRUE, TRU…
#> $ version                  <chr> "CellChatDB v1", "CellChatDB v1", "CellChatDB…

# use a subset of CellChatDB for cell-cell communication analysis
CellChatDB.use <- subsetDB(CellChatDB, search = "Secreted Signaling", key = "annotation") # use Secreted Signaling

# Only uses the Secreted Signaling from CellChatDB v1
#  CellChatDB.use <- subsetDB(CellChatDB, search = list(c("Secreted Signaling"), c("CellChatDB v1")), key = c("annotation", "version"))

# use all CellChatDB except for "Non-protein Signaling" for cell-cell communication analysis
# CellChatDB.use <- subsetDB(CellChatDB)


# use all CellChatDB for cell-cell communication analysis
# CellChatDB.use <- CellChatDB # simply use the default CellChatDB. We do not suggest to use it in this way because CellChatDB v2 includes "Non-protein Signaling" (i.e., metabolic and synaptic signaling). 

# set the used database in the object
cellchat@DB <- CellChatDB.use
```

## Preprocessing the expression data for cell-cell communication analysis

To infer the cell state-specific communications, CellChat identifies
over-expressed ligands or receptors in one cell group and then
identifies over-expressed ligand-receptor interactions if either ligand
or receptor are over-expressed.

We also provide a function to project gene expression data onto
protein-protein interaction (PPI) network. Specifically, a diffusion
process is used to smooth genes’ expression values based on their
neighbors’ defined in a high-confidence experimentally validated
protein-protein network. This function is useful when analyzing
single-cell data with shallow sequencing depth because the projection
reduces the dropout effects of signaling genes, in particular for
possible zero expression of subunits of ligands/receptors. One might be
concerned about the possible artifact introduced by this diffusion
process, however, it will only introduce very weak communications. By
default CellChat uses the raw data (i.e.,
`object@data.signaling`) instead of the projected data. To
use the projected data, users should run the function
`projectData` before running `computeCommunProb`,
and then set `raw.use = FALSE` when running
`computeCommunProb`.

```
# subset the expression data of signaling genes for saving computation cost
cellchat <- subsetData(cellchat) # This step is necessary even if using the whole database
future::plan("multisession", workers = 4) # do parallel
cellchat <- identifyOverExpressedGenes(cellchat)
cellchat <- identifyOverExpressedInteractions(cellchat)
#> The number of highly variable ligand-receptor pairs used for signaling inference is 692

execution.time = Sys.time() - ptm
print(as.numeric(execution.time, units = "secs"))
#> [1] 13.20763
# project gene expression data onto PPI (Optional: when running it, USER should set `raw.use = FALSE` in the function `computeCommunProb()` in order to use the projected data)
# cellchat <- projectData(cellchat, PPI.human)
```

# Part II: Inference of cell-cell communication network

CellChat infers the biologically significant cell-cell communication
by assigning each interaction with a probability value and peforming a
permutation test. CellChat models the probability of cell-cell
communication by integrating gene expression with prior known knowledge
of the interactions between signaling ligands, receptors and their
cofactors using the law of mass action.

CAUTION: The number of inferred ligand-receptor pairs clearly depends
on the **method for calculating the average gene expression per
cell group**. By default, CellChat uses a statistically robust
mean method called ‘trimean’, which produces fewer interactions than
other methods. However, we find that CellChat performs well at
predicting stronger interactions, which is very helpful for narrowing
down on interactions for further experimental validations. In
`computeCommunProb`, we provide an option for using other
methods, such as 5% and 10% truncated mean, to calculating the average
gene expression. Of note, ‘trimean’ approximates 25% truncated mean,
implying that the average gene expression is zero if the percent of
expressed cells in one group is less than 25%. To use 10% truncated
mean, USER can set `type = "truncatedMean"` and
`trim = 0.1`. To determine a proper value of trim, CellChat
provides a function `computeAveExpr`, which can help to check
the average expression of signaling genes of interest, e.g,
`computeAveExpr(cellchat, features = c("CXCL12","CXCR4"), type = "truncatedMean", trim = 0.1)`.
Therefore, if well-known signaling pathways in the studied biological
process are not predicted, users can try `truncatedMean` with
lower values of `trim` to change the method for calculating
the average gene expression per cell group.

When analyzing unsorted single-cell transcriptomes, under the
assumption that abundant cell populations tend to send collectively
stronger signals than the rare cell populations, CellChat can also
consider the effect of cell proportion in each cell group in the
probability calculation. USER can set
`population.size = TRUE`.

## Compute the communication probability and infer cellular communication network

```
ptm = Sys.time()
cellchat <- computeCommunProb(cellchat, type = "triMean")
#> triMean is used for calculating the average gene expression per cell group. 
#> [1] ">>> Run CellChat on sc/snRNA-seq data <<< [2024-02-14 00:32:35.767285]"
#> [1] ">>> CellChat inference is done. Parameter values are stored in `object@options$parameter` <<< [2024-02-14 00:33:13.121225]"
```

The key parameter for this analysis is `type`, the method
for computing the average gene expression per cell group. By default
`type = "triMean"`, producing fewer but stronger
interactions. When setting `type = "truncatedMean"`, a value
should be assigned to `trim`, producing more interactions.
Please check above in detail on the **method for calculating the
average gene expression per cell group**.

Users can filter out the cell-cell communication if there are only
few cells in certain cell groups. By default, the minimum number of
cells required in each cell group for cell-cell communication is 10.

```
cellchat <- filterCommunication(cellchat, min.cells = 10)
```

## Extract the inferred cellular communication network as a data frame

We provide a function `subsetCommunication` to easily
access the inferred cell-cell communications of interest. For
example,

- `df.net <- subsetCommunication(cellchat)` returns a
  data frame consisting of all the inferred cell-cell communications at
  the level of ligands/receptors. Set `slot.name = "netP"` to
  access the the inferred communications at the level of signaling
  pathways
- `df.net <- subsetCommunication(cellchat, sources.use = c(1,2), targets.use = c(4,5))`
  gives the inferred cell-cell communications sending from cell groups 1
  and 2 to cell groups 4 and 5.
- `df.net <- subsetCommunication(cellchat, signaling = c("WNT", "TGFb"))`
  gives the inferred cell-cell communications mediated by signaling WNT
  and TGFb.

## Infer the cell-cell communication at a signaling pathway level

CellChat computes the communication probability on signaling pathway
level by summarizing the communication probabilities of all
ligands-receptors interactions associated with each signaling
pathway.

NB: The inferred intercellular communication network of each
ligand-receptor pair and each signaling pathway is stored in the slot
‘net’ and ‘netP’, respectively.

```
cellchat <- computeCommunProbPathway(cellchat)
```

## Calculate the aggregated cell-cell communication network

CellChat calculates the aggregated cell-cell communication network by
counting the number of links or summarizing the communication
probability. Users can also calculate the aggregated network among a
subset of cell groups by setting `sources.use` and
`targets.use`.

```
cellchat <- aggregateNet(cellchat)
execution.time = Sys.time() - ptm
print(as.numeric(execution.time, units = "secs"))
#> [1] 38.73308
```

CellChat can also visualize the aggregated cell-cell communication
network. For example, showing the number of interactions or the total
interaction strength (weights) between any two cell groups using circle
plot.

```
ptm = Sys.time()
groupSize <- as.numeric(table(cellchat@idents))
par(mfrow = c(1,2), xpd=TRUE)
netVisual_circle(cellchat@net$count, vertex.weight = groupSize, weight.scale = T, label.edge= F, title.name = "Number of interactions")
netVisual_circle(cellchat@net$weight, vertex.weight = groupSize, weight.scale = T, label.edge= F, title.name = "Interaction weights/strength")
```

Due to the complicated cell-cell communication network, we can examine
the signaling sent from each cell group. Here we also control the
parameter `edge.weight.max` so that we can compare edge
weights between differet networks.

```
mat <- cellchat@net$weight
par(mfrow = c(3,4), xpd=TRUE)
for (i in 1:nrow(mat)) {
  mat2 <- matrix(0, nrow = nrow(mat), ncol = ncol(mat), dimnames = dimnames(mat))
  mat2[i, ] <- mat[i, ]
  netVisual_circle(mat2, vertex.weight = groupSize, weight.scale = T, edge.weight.max = max(mat), title.name = rownames(mat)[i])
}
```

# Part III: Visualization of cell-cell communication network

Upon infering the cell-cell communication network, CellChat provides
various functionality for further data exploration, analysis, and
visualization. Specifically:

- It provides several ways for visualizing cell-cell communication
  network, including hierarchical plot, circle plot, Chord diagram, and
  bubble plot.
- It provides an easy-to-use tool for extracting and visualizing
  high-order information of the inferred networks. For example, it allows
  ready prediction of major signaling inputs and outputs for cell
  populations and how these populations and signals coordinate together
  for functions.
- It can quantitatively characterize and compare the inferred
  cell-cell communication networks using an integrated approach by
  combining social network analysis, pattern recognition, and manifold
  learning approaches.

## Visualize each signaling pathway using Hierarchy plot, Circle plot or Chord diagram

**Hierarchy plot**: USER should define
`vertex.receiver`, which is a numeric vector giving the index
of the cell groups as targets in the left part of hierarchy plot. This
hierarchical plot consist of two components: the left portion shows
autocrine and paracrine signaling to certain cell groups of interest
(i.e, the defined `vertex.receiver`), and the right portion
shows autocrine and paracrine signaling to the remaining cell groups in
the dataset. Thus, hierarchy plot provides an informative and intuitive
way to visualize autocrine and paracrine signaling communications
between cell groups of interest. For example, when studying the
cell-cell communication between fibroblasts and immune cells, USER can
define `vertex.receiver` as all fibroblast cell groups.

**Chord diagram**: CellChat provides two functions
`netVisual_chord_cell` and `netVisual_chord_gene`
for visualizing cell-cell communication with different purposes and
different levels. `netVisual_chord_cell` is used for
visualizing the cell-cell communication between different cell groups
(where each sector in the chord diagram is a cell group), and
`netVisual_chord_gene` is used for visualizing the cell-cell
communication mediated by mutiple ligand-receptors or signaling pathways
(where each sector in the chord diagram is a ligand, receptor or
signaling pathway.)

**Explnations of edge color/weight, node
color/size/shape**: In all visualization plots, edge colors are
consistent with the sources as sender, and edge weights are proportional
to the interaction strength. Thicker edge line indicates a stronger
signal. In the **Hierarchy plot and Circle plot**, circle
sizes are proportional to the number of cells in each cell group. In the
hierarchy plot, solid and open circles represent source and target,
respectively. In the **Chord diagram**, the inner thinner
bar colors represent the targets that receive signal from the
corresponding outer bar. The inner bar size is proportional to the
signal strength received by the targets. Such inner bar is helpful for
interpreting the complex chord diagram. Note that there exist some inner
bars without any chord for some cell groups, please just igore it
because this is an issue that has not been addressed by circlize package.

**Visualization of cell-cell communication at different
levels**: One can visualize the inferred communication network of
signaling pathways using `netVisual_aggregate`, and visualize
the inferred communication networks of individual L-R pairs associated
with that signaling pathway using `netVisual_individual`.

Here we take input of one signaling pathway as an example. All the
signaling pathways showing significant communications can be accessed by
`cellchat@netP$pathways`.

```
pathways.show <- c("CXCL") 
# Hierarchy plot
# Here we define `vertex.receive` so that the left portion of the hierarchy plot shows signaling to fibroblast and the right portion shows signaling to immune cells 
vertex.receiver = seq(1,4) # a numeric vector. 
netVisual_aggregate(cellchat, signaling = pathways.show,  vertex.receiver = vertex.receiver)
# Circle plot
par(mfrow=c(1,1))
netVisual_aggregate(cellchat, signaling = pathways.show, layout = "circle")
```

```
# Chord diagram
par(mfrow=c(1,1))
netVisual_aggregate(cellchat, signaling = pathways.show, layout = "chord")
```

```
# Heatmap
par(mfrow=c(1,1))
netVisual_heatmap(cellchat, signaling = pathways.show, color.heatmap = "Reds")
#> Do heatmap based on a single object
```

For the chord diagram, CellChat has an independent function
`netVisual_chord_cell` to flexibly visualize the signaling
network by adjusting different parameters in the circlize package. For
example, we can define a named char vector `group` to create
multiple-group chord diagram, e.g., grouping cell clusters into
different cell types.

```
# Chord diagram
group.cellType <- c(rep("FIB", 4), rep("DC", 4), rep("TC", 4)) # grouping cell clusters into fibroblast, DC and TC cells
names(group.cellType) <- levels(cellchat@idents)
netVisual_chord_cell(cellchat, signaling = pathways.show, group = group.cellType, title.name = paste0(pathways.show, " signaling network"))
#> Plot the aggregated cell-cell communication network at the signaling pathway level
```

### Compute the contribution of each ligand-receptor pair to the overall signaling pathway and visualize cell-cell communication mediated by a single ligand-receptor pair

```
netAnalysis_contribution(cellchat, signaling = pathways.show)
```

We can also visualize the cell-cell communication mediated by a
single ligand-receptor pair. We provide a function
`extractEnrichedLR` to extract all the significant
interactions (L-R pairs) and related signaling genes for a given
signaling pathway.

```
pairLR.CXCL <- extractEnrichedLR(cellchat, signaling = pathways.show, geneLR.return = FALSE)
LR.show <- pairLR.CXCL[1,] # show one ligand-receptor pair
# Hierarchy plot
vertex.receiver = seq(1,4) # a numeric vector
netVisual_individual(cellchat, signaling = pathways.show,  pairLR.use = LR.show, vertex.receiver = vertex.receiver)
#> [[1]]
# Circle plot
netVisual_individual(cellchat, signaling = pathways.show, pairLR.use = LR.show, layout = "circle")
```

```
#> [[1]]
# Chord diagram
netVisual_individual(cellchat, signaling = pathways.show, pairLR.use = LR.show, layout = "chord")
```

```
#> [[1]]
```

### Automatically save the plots of the all inferred network for quick exploration

In practical use, USERS can use ‘for … loop’ to automatically save
the all inferred network for quick exploration using
`netVisual`. `netVisual` supports an output in the
formats of svg, png and pdf.

```
# Access all the signaling pathways showing significant communications
pathways.show.all <- cellchat@netP$pathways
# check the order of cell identity to set suitable vertex.receiver
levels(cellchat@idents)
vertex.receiver = seq(1,4)
for (i in 1:length(pathways.show.all)) {
  # Visualize communication network associated with both signaling pathway and individual L-R pairs
  netVisual(cellchat, signaling = pathways.show.all[i], vertex.receiver = vertex.receiver, layout = "hierarchy")
  # Compute and visualize the contribution of each ligand-receptor pair to the overall signaling pathway
  gg <- netAnalysis_contribution(cellchat, signaling = pathways.show.all[i])
  ggsave(filename=paste0(pathways.show.all[i], "_L-R_contribution.pdf"), plot=gg, width = 3, height = 2, units = 'in', dpi = 300)
}
```

## Visualize cell-cell communication mediated by multiple ligand-receptors or signaling pathways

CellChat can also show all the significant interactions mediated by
L-R pairs and signaling pathways, and interactions provided by users
from some cell groups to other cell groups using the function
netVisual\_bubble (option A) and netVisual\_chord\_gene (option B).

### (A) Bubble plot

We can also show all the significant interactions (L-R pairs) from
some cell groups to other cell groups using
`netVisual_bubble`.

```
# (1) show all the significant interactions (L-R pairs) from some cell groups (defined by 'sources.use') to other cell groups (defined by 'targets.use')
netVisual_bubble(cellchat, sources.use = 4, targets.use = c(5:11), remove.isolate = FALSE)
#> Comparing communications on a single object
```

```
# (2) show all the significant interactions (L-R pairs) associated with certain signaling pathways
netVisual_bubble(cellchat, sources.use = 4, targets.use = c(5:11), signaling = c("CCL","CXCL"), remove.isolate = FALSE)
#> Comparing communications on a single object
```

```
# (3) show all the significant interactions (L-R pairs) based on user's input (defined by `pairLR.use`)
pairLR.use <- extractEnrichedLR(cellchat, signaling = c("CCL","CXCL","FGF"))
netVisual_bubble(cellchat, sources.use = c(3,4), targets.use = c(5:8), pairLR.use = pairLR.use, remove.isolate = TRUE)
#> Comparing communications on a single object
```

```
# set the order of interacting cell pairs on x-axis
# (4) Default: first sort cell pairs based on the appearance of sources in levels(object@idents), and then based on the appearance of targets in levels(object@idents)
# (5) sort cell pairs based on the targets.use defined by users
netVisual_bubble(cellchat, targets.use = c("LC","Inflam. DC","cDC2","CD40LG+ TC"), pairLR.use = pairLR.use, remove.isolate = TRUE, sort.by.target = T)
# (6) sort cell pairs based on the sources.use defined by users
netVisual_bubble(cellchat, sources.use = c("FBN1+ FIB","APOE+ FIB","Inflam. FIB"), pairLR.use = pairLR.use, remove.isolate = TRUE, sort.by.source = T)
# (7) sort cell pairs based on the sources.use and then targets.use defined by users
netVisual_bubble(cellchat, sources.use = c("FBN1+ FIB","APOE+ FIB","Inflam. FIB"), targets.use = c("LC","Inflam. DC","cDC2","CD40LG+ TC"), pairLR.use = pairLR.use, remove.isolate = TRUE, sort.by.source = T, sort.by.target = T)
# (8) sort cell pairs based on the targets.use and then sources.use defined by users
netVisual_bubble(cellchat, sources.use = c("FBN1+ FIB","APOE+ FIB","Inflam. FIB"), targets.use = c("LC","Inflam. DC","cDC2","CD40LG+ TC"), pairLR.use = pairLR.use, remove.isolate = TRUE, sort.by.source = T, sort.by.target = T, sort.by.source.priority = FALSE)
```

### (B) Chord diagram

Similar to Bubble plot, CellChat provides a function
`netVisual_chord_gene` for drawing Chord diagram to

- show all the interactions (L-R pairs or signaling pathways) from
  some cell groups to other cell groups. Two special cases: one is showing
  all the interactions sending from one cell groups and the other is
  showing all the interactions received by one cell group.
- show the interactions inputted by USERS or certain signaling
  pathways defined by USERS

```
# show all the significant interactions (L-R pairs) from some cell groups (defined by 'sources.use') to other cell groups (defined by 'targets.use')
# show all the interactions sending from Inflam.FIB
netVisual_chord_gene(cellchat, sources.use = 4, targets.use = c(5:11), lab.cex = 0.5,legend.pos.y = 30)
```

```
# show all the interactions received by Inflam.DC
netVisual_chord_gene(cellchat, sources.use = c(1,2,3,4), targets.use = 8, legend.pos.x = 15)
```

```
# show all the significant interactions (L-R pairs) associated with certain signaling pathways
netVisual_chord_gene(cellchat, sources.use = c(1,2,3,4), targets.use = c(5:11), signaling = c("CCL","CXCL"),legend.pos.x = 8)
```

```
# show all the significant signaling pathways from some cell groups (defined by 'sources.use') to other cell groups (defined by 'targets.use')
netVisual_chord_gene(cellchat, sources.use = c(1,2,3,4), targets.use = c(5:11), slot.name = "netP", legend.pos.x = 10)
```

NB: Please ignore the note when generating the plot such as “Note: The
first link end is drawn out of sector ‘MIF’.”. If the gene names are
overlapped, you can adjust the argument `small.gap` by
decreasing the value.

## Plot the signaling gene expression distribution using violin/dot plot

CellChat can plot the gene expression distribution of signaling genes
related to L-R pairs or signaling pathways using a Seurat wrapper
function `plotGeneExpression` if the Seurat R package has
been installed. This function provides three types of visualizztion,
including “violin”, “dot”, “bar”. Alternatively, users can extract the
signaling genes related to the inferred L-R pairs or signaling pathway
using `extractEnrichedLR`, and then plot gene expression
using Seurat or other packages.

```
plotGeneExpression(cellchat, signaling = "CXCL", enriched.only = TRUE, type = "violin")
#> The legacy packages maptools, rgdal, and rgeos, underpinning the sp package,
#> which was just loaded, were retired in October 2023.
#> Please refer to R-spatial evolution reports for details, especially
#> https://r-spatial.org/r/2023/05/15/evolution4.html.
#> It may be desirable to make the sf package available;
#> package maintainers should consider adding sf to Suggests:.
#> Scale for y is already present.
#> Adding another scale for y, which will replace the existing scale.
#> Scale for y is already present.
#> Adding another scale for y, which will replace the existing scale.
#> Scale for y is already present.
#> Adding another scale for y, which will replace the existing scale.
```

```
print(as.numeric(execution.time, units = "secs"))
#> [1] 38.73308
```

By default, `plotGeneExpression` only shows the expression
of signaling genes related to the inferred significant communications.
USERS can show the expression of all signaling genes related to one
signaling pathway by

```
plotGeneExpression(cellchat, signaling = "CXCL", enriched.only = FALSE)
execution.time = Sys.time() - ptm
```

# Part IV: Systems analysis of cell-cell communication network

To facilitate the interpretation of the complex intercellular
communication networks, CellChat quantitively measures networks through
methods abstracted from graph theory, pattern recognition and manifold
learning.

- It can determine major signaling sources and targets as well as
  mediators and influencers within a given signaling network using
  centrality measures from network analysis
- It can predict key incoming and outgoing signals for specific
  cell types as well as coordinated responses among different cell types
  by leveraging pattern recognition approaches.
- It can group signaling pathways by defining similarity measures
  and performing manifold learning from both functional and topological
  perspectives.
- It can delineate conserved and context-specific signaling
  pathways by joint manifold learning of multiple networks.

## Identify signaling roles (e.g., dominant senders, receivers) of cell groups as well as the major contributing signaling

CellChat allows ready identification of **dominant senders,
receivers, mediators and influencers** in the intercellular
communication network by computing several network centrality measures
for each cell group. Specifically, we used measures in weighted-directed
networks, including **out-degree, in-degree, flow betweenesss and
information centrality**, to respectively identify dominant
senders, receivers, mediators and influencers for the intercellular
communications. In a weighteddirected network with the weights as the
computed communication probabilities, the outdegree, computed as the sum
of communication probabilities of the outgoing signaling from a cell
group, and the in-degree, computed as the sum of the communication
probabilities of the incoming signaling to a cell group, can be used to
identify the dominant cell senders and receivers of signaling networks,
respectively. For the definition of flow betweenness and information
centrality, please check our published paper and related reference.

Users can visualize the centrality scores on a heatmap (option A) and
a 2D plot (option B). CellChat can also answer the question on which
signals contribute the most to outgoing or incoming signaling of certain
cell groups (option C).

### (A) Compute and visualize the network centrality scores

```
ptm = Sys.time()
# Compute the network centrality scores
cellchat <- netAnalysis_computeCentrality(cellchat, slot.name = "netP") # the slot 'netP' means the inferred intercellular communication network of signaling pathways
# Visualize the computed centrality scores using heatmap, allowing ready identification of major signaling roles of cell groups
netAnalysis_signalingRole_network(cellchat, signaling = pathways.show, width = 8, height = 2.5, font.size = 10)
```

### (B) Visualize dominant senders (sources) and receivers (targets) in a 2D space

CellChat also provides another intuitive way to visualize the
dominant senders (sources) and receivers (targets) in a 2D space using
scatter plot. x-axis and y-axis are respectively the total outgoing or
incoming communication probability associated with each cell group. Dot
size is proportional to the number of inferred links (both outgoing and
incoming) associated with each cell group. Dot colors indicate different
cell groups. Dot shapes indicate different categories of cell groups if
`group` is defined.

```
# Signaling role analysis on the aggregated cell-cell communication network from all signaling pathways
gg1 <- netAnalysis_signalingRole_scatter(cellchat)
#> Signaling role analysis on the aggregated cell-cell communication network from all signaling pathways
# Signaling role analysis on the cell-cell communication networks of interest
gg2 <- netAnalysis_signalingRole_scatter(cellchat, signaling = c("CXCL", "CCL"))
#> Signaling role analysis on the cell-cell communication network from user's input
gg1 + gg2
```

### (C) Identify signals contributing the most to outgoing or incoming signaling of certain cell groups

We can also answer the question on which signals contributing most to
outgoing or incoming signaling of certain cell groups. In this heatmap,
colobar represents the relative signaling strength of a signaling
pathway across cell groups (NB: values are row-scaled). The top colored
bar plot shows the total signaling strength of a cell group by
summarizing all signaling pathways displayed in the heatmap. The right
grey bar plot shows the total signaling strength of a signaling pathway
by summarizing all cell groups displayed in the heatmap.

```
# Signaling role analysis on the aggregated cell-cell communication network from all signaling pathways
ht1 <- netAnalysis_signalingRole_heatmap(cellchat, pattern = "outgoing")
ht2 <- netAnalysis_signalingRole_heatmap(cellchat, pattern = "incoming")
ht1 + ht2
```

```
# Signaling role analysis on the cell-cell communication networks of interest
ht <- netAnalysis_signalingRole_heatmap(cellchat, signaling = c("CXCL", "CCL"))
```

## Identify global communication patterns to explore how multiple cell types and signaling pathways coordinate together

In addition to exploring detailed communications for individual
pathways, an important question is how multiple cell groups and
signaling pathways coordinate to function. CellChat employs a pattern
recognition method to identify the global communication patterns.

As the number of patterns increases, there might be redundant
patterns, making it difficult to interpret the communication patterns.
We chose five patterns as default. Generally, it is biologically
meaningful with the number of patterns greater than 2. In addition, we
also provide a function `selectK` to infer the number of
patterns, which is based on two metrics that have been implemented in
the NMF R package, including Cophenetic and Silhouette. Both metrics
measure the stability for a particular number of patterns based on a
hierarchical clustering of the consensus matrix. For a range of the
number of patterns, a suitable number of patterns is the one at which
Cophenetic and Silhouette values begin to drop suddenly.

This analysis can be done for outgoing (option A) and incoming
(option B) signaling patterns. Outgoing patterns reveal how the sender
cells (i.e., cells as signal source) coordinate with each other as well
as how they coordinate with certain signaling pathways to drive
communication. Incoming patterns show how the target cells (i.e., cells
as signal receivers) coordinate with each other as well as how they
coordinate with certain signaling pathways to respond to incoming
signals.

### (A) Identify and visualize outgoing communication pattern of secreting cells

**Outgoing patterns** reveal how the sender cells
(i.e. cells as signal source) coordinate with each other as well as how
they coordinate with certain signaling pathways to drive
communication.

For outgoing (or incoming) patterns, the cell group pattern matrix W
outputted from the matrix factorization of outgoing (or incoming)
cell-cell communication probability indicates how these cell groups
coordinate to send (or receive) signals and the signaling pathway
pattern matrix H indicates how these signaling pathways work together to
send (or receive) signals. To intuitively show the associations of
latent patterns with cell groups and ligand-receptor pairs or signaling
pathways, we used a **river (alluvial) plot**. We first
normalized each row of W and each column of H to be [0,1], and then set
the elements in W and H to be zero if they are less than a threshold (by
default: 0.5). Such thresholding allows to uncover the most enriched
cell groups and signaling pathways associated with each inferred
pattern. These thresholded matrices W and H are used as inputs for
creating an alluvial plot.

Moreover, to directly relate cell groups with their enriched
signaling pathways, we set the elements in W and H to be zero if they
are less than a threshold (by default: 1/R) where R is the number of
latent patterns. By using a less strict threshold, more enriched
signaling pathways associated each cell group might be obtained. Using a
contribution score of each cell group to each signaling pathway computed
by multiplying W by H, we constructed a **dot plot** in
which the dot size is proportion to the contribution score to show
association between cell group and their enriched signaling pathways.
Users can also decrease the parameter `cutoff` to show more
enriched signaling pathways associated each cell group.

Load required package for the communication pattern analysis

```
library(NMF)
#> Loading required package: registry
#> Loading required package: rngtools
#> Loading required package: cluster
#> NMF - BioConductor layer [OK] | Shared memory capabilities [NO: bigmemory] | Cores 2/2
#>   To enable shared memory capabilities, try: install.extras('
#> NMF
#> ')
#> 
#> Attaching package: 'NMF'
#> The following objects are masked from 'package:igraph':
#> 
#>     algorithm, compare
library(ggalluvial)
```

Here we run `selectK` to infer the number of patterns.

```
selectK(cellchat, pattern = "outgoing")
```

Both Cophenetic and Silhouette values begin to drop suddenly when the
number of outgoing patterns is 6.

```
nPatterns = 6
cellchat <- identifyCommunicationPatterns(cellchat, pattern = "outgoing", k = nPatterns)
```

```
# river plot
netAnalysis_river(cellchat, pattern = "outgoing")
#> Please make sure you have load `library(ggalluvial)` when running this function
```

```
# dot plot
netAnalysis_dot(cellchat, pattern = "outgoing")
```

### (B) Identify and visualize incoming communication pattern of target cells

**Incoming patterns** show how the target cells
(i.e. cells as signal receivers) coordinate with each other as well as
how they coordinate with certain signaling pathways to respond to
incoming signals.

```
selectK(cellchat, pattern = "incoming")
```

Cophenetic values begin to drop when the number of incoming patterns is
3.

```
nPatterns = 3
cellchat <- identifyCommunicationPatterns(cellchat, pattern = "incoming", k = nPatterns)
```

```
# river plot
netAnalysis_river(cellchat, pattern = "incoming")
#> Please make sure you have load `library(ggalluvial)` when running this function
```

```
# dot plot
netAnalysis_dot(cellchat, pattern = "incoming")
```

## Manifold and classification learning analysis of signaling networks

Further, CellChat is able to quantify the similarity between all
significant signaling pathways and then group them based on their
cellular communication network similarity. Grouping can be done either
based on the functional or structural similarity.

**Functional similarity**: High degree of functional
similarity indicates major senders and receivers are similar, and it can
be interpreted as the two signaling pathways or two ligand-receptor
pairs exhibit similar and/or redundant roles. **The functional
similarity analysis requires the same cell population composition
between two datasets.**

**Structural similarity**: A structural similarity was
used to compare their signaling network structure, without considering
the similarity of senders and receivers.

### Identify signaling groups based on their functional similarity

```
cellchat <- computeNetSimilarity(cellchat, type = "functional")
cellchat <- netEmbedding(cellchat, type = "functional")
#> Manifold learning of the signaling networks for a single dataset
cellchat <- netClustering(cellchat, type = "functional")
#> Classification learning of the signaling networks for a single dataset
# Visualization in 2D-space
netVisual_embedding(cellchat, type = "functional", label.size = 3.5)
```

```
# netVisual_embeddingZoomIn(cellchat, type = "functional", nCol = 2)
```

### Identify signaling groups based on structure similarity

```
cellchat <- computeNetSimilarity(cellchat, type = "structural")
cellchat <- netEmbedding(cellchat, type = "structural")
#> Manifold learning of the signaling networks for a single dataset
cellchat <- netClustering(cellchat, type = "structural")
#> Classification learning of the signaling networks for a single dataset
# Visualization in 2D-space
netVisual_embedding(cellchat, type = "structural", label.size = 3.5)
```

```
netVisual_embeddingZoomIn(cellchat, type = "structural", nCol = 2)
```

```
execution.time = Sys.time() - ptm
print(as.numeric(execution.time, units = "secs"))
#> [1] 147.8175
```

# Part V: Save the CellChat object

```
saveRDS(cellchat, file = "cellchat_humanSkin_LS.rds")
```

# Part VI: Explore the cell-cell communication through the Interactive CellChat Explorer

For CellChat analysis of single-cell transcriptomics, please make
sure the `object@dr` contains a low-dimensional space of the
data such as “umap” and “tsne” in order to produce the feature plot of
signaling genes. A new reduced space can be add via the function
`addReduction`.

```
runCellChatApp(cellchat)
```

```
sessionInfo()
#> R version 4.3.1 (2023-06-16)
#> Platform: aarch64-apple-darwin20 (64-bit)
#> Running under: macOS Ventura 13.5
#> 
#> Matrix products: default
#> BLAS:   /Library/Frameworks/R.framework/Versions/4.3-arm64/Resources/lib/libRblas.0.dylib 
#> LAPACK: /Library/Frameworks/R.framework/Versions/4.3-arm64/Resources/lib/libRlapack.dylib;  LAPACK version 3.11.0
#> 
#> locale:
#> [1] en_US.UTF-8/en_US.UTF-8/en_US.UTF-8/C/en_US.UTF-8/en_US.UTF-8
#> 
#> time zone: Asia/Shanghai
#> tzcode source: internal
#> 
#> attached base packages:
#> [1] parallel  stats     graphics  grDevices utils     datasets  methods  
#> [8] base     
#> 
#> other attached packages:
#>  [1] doParallel_1.0.17   iterators_1.0.14    foreach_1.5.2      
#>  [4] ggalluvial_0.12.5   NMF_0.26            cluster_2.1.4      
#>  [7] rngtools_1.5.2      registry_0.5-1      patchwork_1.1.3    
#> [10] CellChat_2.1.2      Biobase_2.60.0      BiocGenerics_0.46.0
#> [13] ggplot2_3.4.3       igraph_1.5.1        dplyr_1.1.3        
#> 
#> loaded via a namespace (and not attached):
#>   [1] RcppAnnoy_0.0.21       splines_4.3.1          later_1.3.1           
#>   [4] tibble_3.2.1           polyclip_1.10-4        ggnetwork_0.5.12      
#>   [7] fastDummies_1.7.3      lifecycle_1.0.3        rstatix_0.7.2.999     
#>  [10] rprojroot_2.0.3        globals_0.16.2         lattice_0.21-8        
#>  [13] MASS_7.3-60            backports_1.4.1        magrittr_2.0.3        
#>  [16] plotly_4.10.2          sass_0.4.7             rmarkdown_2.24        
#>  [19] jquerylib_0.1.4        yaml_2.3.7             httpuv_1.6.11         
#>  [22] Seurat_5.0.1           sctransform_0.4.1      spam_2.9-1            
#>  [25] spatstat.sparse_3.0-2  sp_2.1-0               reticulate_1.31       
#>  [28] cowplot_1.1.1          pbapply_1.7-2          RColorBrewer_1.1-3    
#>  [31] abind_1.4-5            Rtsne_0.16             purrr_1.0.2           
#>  [34] presto_1.0.0           circlize_0.4.16        IRanges_2.34.1        
#>  [37] S4Vectors_0.38.1       ggrepel_0.9.3          irlba_2.3.5.1         
#>  [40] spatstat.utils_3.0-3   listenv_0.9.0          goftest_1.2-3         
#>  [43] RSpectra_0.16-1        spatstat.random_3.1-5  fitdistrplus_1.1-11   
#>  [46] parallelly_1.36.0      svglite_2.1.1          leiden_0.4.3          
#>  [49] codetools_0.2-19       tidyselect_1.2.0       shape_1.4.6           
#>  [52] farver_2.1.1           matrixStats_1.0.0      stats4_4.3.1          
#>  [55] spatstat.explore_3.2-1 jsonlite_1.8.7         GetoptLong_1.0.5      
#>  [58] BiocNeighbors_1.18.0   ellipsis_0.3.2         progressr_0.14.0      
#>  [61] ggridges_0.5.4         survival_3.5-7         systemfonts_1.0.4     
#>  [64] tools_4.3.1            ragg_1.2.5             sna_2.7-1             
#>  [67] ica_1.0-3              Rcpp_1.0.11            glue_1.6.2            
#>  [70] gridExtra_2.3          here_1.0.1             xfun_0.40             
#>  [73] withr_2.5.0            BiocManager_1.30.22    fastmap_1.1.1         
#>  [76] fansi_1.0.4            digest_0.6.33          R6_2.5.1              
#>  [79] mime_0.12              textshaping_0.3.6      colorspace_2.1-0      
#>  [82] scattermore_1.2        tensor_1.5             spatstat.data_3.0-1   
#>  [85] utf8_1.2.3             tidyr_1.3.0            generics_0.1.3        
#>  [88] data.table_1.14.9      FNN_1.1.3.2            httr_1.4.7            
#>  [91] htmlwidgets_1.6.2      uwot_0.1.16            pkgconfig_2.0.3       
#>  [94] gtable_0.3.4           ComplexHeatmap_2.15.4  lmtest_0.9-40         
#>  [97] htmltools_0.5.6        carData_3.0-5          dotCall64_1.0-2       
#> [100] clue_0.3-64            SeuratObject_5.0.1     scales_1.2.1          
#> [103] tidyverse_2.0.0        png_0.1-8              knitr_1.43            
#> [106] rstudioapi_0.15.0      reshape2_1.4.4         rjson_0.2.21          
#> [109] nlme_3.1-163           coda_0.19-4            statnet.common_4.9.0  
#> [112] cachem_1.0.8           zoo_1.8-12             GlobalOptions_0.1.2   
#> [115] stringr_1.5.0          KernSmooth_2.23-22     miniUI_0.1.1.1        
#> [118] pillar_1.9.0           grid_4.3.1             vctrs_0.6.3           
#> [121] RANN_2.6.1             promises_1.2.1         ggpubr_0.6.0          
#> [124] car_3.1-2              xtable_1.8-4           evaluate_0.21         
#> [127] magick_2.8.1           cli_3.6.1              compiler_4.3.1        
#> [130] rlang_1.1.1            crayon_1.5.2           future.apply_1.11.0   
#> [133] ggsignif_0.6.4         labeling_0.4.3         plyr_1.8.8            
#> [136] stringi_1.7.12         deldir_1.0-9           viridisLite_0.4.2     
#> [139] network_1.18.1         gridBase_0.4-7         BiocParallel_1.34.2   
#> [142] munsell_0.5.0          lazyeval_0.2.2         spatstat.geom_3.2-4   
#> [145] Matrix_1.6-5           RcppHNSW_0.5.0         future_1.33.0         
#> [148] shiny_1.7.5            highr_0.10             ROCR_1.0-11           
#> [151] broom_1.0.5            bslib_0.5.1
```
