# Supplementary material for: CSNK2B Expression Aligns with a GDF15-Associated Immune Molecular State in Hepatocellular Carcinoma
Source: Biomedicines. 2026 Aug 26;14(9):1909. doi: 10.3390/biomedicines14091909 (PMC13604900; doi:10.3390/biomedicines14091909)
Supplement: Supplementary file 1 [file biomedicines-14-01909-s001.zip › Supplemental file S1/docker_cellchat/CellChat-main-http1.tar.gz!/CellChat-main/tutorial/CellChat_analysis_of_multiple_spatial_transcriptomics_datasets.html]

CellChat analysis of multiple spatial transcriptomics datasets


# CellChat analysis of multiple spatial transcriptomics datasets

#### Suoqin Jin

#### 22 February, 2024

- Load the required
  libraries
- Part
  I: Data input & processing and initialization of CellChat object
  - Load data
  - Create a CellChat object
  - Set the
    ligand-receptor interaction database
  - Preprocessing
    the expression data for cell-cell communication analysis
- Part II:
  Inference of cell-cell communication network
  - Compute
    the communication probability and infer cellular communication
    network
  - Infer
    the cell-cell communication at a signaling pathway level
  - Calculate
    the aggregated cell-cell communication network
- Part
  III: Visualization of cell-cell communication network
  - Compute
    the contribution of each ligand-receptor pair to the overall signaling
    pathway
- Part V: Save the CellChat
  object

This vignette outlines the steps of inference, analysis and
visualization of cell-cell communication network for **multiple
spatial transcriptomics datasets using CellChat**. We showcase
CellChat’s application to multiple spatial transcriptomics datasets by
applying it to two replicates from human spatial intestine datasets,
which were downloaded from https://simmonslab.shinyapps.io/FetalAtlasDataPortal/.

Below we briefly describe the key steps of applying CellChat to
multiple spatial transcriptomics datasets. Please check the vignette
of applying CellChat to an individual spatially resolved transcriptomics
dataset for detailed descriptions of the methods and steps, and
check the vignette
of FAQ on applying CellChat to spatially resolved transcriptomics
data for detailed descriptions of applying CellChat to different
types of spatial transcriptomics data.

## Load the required libraries

```
ptm = Sys.time()
library(CellChat)
library(patchwork)
```

# Part I: Data input & processing and initialization of CellChat object

## Load data

```
# Here we load two Seurat objects of two replicates from 10X Visium human intestine dataset
library(Seurat)
#> Loading required package: SeuratObject
#> Loading required package: sp
#> The legacy packages maptools, rgdal, and rgeos, underpinning the sp package,
#> which was just loaded, were retired in October 2023.
#> Please refer to R-spatial evolution reports for details, especially
#> https://r-spatial.org/r/2023/05/15/evolution4.html.
#> It may be desirable to make the sf package available;
#> package maintainers should consider adding sf to Suggests:.
#> 'SeuratObject' was built with package 'Matrix' 1.6.3 but the current
#> version is 1.6.5; it is recomended that you reinstall 'SeuratObject' as
#> the ABI for 'Matrix' may have changed
#> 
#> Attaching package: 'SeuratObject'
#> The following object is masked from 'package:BiocGenerics':
#> 
#>     intersect
#> The following object is masked from 'package:base':
#> 
#>     intersect
#> 
#> Attaching package: 'Seurat'
#> The following object is masked from 'package:igraph':
#> 
#>     components
seu1 <- readRDS("/Users/suoqinjin/Library/CloudStorage/OneDrive-Personal/works/CellChat/tutorial/Spatial_A1_adult_with_predictions.RDS")
seu1
#> An object of class Seurat 
#> 16005 features across 2649 samples within 4 assays 
#> Active assay: SCT (15882 features, 3000 variable features)
#>  3 layers present: counts, data, scale.data
#>  3 other assays present: Spatial, fetal.predictions, adult.predictions
#>  2 dimensional reductions calculated: pca, umap
#>  1 image present: slice1
seu2 <- readRDS("/Users/suoqinjin/Library/CloudStorage/OneDrive-Personal/works/CellChat/tutorial/Spatial_A2_adult_with_predictions.RDS")
seu2
#> An object of class Seurat 
#> 50173 features across 2316 samples within 4 assays 
#> Active assay: SCT (16514 features, 3000 variable features)
#>  3 layers present: counts, data, scale.data
#>  3 other assays present: Spatial, fetal.predictions, adult.predictions
#>  2 dimensional reductions calculated: pca, umap
#>  1 image present: slice1

# assign label to each spot based on the maximum predicted probabilities
assignLabels <- function(object, prediction = "predictions") {
  pred <- object[[prediction]]@data
  pred <- pred[1:(nrow(pred)-1), ]
  # label each spot based on the maximum prediction probability
  labels = rownames(pred)[apply(pred, 2, which.max)]
  names(labels) <- colnames(pred)
  object$labels <- factor(labels)
  Idents(object) <- "labels"
  return(object)
}
seu1 <- assignLabels(seu1, prediction = "adult.predictions")
seu2 <- assignLabels(seu2, prediction = "adult.predictions")

# show the image and annotated spots
color.use <- scPalette(nlevels(seu1)); names(color.use) <- levels(seu1)
p1 <- Seurat::SpatialDimPlot(seu1, label = F, label.size = 3, cols = color.use)
color.use <- scPalette(nlevels(seu2)); names(color.use) <- levels(seu2)
p2 <- Seurat::SpatialDimPlot(seu2, label = F, label.size = 3, cols = color.use) + NoLegend()
p1 + p2
```

```
# Prepare input data for CelChat analysis
data.input1 = Seurat::GetAssayData(seu1, slot = "data", assay = "SCT") # normalized data matrix
#> Warning: The `slot` argument of `GetAssayData()` is deprecated as of SeuratObject 5.0.0.
#> ℹ Please use the `layer` argument instead.
#> This warning is displayed once every 8 hours.
#> Call `lifecycle::last_lifecycle_warnings()` to see where this warning was
#> generated.
data.input2 = Seurat::GetAssayData(seu2, slot = "data", assay = "SCT") 

genes.common <- intersect(rownames(data.input1), rownames(data.input2))
colnames(data.input1) <- paste0("A1_", colnames(data.input1))
colnames(data.input2) <- paste0("A2_", colnames(data.input2))
data.input <- cbind(data.input1[genes.common, ], data.input2[genes.common, ])

# define the meta data
# a column named `samples` should be provided for spatial transcriptomics analysis, which is useful for analyzing cell-cell communication by aggregating multiple samples/replicates. Of note, for comparison analysis across different conditions, users still need to create a CellChat object seperately for each condition.  
meta1 = data.frame(labels = Idents(seu1), samples = "A1") # manually create a dataframe consisting of the cell labels
meta2 = data.frame(labels = Idents(seu2), samples = "A2") 

meta <- rbind(meta1, meta2)
rownames(meta) <- colnames(data.input)
# a factor level should be defined for the `meta$labels` and `meta$samples`
meta$labels <- factor(meta$labels, levels = levels(Idents(seu1)))
meta$samples <- factor(meta$samples, levels = c("A1", "A2"))
unique(meta$labels) # check the cell labels
#>  [1] Myofibroblasts        Cycling Cells         Undifferentiated     
#>  [4] Glial                 DC2                   B-Cells              
#>  [7] Goblets               T-Cells               Plasma Cells         
#> [10] Colonocytes           Crypt Top Colonocytes ILCs                 
#> [13] Mast Cells            BEST4+/OTOP2+ Cell    NK                   
#> [16] Endothelial 2         Enteroendocrines      Stromal 3            
#> [19] Stromal 1             Stromal 2             Pericytes            
#> [22] Endothelial 1         DC1                   Macrophages&Monocytes
#> [25] Stromal 4            
#> 25 Levels: B-Cells BEST4+/OTOP2+ Cell Colonocytes ... Undifferentiated
unique(meta$samples) # check the sample labels
#> [1] A1 A2
#> Levels: A1 A2

# load spatial transcriptomics information
# Spatial locations of spots from full (NOT high/low) resolution images are required. For 10X Visium, this information is in `tissue_positions.csv`. 
spatial.locs1 = Seurat::GetTissueCoordinates(seu1, scale = NULL, cols = c("imagerow", "imagecol")) 
spatial.locs2 = Seurat::GetTissueCoordinates(seu2, scale = NULL, cols = c("imagerow", "imagecol")) 
spatial.locs <- rbind(spatial.locs1, spatial.locs2)
rownames(spatial.locs) <- colnames(data.input)

# Scale factors of spatial coordinates
# For 10X Visium, the conversion factor of converting spatial coordinates from Pixels to Micrometers can be computed as the ratio of the theoretical spot size (i.e., 65um) over the number of pixels that span the diameter of a theoretical spot size in the full-resolution image (i.e., 'spot_diameter_fullres' in pixels in the 'scalefactors_json.json' file). 
# Of note, the 'spot_diameter_fullres' factor is different from the `spot` in Seurat object and thus users still need to get the value from the original json file. 
scalefactors1 = jsonlite::fromJSON(txt = file.path("/Users/suoqinjin/Library/CloudStorage/OneDrive-Personal/works/CellChat/tutorial/spatial_imaging_data-intestinalA1", 'scalefactors_json.json'))
spot.size = 65 # the theoretical spot size (um) in 10X Visium
conversion.factor1 = spot.size/scalefactors1$spot_diameter_fullres
spatial.factors1 = data.frame(ratio = conversion.factor1, tol = spot.size/2)

scalefactors2 = jsonlite::fromJSON(txt = file.path("/Users/suoqinjin/Library/CloudStorage/OneDrive-Personal/works/CellChat/tutorial/spatial_imaging_data-intestinalA2", 'scalefactors_json.json'))
conversion.factor2 = spot.size/scalefactors2$spot_diameter_fullres
spatial.factors2 = data.frame(ratio = conversion.factor2, tol = spot.size/2)

spatial.factors <- rbind(spatial.factors1, spatial.factors2)
rownames(spatial.factors) <- c("A1", "A2")
```

## Create a CellChat object

**NB: If USERS load previously calculated CellChat object
(version < 2.1.0), please update the object via
`updateCellChat`**

```
cellchat <- createCellChat(object = data.input, meta = meta, group.by = "labels",
                           datatype = "spatial", coordinates = spatial.locs, spatial.factors = spatial.factors)
#> [1] "Create a CellChat object from a data matrix"
#> Create a CellChat object from spatial transcriptomics data... 
#> Set cell identities for the new CellChat object 
#> The cell groups used for CellChat analysis are  B-Cells, BEST4+/OTOP2+ Cell, Colonocytes, Crypt Top Colonocytes, Cycling Cells, DC1, DC2, Endothelial 1, Endothelial 2, Enteroendocrines, Glial, Goblets, ILCs, Macrophages&Monocytes, Mast Cells, Myofibroblasts, NK, Pericytes, Plasma Cells, Stromal 1, Stromal 2, Stromal 3, Stromal 4, T-Cells, Undifferentiated
cellchat
#> An object of class CellChat created from a single dataset 
#>  15609 genes.
#>  4965 cells. 
#> CellChat analysis of spatial data! The input spatial locations are 
#>                       x_cent y_cent
#> A1_AAACAAGTATCTCCCA-1   4372   5303
#> A1_AAACAGAGCGACTCCT-1   1753   4960
#> A1_AAACATTTCCCGGATT-1   5173   5097
#> A1_AAACCACTACACAGAT-1    949   5919
#> A1_AAACCCGAACGAAATC-1   4006   5846
#> A1_AAACCGGAAATGTTAA-1   4660   6225
```

## Set the ligand-receptor interaction database

```
CellChatDB <- CellChatDB.human # use CellChatDB.human if running on human data

# use a subset of CellChatDB for cell-cell communication analysis
CellChatDB.use <- subsetDB(CellChatDB, search = "Secreted Signaling", key = "annotation") # use Secreted Signaling
# set the used database in the object
cellchat@DB <- CellChatDB.use
```

## Preprocessing the expression data for cell-cell communication analysis

To infer the cell state-specific communications, we identify
over-expressed ligands or receptors in one cell group and then identify
over-expressed ligand-receptor interactions if either ligand or receptor
is over-expressed.

```
# subset the expression data of signaling genes for saving computation cost
cellchat <- subsetData(cellchat) # This step is necessary even if using the whole database
future::plan("multisession", workers = 4) 
cellchat <- identifyOverExpressedGenes(cellchat)
cellchat <- identifyOverExpressedInteractions(cellchat)
#> The number of highly variable ligand-receptor pairs used for signaling inference is 422
 
execution.time = Sys.time() - ptm
print(as.numeric(execution.time, units = "secs"))
#> [1] 20.40818
```

# Part II: Inference of cell-cell communication network

## Compute the communication probability and infer cellular communication network

```
ptm = Sys.time()
cellchat <- computeCommunProb(cellchat, type = "truncatedMean", trim = 0.1, 
                              distance.use = FALSE, interaction.range = 250, scale.distance = NULL,
                              contact.dependent = TRUE, contact.range = 100)
#> truncatedMean is used for calculating the average gene expression per cell group. 
#> [1] ">>> Run CellChat on spatial transcriptomics data without distance values as constraints of the computed communication probability <<< [2024-02-22 12:06:15.985272]"
#> Molecules of the input L-R pairs are diffusible. Run CellChat in a diffusion manner based on the `interaction.range`.
#> [1] ">>> CellChat inference is done. Parameter values are stored in `object@options$parameter` <<< [2024-02-22 12:09:10.370361]"
```

Users can filter out the cell-cell communication if there are only
few cells in certain cell groups. By default, the minimum number of
cells required in each cell group for cell-cell communication is 10.

```
cellchat <- filterCommunication(cellchat, min.cells = 10)
#> The cell-cell communication related with the following cell groups are excluded due to the few number of cells:  Stromal 4 !     0.0% interactions are removed!
```

## Infer the cell-cell communication at a signaling pathway level

CellChat computes the communication probability on signaling pathway
level by summarizing the communication probabilities of all
ligands-receptors interactions associated with each signaling
pathway.

NB: The inferred intercellular communication network of each
ligand-receptor pair and each signaling pathway is stored in the slot
‘net’ and ‘netP’, respectively.

```
cellchat <- computeCommunProbPathway(cellchat)
```

## Calculate the aggregated cell-cell communication network

We can calculate the aggregated cell-cell communication network by
counting the number of links or summarizing the communication
probability.

```
cellchat <- aggregateNet(cellchat)

execution.time = Sys.time() - ptm
print(as.numeric(execution.time, units = "secs"))
#> [1] 188.3582
```

We can also visualize the aggregated cell-cell communication network.
For example, showing the number of interactions or the total interaction
strength (weights) between any two cell groups using circle plot or
heatmap plot.

```
ptm = Sys.time()

groupSize <- as.numeric(table(cellchat@idents))
par(mfrow = c(1,2), xpd=TRUE)
netVisual_circle(cellchat@net$count, vertex.weight = rowSums(cellchat@net$count), weight.scale = T, label.edge= F, title.name = "Number of interactions")
netVisual_circle(cellchat@net$weight, vertex.weight = rowSums(cellchat@net$weight), weight.scale = T, label.edge= F, title.name = "Interaction weights/strength")
```

```
netVisual_heatmap(cellchat, measure = "count", color.heatmap = "Blues")
#> Do heatmap based on a single object
```

```
#netVisual_heatmap(cellchat, measure = "weight", color.heatmap = "Blues")
```

# Part III: Visualization of cell-cell communication network

Upon infering the cell-cell communication network, CellChat provides
various functionality for further data exploration, analysis, and
visualization. Here we only showcase the `circle plot` and
the new `spatial plot`.

**Visualization of cell-cell communication at different
levels**: One can visualize the inferred communication network of
signaling pathways using `netVisual_aggregate`, and visualize
the inferred communication networks of individual L-R pairs associated
with that signaling pathway using `netVisual_individual`.

Here we take input of one signaling pathway as an example. All the
signaling pathways showing significant communications can be accessed by
`cellchat@netP$pathways`.

```
pathways.show <- c("EGF") 
# Circle plot
par(mfrow=c(1,1), xpd=TRUE)
netVisual_aggregate(cellchat, signaling = pathways.show, layout = "circle")
```

```
# Spatial plot
par(mfrow=c(1,1))
netVisual_aggregate(cellchat, signaling = pathways.show, sample.use = "A1", layout = "spatial", edge.width.max = 2, vertex.size.max = 1, alpha.image = 0.2, vertex.label.cex = 0)
```

```
# Setting `vertex.label.cex = 0` to hide the labels on the spatial plot
execution.time = Sys.time() - ptm
print(as.numeric(execution.time, units = "secs"))
#> [1] 1.140493
```

Compute and visualize the network centrality scores:

```
# Compute the network centrality scores
cellchat <- netAnalysis_computeCentrality(cellchat, slot.name = "netP") # the slot 'netP' means the inferred intercellular communication network of signaling pathways
# Visualize the computed centrality scores using heatmap, allowing ready identification of major signaling roles of cell groups
par(mfrow=c(1,1))
netAnalysis_signalingRole_network(cellchat, signaling = pathways.show, width = 8, height = 2.5, font.size = 10)
```

```
# USER can show this information on the spatial transcriptomics when visualizing a signaling network, e.g., bigger circle indicates larger incoming signaling
par(mfrow=c(1,1))
netVisual_aggregate(cellchat, signaling = pathways.show, sample.use = "A1", layout = "spatial", edge.width.max = 2, alpha.image = 0.2, vertex.weight = "incoming", vertex.size.max = 6, vertex.label.cex = 0)
```

### Compute the contribution of each ligand-receptor pair to the overall signaling pathway

```
netAnalysis_contribution(cellchat, signaling = pathways.show)
```

When visualizing gene expression distribution on tissue using
`spatialFeaturePlot`, users also need to provide the
`sample.use` as an input.

```
# Take an input of a few genes
spatialFeaturePlot(cellchat, features = c("AREG","EGFR"), sample.use = "A1", point.size = 0.8, color.heatmap = "Reds", direction = 1)
```

```
spatialFeaturePlot(cellchat, features = c("AREG","EGFR"), sample.use = "A2",point.size = 0.8, color.heatmap = "Reds", direction = 1)
```

```
# Take an input of a ligand-receptor pair
spatialFeaturePlot(cellchat, pairLR.use = "AREG_EGFR", sample.use = "A1", point.size = 0.5, do.binary = FALSE, cutoff = 0.05, enriched.only = F, color.heatmap = "Reds", direction = 1)
#> Applying a cutoff of  0.05 to the values...
```

```
# Take an input of a ligand-receptor pair and show expression in binary
spatialFeaturePlot(cellchat, pairLR.use = "AREG_EGFR", sample.use = "A1", point.size = 1.5, do.binary = TRUE, cutoff = 0.05, enriched.only = F, color.heatmap = "Reds", direction = 1)
```

**NB: Upon infering the intercellular communication network
from spatial transcriptomics data, CellChat’s various functionality can
be used for further data exploration, analysis, and visualization.
Please check other functionalities in the basic
tutorial of CellChat and comparison
analysis across different conditions**

# Part V: Save the CellChat object

```
saveRDS(cellchat, file = "cellchat_human_intestine_ReplicatesA1A2.rds")
```

```
sessionInfo()
#> R version 4.3.1 (2023-06-16)
#> Platform: aarch64-apple-darwin20 (64-bit)
#> Running under: macOS Ventura 13.5
#> 
#> Matrix products: default
#> BLAS:   /Library/Frameworks/R.framework/Versions/4.3-arm64/Resources/lib/libRblas.0.dylib 
#> LAPACK: /Library/Frameworks/R.framework/Versions/4.3-arm64/Resources/lib/libRlapack.dylib;  LAPACK version 3.11.0
#> 
#> locale:
#> [1] en_US.UTF-8/en_US.UTF-8/en_US.UTF-8/C/en_US.UTF-8/en_US.UTF-8
#> 
#> time zone: Asia/Shanghai
#> tzcode source: internal
#> 
#> attached base packages:
#> [1] stats     graphics  grDevices utils     datasets  methods   base     
#> 
#> other attached packages:
#>  [1] Seurat_5.0.1        SeuratObject_5.0.1  sp_2.1-0           
#>  [4] patchwork_1.1.3     CellChat_2.1.2      Biobase_2.60.0     
#>  [7] BiocGenerics_0.46.0 ggplot2_3.4.3       igraph_1.5.1       
#> [10] dplyr_1.1.3        
#> 
#> loaded via a namespace (and not attached):
#>   [1] RcppAnnoy_0.0.21       splines_4.3.1          later_1.3.1           
#>   [4] tibble_3.2.1           polyclip_1.10-4        ggnetwork_0.5.12      
#>   [7] fastDummies_1.7.3      lifecycle_1.0.3        rstatix_0.7.2.999     
#>  [10] doParallel_1.0.17      globals_0.16.2         lattice_0.21-8        
#>  [13] MASS_7.3-60            backports_1.4.1        magrittr_2.0.3        
#>  [16] plotly_4.10.2          sass_0.4.7             rmarkdown_2.24        
#>  [19] jquerylib_0.1.4        yaml_2.3.7             httpuv_1.6.11         
#>  [22] NMF_0.26               sctransform_0.4.1      spam_2.9-1            
#>  [25] spatstat.sparse_3.0-2  reticulate_1.31        cowplot_1.1.1         
#>  [28] pbapply_1.7-2          RColorBrewer_1.1-3     abind_1.4-5           
#>  [31] Rtsne_0.16             purrr_1.0.2            presto_1.0.0          
#>  [34] circlize_0.4.16        IRanges_2.34.1         S4Vectors_0.38.1      
#>  [37] ggrepel_0.9.3          irlba_2.3.5.1          listenv_0.9.0         
#>  [40] spatstat.utils_3.0-3   goftest_1.2-3          RSpectra_0.16-1       
#>  [43] spatstat.random_3.1-5  fitdistrplus_1.1-11    parallelly_1.36.0     
#>  [46] svglite_2.1.1          leiden_0.4.3           codetools_0.2-19      
#>  [49] tidyselect_1.2.0       shape_1.4.6            farver_2.1.1          
#>  [52] matrixStats_1.0.0      stats4_4.3.1           spatstat.explore_3.2-1
#>  [55] jsonlite_1.8.7         GetoptLong_1.0.5       BiocNeighbors_1.18.0  
#>  [58] ellipsis_0.3.2         progressr_0.14.0       ggridges_0.5.4        
#>  [61] ggalluvial_0.12.5      survival_3.5-7         iterators_1.0.14      
#>  [64] systemfonts_1.0.4      foreach_1.5.2          tools_4.3.1           
#>  [67] sna_2.7-1              ica_1.0-3              Rcpp_1.0.11           
#>  [70] glue_1.6.2             gridExtra_2.3          xfun_0.40             
#>  [73] withr_2.5.0            BiocManager_1.30.22    fastmap_1.1.1         
#>  [76] fansi_1.0.4            digest_0.6.33          R6_2.5.1              
#>  [79] mime_0.12              colorspace_2.1-0       scattermore_1.2       
#>  [82] tensor_1.5             spatstat.data_3.0-1    utf8_1.2.3            
#>  [85] tidyr_1.3.0            generics_0.1.3         data.table_1.14.9     
#>  [88] FNN_1.1.3.2            httr_1.4.7             htmlwidgets_1.6.2     
#>  [91] uwot_0.1.16            pkgconfig_2.0.3        gtable_0.3.4          
#>  [94] registry_0.5-1         ComplexHeatmap_2.15.4  lmtest_0.9-40         
#>  [97] htmltools_0.5.6        carData_3.0-5          dotCall64_1.0-2       
#> [100] clue_0.3-64            scales_1.2.1           tidyverse_2.0.0       
#> [103] png_0.1-8              knitr_1.43             rstudioapi_0.15.0     
#> [106] reshape2_1.4.4         rjson_0.2.21           nlme_3.1-163          
#> [109] coda_0.19-4            statnet.common_4.9.0   cachem_1.0.8          
#> [112] zoo_1.8-12             GlobalOptions_0.1.2    stringr_1.5.0         
#> [115] KernSmooth_2.23-22     parallel_4.3.1         miniUI_0.1.1.1        
#> [118] pillar_1.9.0           grid_4.3.1             vctrs_0.6.3           
#> [121] RANN_2.6.1             promises_1.2.1         ggpubr_0.6.0          
#> [124] car_3.1-2              xtable_1.8-4           cluster_2.1.4         
#> [127] evaluate_0.21          magick_2.8.1           cli_3.6.1             
#> [130] compiler_4.3.1         rlang_1.1.1            crayon_1.5.2          
#> [133] rngtools_1.5.2         future.apply_1.11.0    ggsignif_0.6.4        
#> [136] labeling_0.4.3         plyr_1.8.8             stringi_1.7.12        
#> [139] deldir_1.0-9           viridisLite_0.4.2      network_1.18.1        
#> [142] gridBase_0.4-7         BiocParallel_1.34.2    munsell_0.5.0         
#> [145] lazyeval_0.2.2         spatstat.geom_3.2-4    Matrix_1.6-5          
#> [148] RcppHNSW_0.5.0         future_1.33.0          shiny_1.7.5           
#> [151] highr_0.10             ROCR_1.0-11            broom_1.0.5           
#> [154] bslib_0.5.1
```
