# Supplementary material for: CSNK2B Expression Aligns with a GDF15-Associated Immune Molecular State in Hepatocellular Carcinoma
Source: Biomedicines. 2026 Aug 26;14(9):1909. doi: 10.3390/biomedicines14091909 (PMC13604900; doi:10.3390/biomedicines14091909)
Supplement: Supplementary file 1 [file biomedicines-14-01909-s001.zip › Supplemental file S1/docker_cellchat/CellChat-main-http1.tar.gz!/CellChat-main/tutorial/CellChat_analysis_of_spatial_transcriptomics_data.html]

CellChat inference and analysis of spatially proximal cell-cell communication from spatially resolved transcriptomics


# CellChat inference and analysis of spatially proximal cell-cell communication from spatially resolved transcriptomics

#### Suoqin Jin

#### 22 February, 2024

- Load the required
  libraries
- Part
  I: Data input & processing and initialization of CellChat object
  - Load data
  - Create a CellChat object
  - Set the
    ligand-receptor interaction database
  - Preprocessing
    the expression data for cell-cell communication analysis
- Part II:
  Inference of cell-cell communication network
  - Compute
    the communication probability and infer cellular communication
    network
  - Extract
    the inferred cellular communication network as a data frame
  - Infer
    the cell-cell communication at a signaling pathway level
  - Calculate
    the aggregated cell-cell communication network
- Part
  III: Visualization of cell-cell communication network
- Part V: Save the CellChat
  object
- Part
  VI: Explore the cell-cell communication through the Interactive CellChat
  Explorer
- Part
  VII: Application to different technologies of spatially-resolved
  transcriptomics

This vignette outlines the steps of inference, analysis and
visualization of cell-cell communication network for **a single
spatial transcriptomics dataset using CellChat**. We showcase
CellChat’s application to spatial transcriptomics data by applying it to
a mouse brain 10X visium dataset (https://www.10xgenomics.com/resources/datasets/mouse-brain-serial-section-1-sagittal-anterior-1-standard-1-0-0).
Biological annotations of spots (i.e., cell group information) are
predicted using Seurat (https://satijalab.org/seurat/articles/spatial\_vignette.html).

CellChat requires gene expression and spatial location data of
spots/cells as the user input and models the probability of cell-cell
communication by integrating gene expression with spatial distance as
well as prior knowledge of the interactions between signaling ligands,
receptors and their cofactors.

Upon infering the intercellular communication network, CellChat’s
various functionality can be used for further data exploration,
analysis, and visualization.

## Load the required libraries

```
ptm = Sys.time()

library(CellChat)
library(patchwork)
options(stringsAsFactors = FALSE)
```

# Part I: Data input & processing and initialization of CellChat object

When inferring spatially-proximal cell-cell communication from
spatially resolved transcriptomic data, user also should provide spatial
coordinates/locations of spot/cell centroids. In addition, to filter out
cell-cell communication beyond the maximum diffusion range of molecules
(e.g., ~250μm), CellChat needs to compute the cell centroid-to-centroid
distance in the unit of micrometers. Therefore, for spatial technologies
that only provide spatial coordinates in pixels, CellChat converts
spatial coordinates from pixels to micrometers by requiring users to
input the conversion factor.

CellChat requires four user inputs:

- **`data.input` (Gene expression data of
  spots/cells)**: genes should be in rows with rownames and cells
  in columns with colnames. Normalized data (e.g., library-size
  normalization and then log-transformed with a pseudocount of 1) is
  required as input for CellChat analysis. If user provides count data, we
  provide a `normalizeData` function to account for library
  size and then do log-transformed.
- **`meta` (User assigned cell labels and samples
  labels)**: a data frame (rows are cells with rownames) consisting
  of cell information, which will be used for defining cell groups. A
  column named `samples` should be provided for spatial
  transcriptomics analysis, which is useful for analyzing cell-cell
  communication by aggregating multiple samples/replicates. Of note, for
  comparison analysis across different conditions, users still need to
  create a CellChat object seperately for each condition.
- **`coordinates` (Spatial coordinates of
  spots/cells)**: a data frame in which each row gives the spatial
  coordinates/locations of each cell/spot centroid.
- **`spatial.factors` (Spatial factors of spatial
  distance)**: a data frame containing two distance factors
  `ratio` and `tol`, which is dependent on spatial
  transcriptomics technologies (and specific datasets). (i)
  `ratio`: the conversion factor when converting spatial
  coordinates from Pixels or other units to Micrometers (i.e.,Microns).
  For example, setting `ratio = 0.18` indicates that 1 pixel
  equals 0.18um in the coordinates. (ii) `tol`: the tolerance
  factor to increase the robustness when comparing the center-to-center
  distance against the `interaction.range`. This can be the
  half value of cell/spot size in the unit of um. **Please check the
  vignette
  of FAQ on applying CellChat to spatially resolved transcriptomics
  data for detailed explanations and setting of different technologies
  of spatial transcriptomics data.**

When inferring contact-dependent or juxtacrine signaling by setting
`contact.dependent = TRUE` in `computeCommunProb`,
and using L-R pairs from `Cell-Cell Contact` signaling
classified in `CellChatDB$interaction$annotation`, CellChat
requires another one user input:

- **`contact.range`**: a value giving the
  interaction range (Unit: microns) to restrict the contact-dependent
  signaling. For spatial transcriptomics in a single-cell resolution,
  `contact.range` is approximately equal to the estimated cell
  diameter (i.e., the cell center-to-center distance), which means that
  contact-dependent or juxtacrine signaling can only happens when the two
  cells are contact to each other. Typically,
  `contact.range = 10`, which is a typical human cell size.
  However, for low-resolution spatial data such as 10X visium, it should
  be the cell center-to-center distance (i.e.,
  `contact.range = 100` for 10X visium data). The function
  `computeCellDistance` can compute the center-to-center
  distance.

Instead of providing `contact.range`, users may
alternatively provide a value of `contact.knn.k`, in order to
restrict the contact-dependent signaling within the k-nearest neighbors
(knn).

## Load data

```
# Here we load a Seurat object of 10X Visium mouse cortex data and its associated cell meta data
load("/Users/suoqinjin/Library/CloudStorage/OneDrive-Personal/works/CellChat/tutorial/visium_mouse_cortex_annotated_full.RData")
# show the image and annotated spots
color.use <- scPalette(nlevels(visium.brain)); names(color.use) <- levels(visium.brain)
#> Loading required package: SeuratObject
#> Loading required package: sp
#> The legacy packages maptools, rgdal, and rgeos, underpinning the sp package,
#> which was just loaded, were retired in October 2023.
#> Please refer to R-spatial evolution reports for details, especially
#> https://r-spatial.org/r/2023/05/15/evolution4.html.
#> It may be desirable to make the sf package available;
#> package maintainers should consider adding sf to Suggests:.
#> 'SeuratObject' was built with package 'Matrix' 1.6.3 but the current
#> version is 1.6.5; it is recomended that you reinstall 'SeuratObject' as
#> the ABI for 'Matrix' may have changed
#> 
#> Attaching package: 'SeuratObject'
#> The following object is masked from 'package:BiocGenerics':
#> 
#>     intersect
#> The following object is masked from 'package:base':
#> 
#>     intersect
#> Loading required package: Seurat
#> 
#> Attaching package: 'Seurat'
#> The following object is masked from 'package:igraph':
#> 
#>     components
Seurat::SpatialDimPlot(visium.brain, label = T, label.size = 3, cols = color.use)
#> Scale for fill is already present.
#> Adding another scale for fill, which will replace the existing scale.
```

```
# Prepare input data for CelChat analysis
data.input = Seurat::GetAssayData(visium.brain, slot = "data", assay = "SCT") # normalized data matrix
#> Warning: The `slot` argument of `GetAssayData()` is deprecated as of SeuratObject 5.0.0.
#> ℹ Please use the `layer` argument instead.
#> This warning is displayed once every 8 hours.
#> Call `lifecycle::last_lifecycle_warnings()` to see where this warning was
#> generated.

# define the meta data: 
# a column named `samples` should be provided for spatial transcriptomics analysis, which is useful for analyzing cell-cell communication by aggregating multiple samples/replicates. Of note, for comparison analysis across different conditions, users still need to create a CellChat object seperately for each condition.  
meta = data.frame(labels = Seurat::Idents(visium.brain), samples = "sample1", row.names = names(Seurat::Idents(visium.brain))) # manually create a dataframe consisting of the cell labels
meta$samples <- factor(meta$samples)
unique(meta$labels) # check the cell labels
#> [1] L2/3 IT Astro   L6b     L5 IT   L6 IT   L6 CT   L4      Oligo  
#> Levels: Astro L2/3 IT L4 L5 IT L6 IT L6 CT L6b Oligo
unique(meta$samples) # check the sample labels
#> [1] sample1
#> Levels: sample1

# load spatial transcriptomics information
# Spatial locations of spots from full (NOT high/low) resolution images are required. For 10X Visium, this information is in `tissue_positions.csv`. 
spatial.locs = Seurat::GetTissueCoordinates(visium.brain, scale = NULL, cols = c("imagerow", "imagecol")) 

# Spatial factors of spatial coordinates
# For 10X Visium, the conversion factor of converting spatial coordinates from Pixels to Micrometers can be computed as the ratio of the theoretical spot size (i.e., 65um) over the number of pixels that span the diameter of a theoretical spot size in the full-resolution image (i.e., 'spot_diameter_fullres' in pixels in the 'scalefactors_json.json' file). 
# Of note, the 'spot_diameter_fullres' factor is different from the `spot` in Seurat object and thus users still need to get the value from the original json file. 
scalefactors = jsonlite::fromJSON(txt = file.path("/Users/suoqinjin/Library/CloudStorage/OneDrive-Personal/works/CellChat/tutorial/spatial_imaging_data_visium-brain", 'scalefactors_json.json'))
spot.size = 65 # the theoretical spot size (um) in 10X Visium
conversion.factor = spot.size/scalefactors$spot_diameter_fullres
spatial.factors = data.frame(ratio = conversion.factor, tol = spot.size/2)

d.spatial <- computeCellDistance(coordinates = spatial.locs, ratio = spatial.factors$ratio, tol = spatial.factors$tol)
min(d.spatial[d.spatial!=0]) # this value should approximately equal 100um for 10X Visium data
#> [1] 99.52835
```

## Create a CellChat object

**USERS can create a new CellChat object from a data matrix or
Seurat.** If input is a Seurat object, the meta data in the
object will be used by default and USER must provide
`group.by` to define the cell groups. e.g, group.by = “ident”
for the default cell identities in Seurat object.

**NB: If USERS load previously calculated CellChat object
(version < 2.1.0), please update the object via
`updateCellChat`**

```
cellchat <- createCellChat(object = data.input, meta = meta, group.by = "labels",
                           datatype = "spatial", coordinates = spatial.locs, spatial.factors = spatial.factors)
#> [1] "Create a CellChat object from a data matrix"
#> Create a CellChat object from spatial transcriptomics data... 
#> Set cell identities for the new CellChat object 
#> The cell groups used for CellChat analysis are  Astro, L2/3 IT, L4, L5 IT, L6 IT, L6 CT, L6b, Oligo
cellchat
#> An object of class CellChat created from a single dataset 
#>  16019 genes.
#>  1073 cells. 
#> CellChat analysis of spatial data! The input spatial locations are 
#>                    x_cent y_cent
#> AAACAGAGCGACTCCT-1   3164   7950
#> AAACCGGGTAGGTACC-1   6517   3407
#> AAACCGTTCGTCCAGG-1   7715   4371
#> AAACTCGTGATATAAG-1   4242   9258
#> AAAGGGATGTAGCAAG-1   4362   5747
#> AAATAACCATACGGGA-1   3164   7537
```

## Set the ligand-receptor interaction database

Before users can employ CellChat to infer cell-cell communication,
they need to set the ligand-receptor interaction database and identify
over-expressed ligands or receptors.

Our database CellChatDB is a manually curated database of
literature-supported ligand-receptor interactions in both human and
mouse. CellChatDB v2 contains ~3,300 validated molecular interactions,
including ~40% of secrete autocrine/paracrine signaling interactions,
~17% of extracellular matrix (ECM)-receptor interactions, ~13% of
cell-cell contact interactions and ~30% non-protein signaling. Compared
to CellChatDB v1, CellChatDB v2 adds more than 1000 protein and
non-protein interactions such as metabolic and synaptic signaling. It
should be noted that for molecules that are not directly related to
genes measured in scRNA-seq, CellChat v2 estimates the expression of
ligands and receptors using those molecules’ key mediators or enzymes
for potential communication mediated by non-proteins.

CellChatDB v2 also adds additional functional annotations of
ligand-receptor pairs, such as UniProtKB keywords (including biological
process, molecular function, functional class, disease, etc),
subcellular location and relevance to neurotransmitter.

Users can update CellChatDB by adding their own curated
ligand-receptor pairs. Please check the tutorial
on updating the ligand-receptor interaction database CellChatDB.

When analyzing human samples, use the database
**`CellChatDB.human`**; when analyzing mouse
samples, use the database
**`CellChatDB.mouse`**. CellChatDB categorizes
ligand-receptor pairs into different types, including “Secreted
Signaling”, “ECM-Receptor”, “Cell-Cell Contact” and “Non-protein
Signaling”. By default, the “Non-protein Signaling” are not used.

```
CellChatDB <- CellChatDB.mouse # use CellChatDB.human if running on human data
showDatabaseCategory(CellChatDB)
```

```
# Show the structure of the database
dplyr::glimpse(CellChatDB$interaction)
#> Rows: 3,379
#> Columns: 28
#> $ interaction_name         <chr> "TGFB1_TGFBR1_TGFBR2", "TGFB2_TGFBR1_TGFBR2",…
#> $ pathway_name             <chr> "TGFb", "TGFb", "TGFb", "TGFb", "TGFb", "TGFb…
#> $ ligand                   <chr> "Tgfb1", "Tgfb2", "Tgfb3", "Tgfb1", "Tgfb1", …
#> $ receptor                 <chr> "TGFbR1_R2", "TGFbR1_R2", "TGFbR1_R2", "ACVR1…
#> $ agonist                  <chr> "TGFb agonist", "TGFb agonist", "TGFb agonist…
#> $ antagonist               <chr> "TGFb antagonist", "TGFb antagonist", "TGFb a…
#> $ co_A_receptor            <chr> "", "", "", "", "", "", "", "", "", "", "", "…
#> $ co_I_receptor            <chr> "TGFb inhibition receptor", "TGFb inhibition …
#> $ evidence                 <chr> "KEGG: mmu04350", "KEGG: mmu04350", "KEGG: mm…
#> $ annotation               <chr> "Secreted Signaling", "Secreted Signaling", "…
#> $ interaction_name_2       <chr> "Tgfb1 - (Tgfbr1+Tgfbr2)", "Tgfb2  - (Tgfbr1+…
#> $ is_neurotransmitter      <lgl> FALSE, FALSE, FALSE, FALSE, FALSE, FALSE, FAL…
#> $ ligand.symbol            <chr> "Tgfb1", "Tgfb2", "Tgfb3", "Tgfb1", "Tgfb1", …
#> $ ligand.family            <chr> "TGF-beta", "TGF-beta", "TGF-beta", "TGF-beta…
#> $ ligand.location          <chr> "Extracellular matrix, Secreted, Extracellula…
#> $ ligand.keyword           <chr> "Disease variant, Signal, Reference proteome,…
#> $ ligand.secreted_type     <chr> "growth factor", "growth factor", "cytokine;g…
#> $ ligand.transmembrane     <lgl> FALSE, FALSE, TRUE, FALSE, FALSE, FALSE, FALS…
#> $ receptor.symbol          <chr> "Tgfbr1, Tgfbr2", "Tgfbr1, Tgfbr2", "Tgfbr1, …
#> $ receptor.family          <chr> "Protein kinase superfamily, TKL Ser/Thr prot…
#> $ receptor.location        <chr> "Cell membrane, Secreted, Membrane raft, Cell…
#> $ receptor.keyword         <chr> "Membrane, Secreted, Disulfide bond, Kinase, …
#> $ receptor.surfaceome_main <chr> "Receptors", "Receptors", "Receptors", "Recep…
#> $ receptor.surfaceome_sub  <chr> "Act.TGFB;Kinase", "Act.TGFB;Kinase", "Act.TG…
#> $ receptor.adhesome        <chr> "", "", "", "", "", "", "", "", "", "", "", "…
#> $ receptor.secreted_type   <chr> "", "", "", "", "", "", "", "", "", "", "", "…
#> $ receptor.transmembrane   <lgl> TRUE, TRUE, TRUE, TRUE, TRUE, TRUE, TRUE, TRU…
#> $ version                  <chr> "CellChatDB v1", "CellChatDB v1", "CellChatDB…

# use a subset of CellChatDB for cell-cell communication analysis
CellChatDB.use <- subsetDB(CellChatDB, search = "Secreted Signaling", key = "annotation") # use Secreted Signaling

# Only uses the Secreted Signaling from CellChatDB v1
#  CellChatDB.use <- subsetDB(CellChatDB, search = list(c("Secreted Signaling"), c("CellChatDB v1")), key = c("annotation", "version"))

# use all CellChatDB except for "Non-protein Signaling" for cell-cell communication analysis
# CellChatDB.use <- subsetDB(CellChatDB)


# use all CellChatDB for cell-cell communication analysis
# CellChatDB.use <- CellChatDB # simply use the default CellChatDB. We do not suggest to use it in this way because CellChatDB v2 includes "Non-protein Signaling" (i.e., metabolic and synaptic signaling) that can be only estimated from gene expression data. 

# set the used database in the object
cellchat@DB <- CellChatDB.use
```

## Preprocessing the expression data for cell-cell communication analysis

To infer the cell state-specific communications, CellChat identifies
over-expressed ligands or receptors in one cell group and then
identifies over-expressed ligand-receptor interactions if either ligand
or receptor are over-expressed.

We also provide a function to project gene expression data onto
protein-protein interaction (PPI) network. Specifically, a diffusion
process is used to smooth genes’ expression values based on their
neighbors’ defined in a high-confidence experimentally validated
protein-protein network. This function is useful when analyzing
single-cell data with shallow sequencing depth because the projection
reduces the dropout effects of signaling genes, in particular for
possible zero expression of subunits of ligands/receptors. One might be
concerned about the possible artifact introduced by this diffusion
process, however, it will only introduce very weak communications. By
default CellChat uses the raw data (i.e.,
`object@data.signaling`) instead of the projected data. To
use the projected data, users should run the function
`projectData` before running `computeCommunProb`,
and then set `raw.use = FALSE` when running
`computeCommunProb`.

```
# subset the expression data of signaling genes for saving computation cost
cellchat <- subsetData(cellchat) # This step is necessary even if using the whole database
future::plan("multisession", workers = 4) 
cellchat <- identifyOverExpressedGenes(cellchat)
cellchat <- identifyOverExpressedInteractions(cellchat, variable.both = F)
#> The number of highly variable ligand-receptor pairs used for signaling inference is 648
 
# project gene expression data onto PPI (Optional: when running it, USER should set `raw.use = FALSE` in the function `computeCommunProb()` in order to use the projected data)
# cellchat <- projectData(cellchat, PPI.mouse)
execution.time = Sys.time() - ptm
print(as.numeric(execution.time, units = "secs"))
#> [1] 18.24813
```

# Part II: Inference of cell-cell communication network

CellChat infers the biologically significant cell-cell communication
by assigning each interaction with a probability value and peforming a
permutation test. CellChat models the probability of cell-cell
communication by integrating gene expression with prior known knowledge
of the interactions between signaling ligands, receptors and their
cofactors using the law of mass action.

CAUTION: The number of inferred ligand-receptor pairs clearly depends
on the **method for calculating the average gene expression per
cell group**. By default, CellChat uses a statistically robust
mean method called ‘trimean’, which produces fewer interactions than
other methods. However, we find that CellChat performs well at
predicting stronger interactions, which is very helpful for narrowing
down on interactions for further experimental validations. In
`computeCommunProb`, we provide an option for using other
methods, such as 5% and 10% truncated mean, to calculating the average
gene expression. Of note, ‘trimean’ approximates 25% truncated mean,
implying that the average gene expression is zero if the percent of
expressed cells in one group is less than 25%. To use 10% truncated
mean, USER can set `type = "truncatedMean"` and
`trim = 0.1`. To determine a proper value of trim, CellChat
provides a function `computeAveExpr`, which can help to check
the average expression of signaling genes of interest, e.g,
`computeAveExpr(cellchat, features = c("CXCL12","CXCR4"), type = "truncatedMean", trim = 0.1)`.
Therefore, if well-known signaling pathways in the studied biological
process are not predicted, users can try `truncatedMean` with
lower values of `trim` to change the method for calculating
the average gene expression per cell group.

## Compute the communication probability and infer cellular communication network

To quickly examine the inference results, USER can set
`nboot = 20` in `computeCommunProb`. Then “pvalue
< 0.05” means none of the permutation results are larger than the
observed communication probability.

If well-known signaling pathways in the studied biological process
are not predicted, USER can try `truncatedMean` with lower
values of `trim` to change the method for calculating the
average gene expression per cell group.

USERS may need to adjust the parameter `scale.distance`
when working on data from other spatial transcriptomics technologies.
Please check the documentation in detail via
`?computeCommunProb`.

When inferring contact-dependent or juxtacrine signaling, users
should provide a value of `contact.range` and set
`contact.dependent = TRUE`. Briefly, users can set
`contact.range = 10`, which is a typical human cell size.
However, for low-resolution spatial data such as 10X visium, it should
be the cell center-to-center distance (i.e.,
`contact.range = 100` for 10X visium data). Please check the
vignette
of FAQ on applying CellChat to spatially resolved transcriptomics
data for detailed explanations. In this example, we did not use the
L-R pairs from `Cell-Cell Contact` signaling, therefore we
can set `contact.dependent = FALSE` and
`contact.range = NULL`. But as an illustration, we use the
following settings that lead to the same results.

```
ptm = Sys.time()

cellchat <- computeCommunProb(cellchat, type = "truncatedMean", trim = 0.1,
                              distance.use = TRUE, interaction.range = 250, scale.distance = 0.01,
                              contact.dependent = TRUE, contact.range = 100)
#> truncatedMean is used for calculating the average gene expression per cell group. 
#> [1] ">>> Run CellChat on spatial transcriptomics data using distances as constraints of the computed communication probability <<< [2024-02-22 10:39:52.098053]"
#> Molecules of the input L-R pairs are diffusible. Run CellChat in a diffusion manner based on the `interaction.range`.
#> [1] ">>> CellChat inference is done. Parameter values are stored in `object@options$parameter` <<< [2024-02-22 10:54:30.589965]"
```

Users can filter out the cell-cell communication if there are only
few cells in certain cell groups. By default, the minimum number of
cells required in each cell group for cell-cell communication is 10.

```
cellchat <- filterCommunication(cellchat, min.cells = 10)
```

## Extract the inferred cellular communication network as a data frame

We provide a function `subsetCommunication` to easily
access the inferred cell-cell communications of interest. For
example,

- `df.net <- subsetCommunication(cellchat)` returns a
  data frame consisting of all the inferred cell-cell communications at
  the level of ligands/receptors. Set `slot.name = "netP"` to
  access the the inferred communications at the level of signaling
  pathways
- `df.net <- subsetCommunication(cellchat, sources.use = c(1,2), targets.use = c(4,5))`
  gives the inferred cell-cell communications sending from cell groups 1
  and 2 to cell groups 4 and 5.
- `df.net <- subsetCommunication(cellchat, signaling = c("WNT", "TGFb"))`
  gives the inferred cell-cell communications mediated by signaling WNT
  and TGFb.

## Infer the cell-cell communication at a signaling pathway level

CellChat computes the communication probability on signaling pathway
level by summarizing the communication probabilities of all
ligands-receptors interactions associated with each signaling
pathway.

NB: The inferred intercellular communication network of each
ligand-receptor pair and each signaling pathway is stored in the slot
‘net’ and ‘netP’, respectively.

```
cellchat <- computeCommunProbPathway(cellchat)
```

## Calculate the aggregated cell-cell communication network

We can calculate the aggregated cell-cell communication network by
counting the number of links or summarizing the communication
probability. USER can also calculate the aggregated network among a
subset of cell groups by setting `sources.use` and
`targets.use`.

```
cellchat <- aggregateNet(cellchat)

execution.time = Sys.time() - ptm
print(as.numeric(execution.time, units = "secs"))
#> [1] 879.9437
```

We can also visualize the aggregated cell-cell communication network.
For example, showing the number of interactions or the total interaction
strength (weights) between any two cell groups using circle plot or
heatmap plot.

```
ptm = Sys.time()

groupSize <- as.numeric(table(cellchat@idents))
par(mfrow = c(1,2), xpd=TRUE)
netVisual_circle(cellchat@net$count, vertex.weight = rowSums(cellchat@net$count), weight.scale = T, label.edge= F, title.name = "Number of interactions")
netVisual_circle(cellchat@net$weight, vertex.weight = rowSums(cellchat@net$weight), weight.scale = T, label.edge= F, title.name = "Interaction weights/strength")
```

```
netVisual_heatmap(cellchat, measure = "count", color.heatmap = "Blues")
#> Do heatmap based on a single object
```

```
netVisual_heatmap(cellchat, measure = "weight", color.heatmap = "Blues")
#> Do heatmap based on a single object
```

# Part III: Visualization of cell-cell communication network

Upon infering the cell-cell communication network, CellChat provides
various functionality for further data exploration, analysis, and
visualization. Here we only showcase the `circle plot` and
the new `spatial plot`.

**Visualization of cell-cell communication at different
levels**: One can visualize the inferred communication network of
signaling pathways using `netVisual_aggregate`, and visualize
the inferred communication networks of individual L-R pairs associated
with that signaling pathway using `netVisual_individual`.

Here we take input of one signaling pathway as an example. All the
signaling pathways showing significant communications can be accessed by
`cellchat@netP$pathways`.

```
pathways.show <- c("IGF") 
# Circle plot
par(mfrow=c(1,1), xpd = TRUE) # `xpd = TRUE` should be added to show the title
netVisual_aggregate(cellchat, signaling = pathways.show, layout = "circle")
```

```
# Spatial plot
par(mfrow=c(1,1))
netVisual_aggregate(cellchat, signaling = pathways.show, layout = "spatial", edge.width.max = 2, vertex.size.max = 1, alpha.image = 0.2, vertex.label.cex = 3.5)
```

```
execution.time = Sys.time() - ptm
print(as.numeric(execution.time, units = "secs"))
#> [1] 1.59349
```

Compute and visualize the network centrality scores:

```
# Compute the network centrality scores
cellchat <- netAnalysis_computeCentrality(cellchat, slot.name = "netP") # the slot 'netP' means the inferred intercellular communication network of signaling pathways
# Visualize the computed centrality scores using heatmap, allowing ready identification of major signaling roles of cell groups
par(mfrow=c(1,1))
netAnalysis_signalingRole_network(cellchat, signaling = pathways.show, width = 8, height = 2.5, font.size = 10)
```

```
# USER can show this information on the spatial transcriptomics when visualizing a signaling network, e.g., bigger circle indicates larger incoming signaling
par(mfrow=c(1,1))
netVisual_aggregate(cellchat, signaling = pathways.show, layout = "spatial", edge.width.max = 2, alpha.image = 0.2, vertex.weight = "incoming", vertex.size.max = 4, vertex.label.cex = 3.5)
```

Visualize gene expression distribution on tissue

```
# Take an input of a few genes
spatialFeaturePlot(cellchat, features = c("Igf1","Igf1r"), point.size = 0.8, color.heatmap = "Reds", direction = 1)
```

```
# Take an input of a ligand-receptor pair
spatialFeaturePlot(cellchat, pairLR.use = "IGF1_IGF1R", point.size = 0.5, do.binary = FALSE, cutoff = 0.05, enriched.only = F, color.heatmap = "Reds", direction = 1)
#> Applying a cutoff of  0.05 to the values...
```

```
# Take an input of a ligand-receptor pair and show expression in binary
spatialFeaturePlot(cellchat, pairLR.use = "IGF1_IGF1R", point.size = 1, do.binary = TRUE, cutoff = 0.05, enriched.only = F, color.heatmap = "Reds", direction = 1)
```

**NB: Upon infering the intercellular communication network
from spatial transcriptomics data, CellChat’s various functionality can
be used for further data exploration, analysis, and visualization.
Please check other functionalities in the basic
tutorial of CellChat**

# Part V: Save the CellChat object

```
saveRDS(cellchat, file = "cellchat_visium_mouse_cortex.rds")
```

# Part VI: Explore the cell-cell communication through the Interactive CellChat Explorer

```
runCellChatApp(cellchat)
```

# Part VII: Application to different technologies of spatially-resolved transcriptomics

Please check the vignette
of FAQ on applying CellChat to spatially resolved transcriptomics
data for detailed setting of different technologies of spatial
transcriptomics data.

```
sessionInfo()
#> R version 4.3.1 (2023-06-16)
#> Platform: aarch64-apple-darwin20 (64-bit)
#> Running under: macOS Ventura 13.5
#> 
#> Matrix products: default
#> BLAS:   /Library/Frameworks/R.framework/Versions/4.3-arm64/Resources/lib/libRblas.0.dylib 
#> LAPACK: /Library/Frameworks/R.framework/Versions/4.3-arm64/Resources/lib/libRlapack.dylib;  LAPACK version 3.11.0
#> 
#> locale:
#> [1] en_US.UTF-8/en_US.UTF-8/en_US.UTF-8/C/en_US.UTF-8/en_US.UTF-8
#> 
#> time zone: Asia/Shanghai
#> tzcode source: internal
#> 
#> attached base packages:
#> [1] stats     graphics  grDevices utils     datasets  methods   base     
#> 
#> other attached packages:
#>  [1] Seurat_5.0.1        SeuratObject_5.0.1  sp_2.1-0           
#>  [4] patchwork_1.1.3     CellChat_2.1.2      Biobase_2.60.0     
#>  [7] BiocGenerics_0.46.0 ggplot2_3.4.3       igraph_1.5.1       
#> [10] dplyr_1.1.3        
#> 
#> loaded via a namespace (and not attached):
#>   [1] RcppAnnoy_0.0.21       splines_4.3.1          later_1.3.1           
#>   [4] tibble_3.2.1           polyclip_1.10-4        ggnetwork_0.5.12      
#>   [7] fastDummies_1.7.3      lifecycle_1.0.3        rstatix_0.7.2.999     
#>  [10] doParallel_1.0.17      globals_0.16.2         lattice_0.21-8        
#>  [13] MASS_7.3-60            backports_1.4.1        magrittr_2.0.3        
#>  [16] plotly_4.10.2          sass_0.4.7             rmarkdown_2.24        
#>  [19] jquerylib_0.1.4        yaml_2.3.7             httpuv_1.6.11         
#>  [22] NMF_0.26               sctransform_0.4.1      spam_2.9-1            
#>  [25] spatstat.sparse_3.0-2  reticulate_1.31        cowplot_1.1.1         
#>  [28] pbapply_1.7-2          RColorBrewer_1.1-3     abind_1.4-5           
#>  [31] Rtsne_0.16             purrr_1.0.2            presto_1.0.0          
#>  [34] circlize_0.4.16        IRanges_2.34.1         S4Vectors_0.38.1      
#>  [37] ggrepel_0.9.3          irlba_2.3.5.1          listenv_0.9.0         
#>  [40] spatstat.utils_3.0-3   goftest_1.2-3          RSpectra_0.16-1       
#>  [43] spatstat.random_3.1-5  fitdistrplus_1.1-11    parallelly_1.36.0     
#>  [46] svglite_2.1.1          leiden_0.4.3           codetools_0.2-19      
#>  [49] tidyselect_1.2.0       shape_1.4.6            farver_2.1.1          
#>  [52] matrixStats_1.0.0      stats4_4.3.1           spatstat.explore_3.2-1
#>  [55] jsonlite_1.8.7         GetoptLong_1.0.5       BiocNeighbors_1.18.0  
#>  [58] ellipsis_0.3.2         progressr_0.14.0       ggridges_0.5.4        
#>  [61] ggalluvial_0.12.5      survival_3.5-7         iterators_1.0.14      
#>  [64] systemfonts_1.0.4      foreach_1.5.2          tools_4.3.1           
#>  [67] sna_2.7-1              ica_1.0-3              Rcpp_1.0.11           
#>  [70] glue_1.6.2             gridExtra_2.3          xfun_0.40             
#>  [73] withr_2.5.0            BiocManager_1.30.22    fastmap_1.1.1         
#>  [76] fansi_1.0.4            digest_0.6.33          R6_2.5.1              
#>  [79] mime_0.12              colorspace_2.1-0       scattermore_1.2       
#>  [82] tensor_1.5             spatstat.data_3.0-1    utf8_1.2.3            
#>  [85] tidyr_1.3.0            generics_0.1.3         data.table_1.14.9     
#>  [88] FNN_1.1.3.2            httr_1.4.7             htmlwidgets_1.6.2     
#>  [91] uwot_0.1.16            pkgconfig_2.0.3        gtable_0.3.4          
#>  [94] registry_0.5-1         ComplexHeatmap_2.15.4  lmtest_0.9-40         
#>  [97] htmltools_0.5.6        carData_3.0-5          dotCall64_1.0-2       
#> [100] clue_0.3-64            scales_1.2.1           tidyverse_2.0.0       
#> [103] png_0.1-8              knitr_1.43             rstudioapi_0.15.0     
#> [106] reshape2_1.4.4         rjson_0.2.21           nlme_3.1-163          
#> [109] coda_0.19-4            statnet.common_4.9.0   cachem_1.0.8          
#> [112] zoo_1.8-12             GlobalOptions_0.1.2    stringr_1.5.0         
#> [115] KernSmooth_2.23-22     parallel_4.3.1         miniUI_0.1.1.1        
#> [118] pillar_1.9.0           grid_4.3.1             vctrs_0.6.3           
#> [121] RANN_2.6.1             promises_1.2.1         ggpubr_0.6.0          
#> [124] car_3.1-2              xtable_1.8-4           cluster_2.1.4         
#> [127] evaluate_0.21          magick_2.8.1           cli_3.6.1             
#> [130] compiler_4.3.1         rlang_1.1.1            crayon_1.5.2          
#> [133] rngtools_1.5.2         future.apply_1.11.0    ggsignif_0.6.4        
#> [136] labeling_0.4.3         plyr_1.8.8             stringi_1.7.12        
#> [139] deldir_1.0-9           viridisLite_0.4.2      network_1.18.1        
#> [142] gridBase_0.4-7         BiocParallel_1.34.2    munsell_0.5.0         
#> [145] lazyeval_0.2.2         spatstat.geom_3.2-4    Matrix_1.6-5          
#> [148] RcppHNSW_0.5.0         future_1.33.0          shiny_1.7.5           
#> [151] highr_0.10             ROCR_1.0-11            broom_1.0.5           
#> [154] bslib_0.5.1
```
