# Supplementary material for: CSNK2B Expression Aligns with a GDF15-Associated Immune Molecular State in Hepatocellular Carcinoma
Source: Biomedicines. 2026 Aug 26;14(9):1909. doi: 10.3390/biomedicines14091909 (PMC13604900; doi:10.3390/biomedicines14091909)
Supplement: Supplementary file 1 [file biomedicines-14-01909-s001.zip › Supplemental file S1/docker_cellchat/CellChat-main-http1.tar.gz!/CellChat-main/tutorial/Comparison_analysis_of_multiple_datasets.html]

Comparison analysis of multiple datasets using CellChat


# Comparison analysis of multiple datasets using CellChat

#### Suoqin Jin

#### 14 February, 2024

- Load the required
  libraries
- Create a directory to save
  figures
- Load
  CellChat object of each dataset and merge them together
- Part
  I: Identify altered interactions and cell populations
  - Compare
    the total number of interactions and interaction strength
  - Compare
    the number of interactions and interaction strength among different cell
    populations
    - (A)
      Circle plot showing differential number of interactions or interaction
      strength among different cell populations across two datasets
    - (B)
      Heatmap showing differential number of interactions or interaction
      strength among different cell populations across two datasets
    - (C)
      Circle plot showing the number of interactions or interaction strength
      among different cell populations across multiple datasets
    - (D)
      Circle plot showing the differential number of interactions or
      interaction strength among coarse cell types
  - Compare the
    major sources and targets in a 2D space
    - (A)
      Identify cell populations with significant changes in sending or
      receiving signals
    - (B)
      Identify the signaling changes of specific cell populations
- Part
  II: Identify altered signaling with distinct network architecture and
  interaction strength
  - Identify
    signaling networks with larger (or less) difference as well as signaling
    groups based on their functional/structure similarity
    - Identify
      signaling groups based on their functional similarity
    - Identify
      signaling groups based on structure similarity
    - Compute
      and visualize the pathway distance in the learned joint
      manifold
  - Identify
    altered signaling with distinct interaction strength
    - (A)
      Compare the overall information flow of each signaling pathway or
      ligand-receptor pair
    - (B)
      Compare outgoing (or incoming) signaling patterns associated with each
      cell population
- Part
  III: Identify the up-gulated and down-regulated signaling
  ligand-receptor pairs
  - Identify
    dysfunctional signaling by comparing the communication
    probabities
  - Identify
    dysfunctional signaling by using differential expression
    analysis
  - Visualize
    the identified up-regulated and down-regulated signaling ligand-receptor
    pairs
    - (A) Bubble
      plot
    - (B) Chord
      diagram
    - (C) Wordcloud
      plot
- Part
  IV: Visually compare cell-cell communication using Hierarchy plot,
  Circle plot or Chord diagram
- Part
  V: Compare the signaling gene expression distribution between different
  datasets
- Save the merged CellChat
  object

This vignette shows how to apply CellChat to identify major signaling
changes across different biological conditions by quantitative contrasts
and joint manifold learning. We showcase CellChat’s diverse
functionalities for identifying major signaling changes across
conditions by applying it to scRNA-seq datasets from two biological
conditions: nonlesional (NL, normal) and lesional (LS, diseased) human
skin from patients with atopic dermatitis. **These two datasets
(conditions) have the same cell population compositions after joint
clustering. If there are different cell population compositions between
different conditions, please check out the tutorial
on Comparison analysis of multiple datasets with different cell type
compositions**.

CellChat employs a top-down approach, i.e., starting with the big
picture and then refining it in a greater detail on the signaling
mechanisms, to identify signaling changes at different levels, including
altered interactions, cell populations, signaling pathways and
ligand-receptor pairs.

## Load the required libraries

```
ptm = Sys.time()
library(CellChat)
library(patchwork)
# reticulate::use_python("/Users/suoqinjin/anaconda3/bin/python", required=T)
```

## Create a directory to save figures

```
data.dir <- './comparison'
dir.create(data.dir)
setwd(data.dir)
```

# Load CellChat object of each dataset and merge them together

Users need to first run CellChat on each dataset separately and then
merge different CellChat objects together. Please do
`updateCellChat` if the CellChat objects are obtained using
the earlier version (< 1.6.0).

```
cellchat.NL <- readRDS("/Users/suoqinjin/Library/CloudStorage/OneDrive-Personal/works/CellChat/tutorial/cellchat_humanSkin_NL.rds")
cellchat.LS <- readRDS("/Users/suoqinjin/Library/CloudStorage/OneDrive-Personal/works/CellChat/tutorial/cellchat_humanSkin_LS.rds")
object.list <- list(NL = cellchat.NL, LS = cellchat.LS)
cellchat <- mergeCellChat(object.list, add.names = names(object.list))
#> Merge the following slots: 'data.signaling','images','net', 'netP','meta', 'idents', 'var.features' , 'DB', and 'LR'.
cellchat
#> An object of class CellChat created from a merged object with multiple datasets 
#>  593 signaling genes.
#>  7563 cells. 
#> CellChat analysis of single cell RNA-seq data!
execution.time = Sys.time() - ptm
print(as.numeric(execution.time, units = "secs"))
#> [1] 2.763455
```

```
# Users can now export the merged CellChat object and the list of the two separate objects for later use
save(object.list, file = "cellchat_object.list_humanSkin_NL_LS.RData")
save(cellchat, file = "cellchat_merged_humanSkin_NL_LS.RData")
```

# Part I: Identify altered interactions and cell populations

CellChat employs a top-down approach, i.e., starting with the big
picture and then refining it in a greater detail on the signaling
mechanisms, to identify signaling changes at different levels, including
altered interactions, cell populations, signaling pathways and
ligand-receptor pairs. First, CellChat starts with a big picture to
answer the following questions:

- Whether the cell-cell communication is enhanced or not
- The interaction between which cell types is significantly
  changed
- How the major sources and targets change from one condition to
  another

## Compare the total number of interactions and interaction strength

To answer the question on whether the cell-cell communication is
enhanced or not, CellChat compares the total number of interactions and
interaction strength of the inferred cell-cell communication networks
from different biological conditions.

```
ptm = Sys.time()
gg1 <- compareInteractions(cellchat, show.legend = F, group = c(1,2))
gg2 <- compareInteractions(cellchat, show.legend = F, group = c(1,2), measure = "weight")
gg1 + gg2
```

## Compare the number of interactions and interaction strength among different cell populations

To identify the interaction between which cell populations showing
significant changes, CellChat compares the number of interactions and
interaction strength among different cell populations using a circle
plot with differential interactions (option A), a heatmap with
differential interactions (option B) and two circle plots with the
number of interactions or interaction strength per dataset (option C).
Alternatively, users can examine the differential number of interactions
or interaction strength among coarse cell types by aggregating the
cell-cell communication based on the defined cell groups (option D).

### (A) Circle plot showing differential number of interactions or interaction strength among different cell populations across two datasets

The differential number of interactions or interaction strength in
the cell-cell communication network between two datasets can be
visualized using circle plot, where \(\color{red}{\text{red}}\) (or \(\color{blue}{\text{blue}}\)) colored edges
represent \(\color{red}{\text{increased}}\) (or \(\color{blue}{\text{decreased}}\)) signaling
in the second dataset compared to the first one.

```
par(mfrow = c(1,2), xpd=TRUE)
netVisual_diffInteraction(cellchat, weight.scale = T)
netVisual_diffInteraction(cellchat, weight.scale = T, measure = "weight")
```

### (B) Heatmap showing differential number of interactions or interaction strength among different cell populations across two datasets

CellChat can also show differential number of interactions or
interaction strength in greater details using a heatmap. The top colored
bar plot represents the sum of each column of the absolute values
displayed in the heatmap (incoming signaling). The right colored bar
plot represents the sum of each row of the absolute values (outgoing
signaling). Therefore, the bar height indicates the degree of change in
terms of the number of interactions or interaction strength between the
two conditions. In the colorbar, \(\color{red}{\text{red}}\) (or \(\color{blue}{\text{blue}}\)) represents
\(\color{red}{\text{increased}}\) (or
\(\color{blue}{\text{decreased}}\))
signaling in the second dataset compared to the first one.

```
gg1 <- netVisual_heatmap(cellchat)
#> Do heatmap based on a merged object
gg2 <- netVisual_heatmap(cellchat, measure = "weight")
#> Do heatmap based on a merged object
gg1 + gg2
```

### (C) Circle plot showing the number of interactions or interaction strength among different cell populations across multiple datasets

The above differential network analysis only works for pairwise
datasets. If there are more datasets for comparison, CellChat can
directly show the number of interactions or interaction strength between
any two cell populations in each dataset.

To better control the node size and edge weights of the inferred
networks across different datasets, CellChat computes the maximum number
of cells per cell group and the maximum number of interactions (or
interaction weights) across all datasets.

```
weight.max <- getMaxWeight(object.list, attribute = c("idents","count"))
par(mfrow = c(1,2), xpd=TRUE)
for (i in 1:length(object.list)) {
  netVisual_circle(object.list[[i]]@net$count, weight.scale = T, label.edge= F, edge.weight.max = weight.max[2], edge.width.max = 12, title.name = paste0("Number of interactions - ", names(object.list)[i]))
}
```

### (D) Circle plot showing the differential number of interactions or interaction strength among coarse cell types

To simplify the complicated network and gain insights into the
cell-cell communication at the cell type level, CellChat aggregates the
cell-cell communication based on the defined cell groups. Here we
categorize the cell populations into three cell types, and then re-merge
the list of CellChat object.

```
group.cellType <- c(rep("FIB", 4), rep("DC", 4), rep("TC", 4))
group.cellType <- factor(group.cellType, levels = c("FIB", "DC", "TC"))
object.list <- lapply(object.list, function(x) {mergeInteractions(x, group.cellType)})
cellchat <- mergeCellChat(object.list, add.names = names(object.list))
#> Merge the following slots: 'data.signaling','images','net', 'netP','meta', 'idents', 'var.features' , 'DB', and 'LR'.
```

We then can show the number of interactions or interaction strength
between any two cell types in each dataset.

```
weight.max <- getMaxWeight(object.list, slot.name = c("idents", "net", "net"), attribute = c("idents","count", "count.merged"))
par(mfrow = c(1,2), xpd=TRUE)
for (i in 1:length(object.list)) {
  netVisual_circle(object.list[[i]]@net$count.merged, weight.scale = T, label.edge= T, edge.weight.max = weight.max[3], edge.width.max = 12, title.name = paste0("Number of interactions - ", names(object.list)[i]))
}
```

Similarly, CellChat can also show the differential number of
interactions or interaction strength between any two cell types using
circle plot. Red (or blue) colored edges represent increased (or
decreased) signaling in the second dataset compared to the first
one.

```
par(mfrow = c(1,2), xpd=TRUE)
netVisual_diffInteraction(cellchat, weight.scale = T, measure = "count.merged", label.edge = T)
netVisual_diffInteraction(cellchat, weight.scale = T, measure = "weight.merged", label.edge = T)
```

## Compare the major sources and targets in a 2D space

Comparing the outgoing and incoming interaction strength in a 2D
space allows ready identification of the cell populations with
significant changes in sending or receiving signals between different
datasets.

Identify cell populations with significant changes in sending or
receiving signals between different datasets by following option A, and
the signaling changes of specific cell populations by following option
B.

### (A) Identify cell populations with significant changes in sending or receiving signals

```
num.link <- sapply(object.list, function(x) {rowSums(x@net$count) + colSums(x@net$count)-diag(x@net$count)})
weight.MinMax <- c(min(num.link), max(num.link)) # control the dot size in the different datasets
gg <- list()
for (i in 1:length(object.list)) {
  gg[[i]] <- netAnalysis_signalingRole_scatter(object.list[[i]], title = names(object.list)[i], weight.MinMax = weight.MinMax)
}
#> Signaling role analysis on the aggregated cell-cell communication network from all signaling pathways
#> Signaling role analysis on the aggregated cell-cell communication network from all signaling pathways
patchwork::wrap_plots(plots = gg)
```

From the scatter plot, we can see that Inflam.DC and cDC1 emerge as one
of the major source and targets in LS compared to NL. Fibroblast
populations also become the major sources in LS.

### (B) Identify the signaling changes of specific cell populations

Furthermore, we can identify the specific signaling changes of
Inflam.DC and cDC1 between NL and LS.

```
gg1 <- netAnalysis_signalingChanges_scatter(cellchat, idents.use = "Inflam. DC", signaling.exclude = "MIF")
#> Visualizing differential outgoing and incoming signaling changes from NL to LS
#> The following `from` values were not present in `x`: 0
#> The following `from` values were not present in `x`: 0, -1
gg2 <- netAnalysis_signalingChanges_scatter(cellchat, idents.use = "cDC1", signaling.exclude = c("MIF"))
#> Visualizing differential outgoing and incoming signaling changes from NL to LS
#> The following `from` values were not present in `x`: 0, 2
#> The following `from` values were not present in `x`: 0, -1
patchwork::wrap_plots(plots = list(gg1,gg2))
```

```
execution.time = Sys.time() - ptm
print(as.numeric(execution.time, units = "secs"))
#> [1] 1.982786
```

# Part II: Identify altered signaling with distinct network architecture and interaction strength

CellChat then can identify signaling with larger (or smaller) network
difference as well as signaling groups based on their functional or
structure similarity across multiple biological conditions.

## Identify signaling networks with larger (or less) difference as well as signaling groups based on their functional/structure similarity

CellChat performs joint manifold learning and classification of the
inferred communication networks based on their functional and
topological similarity across different conditions. NB: Such analysis is
applicable to more than two datasets.

By quantifying the similarity between the cellular communication
networks of signaling pathways across conditions, this analysis
highlights the potentially altered signaling pathways. CellChat adopts
the concept of network rewiring from network biology and hypothesized
that the difference between different communication networks may affect
biological processes across conditions. UMAP is used for visualizing
signaling relationship and interpreting our signaling outputs in an
intuitive way without involving the classification of conditions.

**Functional similarity**: High degree of functional
similarity indicates major senders and receivers are similar, and it can
be interpreted as the two signaling pathways or two ligand-receptor
pairs exhibit similar and/or redundant roles. **NB**:
Functional similarity analysis is not applicable to multiple datsets
with different cell type composition.

**Structural similarity**: A structural similarity was
used to compare their signaling network structure, without considering
the similarity of senders and receivers. **NB**: Structural
similarity analysis is applicable to multiple datsets with the same cell
type composition or the vastly different cell type composition.

Here we can run the manifold and classification learning analysis
based on the functional similarity because the two datasets have the the
same cell type composition.

### Identify signaling groups based on their functional similarity

```
ptm = Sys.time()

cellchat <- computeNetSimilarityPairwise(cellchat, type = "functional")
#> Compute signaling network similarity for datasets 1 2
cellchat <- netEmbedding(cellchat, type = "functional")
#> Manifold learning of the signaling networks for datasets 1 2
cellchat <- netClustering(cellchat, type = "functional")
#> Classification learning of the signaling networks for datasets 1 2
# Visualization in 2D-space
netVisual_embeddingPairwise(cellchat, type = "functional", label.size = 3.5)
#> 2D visualization of signaling networks from datasets 1 2
```

```
# netVisual_embeddingZoomIn(cellchat, type = "functional", nCol = 2)
```

### Identify signaling groups based on structure similarity

```
cellchat <- computeNetSimilarityPairwise(cellchat, type = "structural")
cellchat <- netEmbedding(cellchat, type = "structural")
cellchat <- netClustering(cellchat, type = "structural")
# Visualization in 2D-space
netVisual_embeddingPairwise(cellchat, type = "structural", label.size = 3.5)
netVisual_embeddingPairwiseZoomIn(cellchat, type = "structural", nCol = 2)
```

### Compute and visualize the pathway distance in the learned joint manifold

CellChat can identify the signaling networks with larger (or smaller)
difference based on their Euclidean distance in the shared
two-dimensions space. Larger distance implies larger difference of the
communication networks between two datasets in terms of either
functional or structure similarity. It should be noted that we only
compute the distance of overlapped signaling pathways between two
datasets. Those signaling pathways that are only identified in one
dataset are not considered here. If there are more than three datasets,
one can do pairwise comparisons by modifying the parameter
`comparison` in the function `rankSimilarity`.

```
rankSimilarity(cellchat, type = "functional")
#> Compute the distance of signaling networks between datasets 1 2
```

## Identify altered signaling with distinct interaction strength

By comparing the information flow/interaction strength of each
signaling pathway, CellChat identifies signaling pathways that: (i) turn
off, (ii) decrease, (iii) turn on, or (iv) increase, by changing their
information flow at one condition as compared to another condition.
Identify the altered signaling pathways or ligand-receptor pairs based
on the overall information flow by following option A, and based on the
outgoing (or incoming) signaling patterns by following option B.

### (A) Compare the overall information flow of each signaling pathway or ligand-receptor pair

CellChat can identify the conserved and context-specific signaling
pathways by simply comparing the information flow for each signaling
pathway, which is defined by the sum of communication probability among
all pairs of cell groups in the inferred network (i.e., the total
weights in the network).

This bar chart can be plotted in a stacked mode or not. Significant
signaling pathways were ranked based on differences in the overall
information flow within the inferred networks between NL and LS skin.
When setting `do.stat = TRUE`, a paired Wilcoxon test is
performed to determine whether there is a significant difference of the
signaling information flow between two conditions. The top signaling
pathways colored red are enriched in NL skin, and these colored greens
are enriched in the LS skin.

```
gg1 <- rankNet(cellchat, mode = "comparison", measure = "weight", sources.use = NULL, targets.use = NULL, stacked = T, do.stat = TRUE)
gg2 <- rankNet(cellchat, mode = "comparison", measure = "weight", sources.use = NULL, targets.use = NULL, stacked = F, do.stat = TRUE)

gg1 + gg2
```

### (B) Compare outgoing (or incoming) signaling patterns associated with each cell population

The above analysis summarize the information from the outgoing and
incoming signaling together. CellChat can also compare the outgoing (or
incoming) signaling pattern between two datasets, allowing to identify
signaling pathways/ligand-receptors that exhibit different signaling
patterns. We can combine all the identified signaling pathways from
different datasets and thus compare them side by side, including
outgoing signaling, incoming signaling and overall signaling by
aggregating outgoing and incoming signaling together.

CellChat uses heatmap plot to show the contribution of signals
(signaling pathways or ligand-receptor pairs) to cell groups in terms of
outgoing or incoming signaling. In this heatmap, colobar represents the
relative signaling strength of a signaling pathway across cell groups
(Note that values are row-scaled). The top colored bar plot shows the
total signaling strength of a cell group by summarizing all signaling
pathways displayed in the heatmap. The right grey bar plot shows the
total signaling strength of a signaling pathway by summarizing all cell
groups displayed in the heatmap.

```
library(ComplexHeatmap)
#> Loading required package: grid
#> ========================================
#> ComplexHeatmap version 2.15.4
#> Bioconductor page: http://bioconductor.org/packages/ComplexHeatmap/
#> Github page: https://github.com/jokergoo/ComplexHeatmap
#> Documentation: http://jokergoo.github.io/ComplexHeatmap-reference
#> 
#> If you use it in published research, please cite either one:
#> - Gu, Z. Complex Heatmap Visualization. iMeta 2022.
#> - Gu, Z. Complex heatmaps reveal patterns and correlations in multidimensional 
#>     genomic data. Bioinformatics 2016.
#> 
#> 
#> The new InteractiveComplexHeatmap package can directly export static 
#> complex heatmaps into an interactive Shiny app with zero effort. Have a try!
#> 
#> This message can be suppressed by:
#>   suppressPackageStartupMessages(library(ComplexHeatmap))
#> ========================================
i = 1
# combining all the identified signaling pathways from different datasets 
pathway.union <- union(object.list[[i]]@netP$pathways, object.list[[i+1]]@netP$pathways)
ht1 = netAnalysis_signalingRole_heatmap(object.list[[i]], pattern = "outgoing", signaling = pathway.union, title = names(object.list)[i], width = 5, height = 6)
ht2 = netAnalysis_signalingRole_heatmap(object.list[[i+1]], pattern = "outgoing", signaling = pathway.union, title = names(object.list)[i+1], width = 5, height = 6)
draw(ht1 + ht2, ht_gap = unit(0.5, "cm"))
```

```
execution.time = Sys.time() - ptm
print(as.numeric(execution.time, units = "secs"))
#> [1] 17.62665
```

```
ht1 = netAnalysis_signalingRole_heatmap(object.list[[i]], pattern = "incoming", signaling = pathway.union, title = names(object.list)[i], width = 5, height = 6, color.heatmap = "GnBu")
ht2 = netAnalysis_signalingRole_heatmap(object.list[[i+1]], pattern = "incoming", signaling = pathway.union, title = names(object.list)[i+1], width = 5, height = 6, color.heatmap = "GnBu")
draw(ht1 + ht2, ht_gap = unit(0.5, "cm"))
```

```
ht1 = netAnalysis_signalingRole_heatmap(object.list[[i]], pattern = "all", signaling = pathway.union, title = names(object.list)[i], width = 5, height = 6, color.heatmap = "OrRd")
ht2 = netAnalysis_signalingRole_heatmap(object.list[[i+1]], pattern = "all", signaling = pathway.union, title = names(object.list)[i+1], width = 5, height = 6, color.heatmap = "OrRd")
draw(ht1 + ht2, ht_gap = unit(0.5, "cm"))
```

# Part III: Identify the up-gulated and down-regulated signaling ligand-receptor pairs

## Identify dysfunctional signaling by comparing the communication probabities

CellChat can compare the communication probabilities mediated by L-R
pairs from certain cell groups to other cell groups. This can be done by
setting `comparison` in the function
`netVisual_bubble`.

```
ptm = Sys.time()

netVisual_bubble(cellchat, sources.use = 4, targets.use = c(5:11),  comparison = c(1, 2), angle.x = 45)
#> Comparing communications on a merged object
```

Moreover, CellChat can identify the up-regulated (increased) and
down-regulated (decreased) signaling ligand-receptor pairs in one
dataset compared to the other dataset. This can be done by specifying
`max.dataset` and `min.dataset` in the function
`netVisual_bubble`. The increased signaling means these
signaling have higher communication probability (strength) in the second
dataset compared to the first dataset. The ligand-receptor pairs shown
in the bubble plot can be accessed via `gg1$data`.

```
gg1 <- netVisual_bubble(cellchat, sources.use = 4, targets.use = c(5:11),  comparison = c(1, 2), max.dataset = 2, title.name = "Increased signaling in LS", angle.x = 45, remove.isolate = T)
#> Comparing communications on a merged object
gg2 <- netVisual_bubble(cellchat, sources.use = 4, targets.use = c(5:11),  comparison = c(1, 2), max.dataset = 1, title.name = "Decreased signaling in LS", angle.x = 45, remove.isolate = T)
#> Comparing communications on a merged object
gg1 + gg2
```

NB: The ligand-receptor pairs shown in the bubble plot can be accessed
via `signaling.LSIncreased = gg1$data`.

## Identify dysfunctional signaling by using differential expression analysis

The above method for identifying the upgulated and down-regulated
signaling is perfomed by comparing the communication probability between
two datasets for each L-R pair and each pair of cell groups.
Alternative, we can identify the upgulated and down-regulated signaling
ligand-receptor pairs based on the differential expression analysis
(DEA). Specifically, we perform differential expression analysis between
two biological conditions (i.e., NL and LS) for each cell group, and
then obtain the upgulated and down-regulated signaling based on the fold
change of ligands in the sender cells and receptors in the receiver
cells.

Of note, users may observe the same LR pairs appearing in both the
up-regulated and down-regulated results due to the fact that DEA between
conditions is performed for each cell group. To perform DEA between
conditions by ignoring cell group information, users can set
`group.DE.combined = TRUE` in
`identifyOverExpressedGenes` for CellChat v2.1.1.

```
# define a positive dataset, i.e., the dataset with positive fold change against the other dataset
pos.dataset = "LS"
# define a char name used for storing the results of differential expression analysis
features.name = paste0(pos.dataset, ".merged")

# perform differential expression analysis 
# Of note, compared to CellChat version < v2, CellChat v2 now performs an ultra-fast Wilcoxon test using the presto package, which gives smaller values of logFC. Thus we here set a smaller value of thresh.fc compared to the original one (thresh.fc = 0.1). Users can also provide a vector and dataframe of customized DEGs by modifying the cellchat@var.features$LS.merged and cellchat@var.features$LS.merged.info. 

cellchat <- identifyOverExpressedGenes(cellchat, group.dataset = "datasets", pos.dataset = pos.dataset, features.name = features.name, only.pos = FALSE, thresh.pc = 0.1, thresh.fc = 0.05,thresh.p = 0.05, group.DE.combined = FALSE) 
#> Use the joint cell labels from the merged CellChat object

# map the results of differential expression analysis onto the inferred cell-cell communications to easily manage/subset the ligand-receptor pairs of interest
net <- netMappingDEG(cellchat, features.name = features.name, variable.all = TRUE)
# extract the ligand-receptor pairs with upregulated ligands in LS
net.up <- subsetCommunication(cellchat, net = net, datasets = "LS",ligand.logFC = 0.05, receptor.logFC = NULL)
# extract the ligand-receptor pairs with upregulated ligands and upregulated receptors in NL, i.e.,downregulated in LS
net.down <- subsetCommunication(cellchat, net = net, datasets = "NL",ligand.logFC = -0.05, receptor.logFC = NULL)
```

Since the signaling genes in the `net.up` and
`net.down` might be complex with multi-subunits, we can do
further deconvolution to obtain the individual signaling genes.

```
gene.up <- extractGeneSubsetFromPair(net.up, cellchat)
gene.down <- extractGeneSubsetFromPair(net.down, cellchat)
```

Users can also find all the significant outgoing/incoming/both
signaling according to the customized features and cell groups of
interest

```
df <- findEnrichedSignaling(object.list[[2]], features = c("CCL19", "CXCL12"), idents = c("Inflam. FIB", "COL11A1+ FIB"), pattern ="outgoing")
```

## Visualize the identified up-regulated and down-regulated signaling ligand-receptor pairs

CellChat can visualize the identified up-regulated and down-regulated
signaling ligand-receptor pairs using bubble plot (option A), chord
diagram (option B) or wordcloud (option C).

### (A) Bubble plot

We then visualize the upgulated and down-regulated signaling
ligand-receptor pairs using bubble plot or chord diagram.

```
pairLR.use.up = net.up[, "interaction_name", drop = F]
gg1 <- netVisual_bubble(cellchat, pairLR.use = pairLR.use.up, sources.use = 4, targets.use = c(5:11), comparison = c(1, 2),  angle.x = 90, remove.isolate = T,title.name = paste0("Up-regulated signaling in ", names(object.list)[2]))
#> Comparing communications on a merged object
pairLR.use.down = net.down[, "interaction_name", drop = F]
gg2 <- netVisual_bubble(cellchat, pairLR.use = pairLR.use.down, sources.use = 4, targets.use = c(5:11), comparison = c(1, 2),  angle.x = 90, remove.isolate = T,title.name = paste0("Down-regulated signaling in ", names(object.list)[2]))
#> Comparing communications on a merged object
gg1 + gg2
```

### (B) Chord diagram

Visualize the upgulated and down-regulated signaling ligand-receptor
pairs using Chord diagram

```
# Chord diagram
par(mfrow = c(1,2), xpd=TRUE)
netVisual_chord_gene(object.list[[2]], sources.use = 4, targets.use = c(5:11), slot.name = 'net', net = net.up, lab.cex = 0.8, small.gap = 3.5, title.name = paste0("Up-regulated signaling in ", names(object.list)[2]))
netVisual_chord_gene(object.list[[1]], sources.use = 4, targets.use = c(5:11), slot.name = 'net', net = net.down, lab.cex = 0.8, small.gap = 3.5, title.name = paste0("Down-regulated signaling in ", names(object.list)[2]))
#> You may try the function `netVisual_chord_cell` for visualizing individual signaling pathway
```

### (C) Wordcloud plot

Visualize the enriched ligands, signaling,or ligand-receptor pairs in
one condition compared to another condition using wordcloud

```
# visualize the enriched ligands in the first condition
computeEnrichmentScore(net.down, species = 'human', variable.both = TRUE)
```

```
# visualize the enriched ligands in the second condition
computeEnrichmentScore(net.up, species = 'human', variable.both = TRUE)
```

# Part IV: Visually compare cell-cell communication using Hierarchy plot, Circle plot or Chord diagram

Similar to the CellChat
analysis of individual dataset, CellChat can visually compare
cell-cell communication networks using hierarchy plot, circle plot,
chord diagram, or heatmap. More details on the visualization can be
found in the CellChat
analysis of individual dataset.

**Edge color/weight, node color/size/shape**: In all
visualization plots, edge colors are consistent with the sources as
sender, and edge weights are proportional to the interaction strength.
Thicker edge line indicates a stronger signal. In the **Hierarchy
plot and Circle plot**, circle sizes are proportional to the
number of cells in each cell group. In the hierarchy plot, solid and
open circles represent source and target, respectively. In the
**Chord diagram**, the inner thinner bar colors represent
the targets that receive signal from the corresponding outer bar. The
inner bar size is proportional to the signal strength received by the
targets. Such inner bar is helpful for interpreting the complex chord
diagram. Note that there exist some inner bars without any chord for
some cell groups, please just igore it because this is an issue that has
not been addressed by circlize package.

```
pathways.show <- c("CXCL") 
weight.max <- getMaxWeight(object.list, slot.name = c("netP"), attribute = pathways.show) # control the edge weights across different datasets
par(mfrow = c(1,2), xpd=TRUE)
for (i in 1:length(object.list)) {
  netVisual_aggregate(object.list[[i]], signaling = pathways.show, layout = "circle", edge.weight.max = weight.max[1], edge.width.max = 10, signaling.name = paste(pathways.show, names(object.list)[i]))
}
```

```
pathways.show <- c("CXCL") 
par(mfrow = c(1,2), xpd=TRUE)
ht <- list()
for (i in 1:length(object.list)) {
  ht[[i]] <- netVisual_heatmap(object.list[[i]], signaling = pathways.show, color.heatmap = "Reds",title.name = paste(pathways.show, "signaling ",names(object.list)[i]))
}
#> Do heatmap based on a single object 
#> 
#> Do heatmap based on a single object
ComplexHeatmap::draw(ht[[1]] + ht[[2]], ht_gap = unit(0.5, "cm"))
```

```
# Chord diagram
pathways.show <- c("CXCL") 
par(mfrow = c(1,2), xpd=TRUE)
for (i in 1:length(object.list)) {
  netVisual_aggregate(object.list[[i]], signaling = pathways.show, layout = "chord", signaling.name = paste(pathways.show, names(object.list)[i]))
}
```

For the chord diagram, CellChat has an independent function
`netVisual_chord_cell` to flexibly visualize the signaling
network by adjusting different parameters in the circlize package. For
example, we can define a named char vector `group` to create
multiple-group chord diagram, e.g., grouping cell clusters into
different cell types.

```
# Chord diagram
group.cellType <- c(rep("FIB", 4), rep("DC", 4), rep("TC", 4)) # grouping cell clusters into fibroblast, DC and TC cells
names(group.cellType) <- levels(object.list[[1]]@idents)
pathways.show <- c("CXCL") 
par(mfrow = c(1,2), xpd=TRUE)
for (i in 1:length(object.list)) {
  netVisual_chord_cell(object.list[[i]], signaling = pathways.show, group = group.cellType, title.name = paste0(pathways.show, " signaling network - ", names(object.list)[i]))
}
#> Plot the aggregated cell-cell communication network at the signaling pathway level
#> Plot the aggregated cell-cell communication network at the signaling pathway level
```

Using chord diagram, CellChat provides two functions
`netVisual_chord_cell` and `netVisual_chord_gene`
for visualizing cell-cell communication with different purposes and
different levels. `netVisual_chord_cell` is used for
visualizing the cell-cell communication between different cell groups
(where each sector in the chord diagram is a cell group), and
`netVisual_chord_gene` is used for visualizing the cell-cell
communication mediated by mutiple ligand-receptors or signaling pathways
(where each sector in the chord diagram is a ligand, receptor or
signaling pathway.)

```
par(mfrow = c(1, 2), xpd=TRUE)
# compare all the interactions sending from Inflam.FIB to DC cells
for (i in 1:length(object.list)) {
  netVisual_chord_gene(object.list[[i]], sources.use = 4, targets.use = c(5:8), lab.cex = 0.5, title.name = paste0("Signaling from Inflam.FIB - ", names(object.list)[i]))
}
```

```
# compare all the interactions sending from fibroblast to inflamatory immune cells
par(mfrow = c(1, 2), xpd=TRUE)
for (i in 1:length(object.list)) {
  netVisual_chord_gene(object.list[[i]], sources.use = c(1,2, 3, 4), targets.use = c(8,10),  title.name = paste0("Signaling received by Inflam.DC and .TC - ", names(object.list)[i]), legend.pos.x = 10)
}
```

```
# show all the significant signaling pathways from fibroblast to immune cells
par(mfrow = c(1, 2), xpd=TRUE)
for (i in 1:length(object.list)) {
  netVisual_chord_gene(object.list[[i]], sources.use = c(1,2,3,4), targets.use = c(5:11),slot.name = "netP", title.name = paste0("Signaling pathways sending from fibroblast - ", names(object.list)[i]), legend.pos.x = 10)
}
```

NB: Please ignore the note when generating the plot such as “Note: The
first link end is drawn out of sector ‘MIF’.”. If the gene names are
overlapped, you can adjust the argument `small.gap` by
decreasing the value.

# Part V: Compare the signaling gene expression distribution between different datasets

We can plot the gene expression distribution of signaling genes
related to L-R pairs or signaling pathway using a Seurat wrapper
function `plotGeneExpression`.

```
cellchat@meta$datasets = factor(cellchat@meta$datasets, levels = c("NL", "LS")) # set factor level
plotGeneExpression(cellchat, signaling = "CXCL", split.by = "datasets", colors.ggplot = T, type = "violin")
#> The legacy packages maptools, rgdal, and rgeos, underpinning the sp package,
#> which was just loaded, were retired in October 2023.
#> Please refer to R-spatial evolution reports for details, especially
#> https://r-spatial.org/r/2023/05/15/evolution4.html.
#> It may be desirable to make the sf package available;
#> package maintainers should consider adding sf to Suggests:.
#> The default behaviour of split.by has changed.
#> Separate violin plots are now plotted side-by-side.
#> To restore the old behaviour of a single split violin,
#> set split.plot = TRUE.
#>       
#> This message will be shown once per session.
#> Scale for y is already present.
#> Adding another scale for y, which will replace the existing scale.
#> Scale for y is already present.
#> Adding another scale for y, which will replace the existing scale.
#> Scale for y is already present.
#> Adding another scale for y, which will replace the existing scale.
```

```
execution.time = Sys.time() - ptm
print(as.numeric(execution.time, units = "secs"))
#> [1] 7.094303
```

# Save the merged CellChat object

```
save(object.list, file = "cellchat_object.list_humanSkin_NL_LS.RData")
save(cellchat, file = "cellchat_merged_humanSkin_NL_LS.RData")
```

```
sessionInfo()
#> R version 4.3.1 (2023-06-16)
#> Platform: aarch64-apple-darwin20 (64-bit)
#> Running under: macOS Ventura 13.5
#> 
#> Matrix products: default
#> BLAS:   /Library/Frameworks/R.framework/Versions/4.3-arm64/Resources/lib/libRblas.0.dylib 
#> LAPACK: /Library/Frameworks/R.framework/Versions/4.3-arm64/Resources/lib/libRlapack.dylib;  LAPACK version 3.11.0
#> 
#> locale:
#> [1] en_US.UTF-8/en_US.UTF-8/en_US.UTF-8/C/en_US.UTF-8/en_US.UTF-8
#> 
#> time zone: Asia/Shanghai
#> tzcode source: internal
#> 
#> attached base packages:
#> [1] grid      stats     graphics  grDevices utils     datasets  methods  
#> [8] base     
#> 
#> other attached packages:
#> [1] ComplexHeatmap_2.15.4 patchwork_1.1.3       CellChat_2.1.2       
#> [4] Biobase_2.60.0        BiocGenerics_0.46.0   ggplot2_3.4.3        
#> [7] igraph_1.5.1          dplyr_1.1.3          
#> 
#> loaded via a namespace (and not attached):
#>   [1] RcppAnnoy_0.0.21       splines_4.3.1          later_1.3.1           
#>   [4] tibble_3.2.1           polyclip_1.10-4        ggnetwork_0.5.12      
#>   [7] fastDummies_1.7.3      lifecycle_1.0.3        rstatix_0.7.2.999     
#>  [10] doParallel_1.0.17      rprojroot_2.0.3        globals_0.16.2        
#>  [13] lattice_0.21-8         MASS_7.3-60            backports_1.4.1       
#>  [16] magrittr_2.0.3         plotly_4.10.2          sass_0.4.7            
#>  [19] rmarkdown_2.24         jquerylib_0.1.4        yaml_2.3.7            
#>  [22] httpuv_1.6.11          Seurat_5.0.1           sctransform_0.4.1     
#>  [25] NMF_0.26               spam_2.9-1             spatstat.sparse_3.0-2 
#>  [28] sp_2.1-0               reticulate_1.31        cowplot_1.1.1         
#>  [31] pbapply_1.7-2          RColorBrewer_1.1-3     abind_1.4-5           
#>  [34] Rtsne_0.16             purrr_1.0.2            presto_1.0.0          
#>  [37] circlize_0.4.16        IRanges_2.34.1         S4Vectors_0.38.1      
#>  [40] ggrepel_0.9.3          irlba_2.3.5.1          spatstat.utils_3.0-3  
#>  [43] listenv_0.9.0          goftest_1.2-3          RSpectra_0.16-1       
#>  [46] spatstat.random_3.1-5  fitdistrplus_1.1-11    parallelly_1.36.0     
#>  [49] svglite_2.1.1          leiden_0.4.3           codetools_0.2-19      
#>  [52] tidyselect_1.2.0       shape_1.4.6            farver_2.1.1          
#>  [55] spatstat.explore_3.2-1 matrixStats_1.0.0      stats4_4.3.1          
#>  [58] jsonlite_1.8.7         GetoptLong_1.0.5       BiocNeighbors_1.18.0  
#>  [61] ellipsis_0.3.2         progressr_0.14.0       ggridges_0.5.4        
#>  [64] ggalluvial_0.12.5      survival_3.5-7         iterators_1.0.14      
#>  [67] systemfonts_1.0.4      foreach_1.5.2          tools_4.3.1           
#>  [70] ragg_1.2.5             sna_2.7-1              ica_1.0-3             
#>  [73] Rcpp_1.0.11            glue_1.6.2             gridExtra_2.3         
#>  [76] xfun_0.40              here_1.0.1             withr_2.5.0           
#>  [79] BiocManager_1.30.22    fastmap_1.1.1          fansi_1.0.4           
#>  [82] digest_0.6.33          R6_2.5.1               mime_0.12             
#>  [85] textshaping_0.3.6      colorspace_2.1-0       scattermore_1.2       
#>  [88] tensor_1.5             spatstat.data_3.0-1    utf8_1.2.3            
#>  [91] tidyr_1.3.0            generics_0.1.3         data.table_1.14.9     
#>  [94] FNN_1.1.3.2            httr_1.4.7             htmlwidgets_1.6.2     
#>  [97] uwot_0.1.16            pkgconfig_2.0.3        gtable_0.3.4          
#> [100] registry_0.5-1         lmtest_0.9-40          htmltools_0.5.6       
#> [103] carData_3.0-5          dotCall64_1.0-2        clue_0.3-64           
#> [106] SeuratObject_5.0.1     scales_1.2.1           tidyverse_2.0.0       
#> [109] png_0.1-8              wordcloud_2.6          knitr_1.43            
#> [112] rstudioapi_0.15.0      reshape2_1.4.4         rjson_0.2.21          
#> [115] nlme_3.1-163           coda_0.19-4            statnet.common_4.9.0  
#> [118] cachem_1.0.8           zoo_1.8-12             GlobalOptions_0.1.2   
#> [121] stringr_1.5.0          KernSmooth_2.23-22     parallel_4.3.1        
#> [124] miniUI_0.1.1.1         pillar_1.9.0           vctrs_0.6.3           
#> [127] RANN_2.6.1             promises_1.2.1         ggpubr_0.6.0          
#> [130] car_3.1-2              xtable_1.8-4           cluster_2.1.4         
#> [133] evaluate_0.21          magick_2.8.1           cli_3.6.1             
#> [136] compiler_4.3.1         rlang_1.1.1            crayon_1.5.2          
#> [139] rngtools_1.5.2         future.apply_1.11.0    ggsignif_0.6.4        
#> [142] labeling_0.4.3         plyr_1.8.8             stringi_1.7.12        
#> [145] deldir_1.0-9           viridisLite_0.4.2      network_1.18.1        
#> [148] gridBase_0.4-7         BiocParallel_1.34.2    munsell_0.5.0         
#> [151] lazyeval_0.2.2         spatstat.geom_3.2-4    Matrix_1.6-5          
#> [154] RcppHNSW_0.5.0         future_1.33.0          shiny_1.7.5           
#> [157] highr_0.10             ROCR_1.0-11            broom_1.0.5           
#> [160] bslib_0.5.1
```
