# Supplementary material for: CSNK2B Expression Aligns with a GDF15-Associated Immune Molecular State in Hepatocellular Carcinoma
Source: Biomedicines. 2026 Aug 26;14(9):1909. doi: 10.3390/biomedicines14091909 (PMC13604900; doi:10.3390/biomedicines14091909)
Supplement: Supplementary file 1 [file biomedicines-14-01909-s001.zip › Supplemental file S1/docker_cellchat/CellChat-main-http1.tar.gz!/CellChat-main/tutorial/Comparison_analysis_of_multiple_datasets_with_different_cellular_compositions.html]

Comparison analysis of multiple datasets with different cell type compositions


# Comparison analysis of multiple datasets with different cell type compositions

#### Suoqin Jin

#### 14 February, 2024

- Load the required
  libraries
- Part
  I: Comparative analysis of multiple datasets with slightly different
  cell type compositions
  - Load CellChat object of
    each dataset
  - Lift up
    CellChat objects and merge them together
  - Comparative
    visualization and analysis of cell-cell communication using the lifted
    objects
- Part
  II: Comparison analysis of multiple datasets with vastly different cell
  type compositions

This vignette shows how to apply CellChat to the comparison analysis
of multiple datasets with different cell type compositions. Almost all
the CellChat’s functionalities can be applied.

## Load the required libraries

```
library(CellChat)
library(patchwork)
```

# Part I: Comparative analysis of multiple datasets with slightly different cell type compositions

For the datasets with differing cell type (group) compositions,
CellChat lifts up the cell groups to the same cell type compositions
across all datasets using the function `liftCellChat`, and
then performs comparative analysis as the joint analysis of datasets
with the same cell type compositions.

Here we take an example of comparative analysis of two embryonic
mouse skin scRNA-seq datasets from days E13.5 and E14.5. There are 11
shared skin cell populations at E13.5 and E14.5 and additional two
populations (i.e., dermal DC and pericytes) specific to E14.5.
Therefore, we lift up the cell groups from E13.5 to the same cell labels
as E14.5.

## Load CellChat object of each dataset

Users need to run CellChat on each dataset separately and then merge
different CellChat objects together. Here we do
`updateCellChat` because these two objects are obtained using
the earlier version (< 1.6.0) of CellChat.

```
ptm = Sys.time()

cellchat.E13 <- readRDS("/Users/suoqinjin/Library/CloudStorage/OneDrive-Personal/works/CellChat/tutorial/cellchat_embryonic_E13.rds")
cellchat.E13 <- updateCellChat(cellchat.E13)
#> Update slot 'var.features' from a vector to a list
#> Warning in updateCellChat(cellchat.E13): The 'meta' data does not have a column
#> named `samples`. We now add this column and all cells are assumed to belong to
#> `sample1`!
cellchat.E14 <- readRDS("/Users/suoqinjin/Library/CloudStorage/OneDrive-Personal/works/CellChat/tutorial/cellchat_embryonic_E14.rds")
cellchat.E14 <- updateCellChat(cellchat.E14)
#> Update slot 'var.features' from a vector to a list
#> Warning in updateCellChat(cellchat.E14): The 'meta' data does not have a column
#> named `samples`. We now add this column and all cells are assumed to belong to
#> `sample1`!
```

## Lift up CellChat objects and merge them together

Since there are two additional populations (i.e., dermal DC and
pericytes) specific to E14.5 compared to E13.5, we lift up
`cellchat.E13` by lifting up the cell groups to the same cell
group compositions as E14.5. `liftCellChat` only updates the
slot related to cell-cell communication network, including slots
`object@net`, `object@netP` and
`object@idents`.

```
# Define the cell labels to lift up by combining both cell labels from the conditions
# Please note that the order of cell groups in `group.new` will affect the appearance order when visualizing the cell-cell communication. If there are unique cell groups in both the conditions, you should define `group.new = union(levels(cellchat.E14@idents), levels(cellchat.E13@idents))`
group.new = levels(cellchat.E14@idents)
cellchat.E13 <- liftCellChat(cellchat.E13, group.new)
#> The CellChat object will be lifted up using the cell labels FIB-A, FIB-B, FIB-P, DC, Pericyte, MYL, Immune, ENDO, Muscle, MELA, Basal-P, Basal, Spinious
#> Update slots object@net, object@netP, object@idents in a single dataset...
# Of note, we did not apply `liftCellChat` to cellchat.E14 here because it contains all cell groups. 
object.list <- list(E13 = cellchat.E13, E14 = cellchat.E14)
cellchat <- mergeCellChat(object.list, add.names = names(object.list), cell.prefix = TRUE)
#> Warning in mergeCellChat(object.list, add.names = names(object.list),
#> cell.prefix = TRUE): Prefix cell names!
#> The cell barcodes in merged 'meta' is  rep1_AAACCTGCACCAACCG rep1_AAACGGGAGCCGATTT rep1_AAACGGGAGTATCGAA rep1_AAACGGGCATCTCCCA rep1_AAAGATGCACTTGGAT rep1_AAAGATGCAGTTCATG
#> Warning in mergeCellChat(object.list, add.names = names(object.list), cell.prefix = TRUE): The cell barcodes in merged 'meta' is different from those in the used data matrix.
#>               We now simply assign the colnames in the data matrix to the rownames of merged 'mata'!
#> Merge the following slots: 'data.signaling','images','net', 'netP','meta', 'idents', 'var.features' , 'DB', and 'LR'.
```

## Comparative visualization and analysis of cell-cell communication using the lifted objects

Once we lift up the CellChat object and merge different objects
together, we can perform CellChat analysis the same way as the
comparative analysis of multiple datasets with the same cell type
compositions (see the tutorial
on Comparison analysis of multiple datasets). Below is an example of
how to compare the inferred cell-cell communication networks using
Circle plot, Hierarchy plot and Chord diagram.

```
# Circle plot
pathways.show <- c("WNT") 
weight.max <- getMaxWeight(object.list, slot.name = c("netP"), attribute = pathways.show) # control the edge weights across different datasets
par(mfrow = c(1,2), xpd=TRUE)
for (i in 1:length(object.list)) {
  netVisual_aggregate(object.list[[i]], signaling = pathways.show, layout = "circle", edge.weight.max = weight.max[1], edge.width.max = 10, signaling.name = paste(pathways.show, names(object.list)[i]))
}
```

```
execution.time = Sys.time() - ptm
print(as.numeric(execution.time, units = "secs"))
#> [1] 4.815936
```

```
# Hierarchy plot
pathways.show <- c("WNT") 
weight.max <- getMaxWeight(object.list, slot.name = c("netP"), attribute = pathways.show) # control the edge weights across different datasets
vertex.receiver = seq(1,10) # Left portion of hierarchy plot the shows signaling to dermal cells and right portion shows signaling to epidermal cells
par(mfrow = c(1,2), xpd=TRUE)
for (i in 1:length(object.list)) {
  netVisual_aggregate(object.list[[i]], signaling = pathways.show, vertex.receiver = vertex.receiver, edge.weight.max = weight.max[1], edge.width.max = 10, signaling.name = paste(pathways.show, names(object.list)[i]))
}
```

```
# Chord diagram
pathways.show <- c("WNT") 
par(mfrow = c(1,2), xpd=TRUE)
for (i in 1:length(object.list)) {
  netVisual_aggregate(object.list[[i]], signaling = pathways.show, layout = "chord", signaling.name = paste(pathways.show, names(object.list)[i]))
}
```

For the chord diagram, CellChat has an independent function
`netVisual_chord_cell` to flexibly visualize the signaling
network by adjusting different parameters in the circlize package. For
example, we can define a named char vector `group` to create
multiple-group chord diagram, e.g., grouping cell clusters into
different cell types.

```
# Chord diagram
group.merged <- c(rep("Dermal", 10), rep("Epidermal", 3)) # grouping cell clusters into dermal and epidermal cells to study the cell-cell communication between dermal and epidermal
names(group.merged) <- levels(object.list[[1]]@idents)
pathways.show <- c("WNT") 
par(mfrow = c(1,2), xpd=TRUE)
for (i in 1:length(object.list)) {
  netVisual_chord_cell(object.list[[i]], signaling = pathways.show, group = group.merged, title.name = paste0(pathways.show, " signaling network - ", names(object.list)[i]))
}
#> Plot the aggregated cell-cell communication network at the signaling pathway level
#> Plot the aggregated cell-cell communication network at the signaling pathway level
```

# Part II: Comparison analysis of multiple datasets with vastly different cell type compositions

CellChat can be used to compare cell-cell communication patterns
between two scRNA-seq datasets from vastly distinct biological contexts
– embryonic morphogenesis vs. wound-induced repair. For the datasets
with vastly different cell type (group) compositions, most of the
CellChat’s functionalities can be applied, except for the following two
aspects:

- Compare the differential number of interactions and interaction
  strength among different cell populations using
  `netVisual_diffInteraction`. However, USERS can still use
  `netVisual_circle` fucntion to show the number of
  interactions and interaction strength.
- Identify signaling groups based on their functional similarity
  using
  `computeNetSimilarityPairwise(cellchat, type = "functional")`.

However, USERS can still use
`computeNetSimilarityPairwise(cellchat, type = "structural")`
to perform structure similarity analysis.

Please check out our paper for the comparison analysis of two
scRNA-seq datasets, one from embryonic day E13.5 skin and another from
adult day 12 wound skin.

```
sessionInfo()
#> R version 4.3.1 (2023-06-16)
#> Platform: aarch64-apple-darwin20 (64-bit)
#> Running under: macOS Ventura 13.5
#> 
#> Matrix products: default
#> BLAS:   /Library/Frameworks/R.framework/Versions/4.3-arm64/Resources/lib/libRblas.0.dylib 
#> LAPACK: /Library/Frameworks/R.framework/Versions/4.3-arm64/Resources/lib/libRlapack.dylib;  LAPACK version 3.11.0
#> 
#> locale:
#> [1] en_US.UTF-8/en_US.UTF-8/en_US.UTF-8/C/en_US.UTF-8/en_US.UTF-8
#> 
#> time zone: Asia/Shanghai
#> tzcode source: internal
#> 
#> attached base packages:
#> [1] stats     graphics  grDevices utils     datasets  methods   base     
#> 
#> other attached packages:
#> [1] patchwork_1.1.3     CellChat_2.1.2      Biobase_2.60.0     
#> [4] BiocGenerics_0.46.0 ggplot2_3.4.3       igraph_1.5.1       
#> [7] dplyr_1.1.3        
#> 
#> loaded via a namespace (and not attached):
#>   [1] pbapply_1.7-2         rlang_1.1.1           magrittr_2.0.3       
#>   [4] clue_0.3-64           GetoptLong_1.0.5      gridBase_0.4-7       
#>   [7] matrixStats_1.0.0     compiler_4.3.1        systemfonts_1.0.4    
#>  [10] png_0.1-8             vctrs_0.6.3           reshape2_1.4.4       
#>  [13] ggalluvial_0.12.5     stringr_1.5.0         pkgconfig_2.0.3      
#>  [16] shape_1.4.6           crayon_1.5.2          fastmap_1.1.1        
#>  [19] backports_1.4.1       ellipsis_0.3.2        utf8_1.2.3           
#>  [22] promises_1.2.1        rmarkdown_2.24        network_1.18.1       
#>  [25] purrr_1.0.2           xfun_0.40             cachem_1.0.8         
#>  [28] jsonlite_1.8.7        highr_0.10            later_1.3.1          
#>  [31] BiocParallel_1.34.2   irlba_2.3.5.1         broom_1.0.5          
#>  [34] parallel_4.3.1        cluster_2.1.4         R6_2.5.1             
#>  [37] bslib_0.5.1           stringi_1.7.12        RColorBrewer_1.1-3   
#>  [40] reticulate_1.31       car_3.1-2             parallelly_1.36.0    
#>  [43] jquerylib_0.1.4       Rcpp_1.0.11           iterators_1.0.14     
#>  [46] knitr_1.43            future.apply_1.11.0   IRanges_2.34.1       
#>  [49] FNN_1.1.3.2           httpuv_1.6.11         Matrix_1.6-5         
#>  [52] tidyselect_1.2.0      abind_1.4-5           rstudioapi_0.15.0    
#>  [55] yaml_2.3.7            doParallel_1.0.17     codetools_0.2-19     
#>  [58] listenv_0.9.0         lattice_0.21-8        tibble_3.2.1         
#>  [61] plyr_1.8.8            shiny_1.7.5           withr_2.5.0          
#>  [64] coda_0.19-4           evaluate_0.21         future_1.33.0        
#>  [67] circlize_0.4.16       pillar_1.9.0          BiocManager_1.30.22  
#>  [70] ggpubr_0.6.0          carData_3.0-5         rngtools_1.5.2       
#>  [73] foreach_1.5.2         stats4_4.3.1          generics_0.1.3       
#>  [76] S4Vectors_0.38.1      munsell_0.5.0         scales_1.2.1         
#>  [79] NMF_0.26              ggnetwork_0.5.12      globals_0.16.2       
#>  [82] xtable_1.8-4          glue_1.6.2            tools_4.3.1          
#>  [85] data.table_1.14.9     BiocNeighbors_1.18.0  RSpectra_0.16-1      
#>  [88] ggsignif_0.6.4        registry_0.5-1        cowplot_1.1.1        
#>  [91] grid_4.3.1            tidyr_1.3.0           tidyverse_2.0.0      
#>  [94] colorspace_2.1-0      presto_1.0.0          cli_3.6.1            
#>  [97] fansi_1.0.4           svglite_2.1.1         ComplexHeatmap_2.15.4
#> [100] gtable_0.3.4          rstatix_0.7.2.999     sass_0.4.7           
#> [103] digest_0.6.33         ggrepel_0.9.3         sna_2.7-1            
#> [106] rjson_0.2.21          htmltools_0.5.6       lifecycle_1.0.3      
#> [109] statnet.common_4.9.0  GlobalOptions_0.1.2   mime_0.12
```
