# Supplementary material for: CSNK2B Expression Aligns with a GDF15-Associated Immune Molecular State in Hepatocellular Carcinoma
Source: Biomedicines. 2026 Aug 26;14(9):1909. doi: 10.3390/biomedicines14091909 (PMC13604900; doi:10.3390/biomedicines14091909)
Supplement: Supplementary file 1 [file biomedicines-14-01909-s001.zip › Supplemental file S1/docker_cellchat/CellChat-main-http1.tar.gz!/CellChat-main/tutorial/FAQ_on_applying_CellChat_to_spatial_transcriptomics_data.html]

FAQ on applying CellChat to spatially resolved transcriptomics data


# FAQ on applying CellChat to spatially resolved transcriptomics data

#### Suoqin Jin

- 10X Visium
- Xenium/visium
  HD/seqFISH/merFISH/STARmap
- Slide-seq
- CosMx
- Stereo-seq
- Application
  to a dataset with a small panel of genes
- Application
  to multiple spatial transcriptomics datasets

This vignette outlines Frequently Asked Questions (FAQ) when applying
CellChat to spatially resolved transcriptomics data, particularly on
**how to set the `spatial.factors` for different types
of spatial transcriptomics data**.

When inferring spatially-proximal cell-cell communication from
spatially resolved transcriptomic data, user also should provide spatial
coordinates/locations of spot/cell centroids. In addition, to filter out
cell-cell communication beyond the maximum diffusion range of molecules
(e.g., ~250μm), CellChat needs to compute the cell centroid-to-centroid
distance in the unit of micrometers. Therefore, for spatial technologies
that only provide spatial coordinates in pixels, CellChat converts
spatial coordinates from pixels to micrometers by requiring users to
input the conversion factor.

To infer spatially proximal cell-cell communication, in addition to
the gene expression data matrix, CellChat requires additional three user
inputs:

- **(1) `meta` (User assigned cell labels and
  samples labels)**: a data frame (rows are cells with rownames)
  consisting of cell information, which will be used for defining cell
  groups. A column named `samples` should be provided for
  spatial transcriptomics analysis, which is useful for analyzing
  cell-cell communication by aggregating multiple samples/replicates. Of
  note, for comparison analysis across different conditions, users still
  need to create a CellChat object seperately for each condition.
- **(2) `coordinates` (Spatial coordinates of
  spots/cells)**: a data frame in which each row gives the spatial
  coordinates/locations of each cell/spot centroid.
- **(3) `spatial.factors` (Spatial factors of
  spatial distance)**: a data frame containing two distance factors
  `ratio` and `tol`, which is dependent on spatial
  transcriptomics technologies (and specific datasets) and detailed as
  follows:
- 1. `ratio`: the conversion factor when converting spatial
     coordinates from Pixels or other units to Micrometers (i.e.,Microns).
     For example, setting `ratio = 0.18` indicates that 1 pixel
     equals 0.18um in the coordinates.
- 2. `tol`: the tolerance factor to increase the robustness
     when comparing the center-to-center distance against the
     `interaction.range`. This can be the half value of cell/spot
     size in the unit of um. If the cell/spot size is not known, we provide a
     function `computeCellDistance` to compute the cell
     center-to-center distance. `tol` can be the the half value of
     the minimum center-to-center distance. Of note, CellChat does not need
     an accurate tolerance factor, which is used for determining whether
     considering the cell-pair as spatially proximal if their distance is
     greater than `interaction.range` but smaller than
     “`interaction.range` + `tol`”.

When inferring contact-dependent or juxtacrine signaling by setting
`contact.dependent = TRUE` in `computeCommunProb`,
and using L-R pairs from `Cell-Cell Contact` signaling
classified in `CellChatDB$interaction$annotation`, CellChat
requires another one user input:

- **(4) `contact.range`**: a value giving the
  interaction range (Unit: microns) to restrict the contact-dependent
  signaling. For spatial transcriptomics in a single-cell resolution,
  `contact.range` is approximately equal to the estimated cell
  diameter (i.e., the cell center-to-center distance), which means that
  contact-dependent or juxtacrine signaling can only happens when the two
  cells are contact to each other. Typically,
  `contact.range = 10`, which is a typical human cell size.
  However, for low-resolution spatial data such as 10X visium, it should
  be the cell center-to-center distance (i.e.,
  `contact.range = 100` for 10X visium data). The function
  `computeCellDistance` can compute the center-to-center
  distance.

Instead of providing `contact.range`, users may
alternatively provide a value of `contact.knn.k`, in order to
restrict the contact-dependent signaling within the k-nearest neighbors
(knn). Please check the documentation of `computeCommunProb`
for more details via `help(computeCommunProb)`.

Below we provides suggestions on how to set the
`spatial.factors` for different types of spatial
transcriptomics data, including 10X Visium, Slide-seq, CosMx,
Stereo-seq, and seqFISH/merFISH/STARmap.

# 10X Visium

First, the spatial coordinates of spots from the full (NOT high/low)
resolution image should be used. For 10X Visium, this information is in
`tissue_positions.csv`. Given a Seurat object
`seu` of the spatial transcriptomics data, and spatial
coordinates can be obtained by

```
spatial.locs = GetTissueCoordinates(seu, scale = NULL, cols = c("imagerow", "imagecol"))
```

Second, the conversion factor of converting spatial coordinates from
Pixels to Micrometers can be computed as the ratio of the theoretical
spot size (i.e., 65um) over the number of pixels that span the diameter
of a theoretical spot size in the full-resolution image (i.e.,
‘spot\_diameter\_fullres’ in pixels in the ‘scalefactors\_json.json’ file).
Specifically, CellChat sets the `spatial.factors` as
follows:

```
scalefactors = jsonlite::fromJSON(txt = file.path("./tutorial/spatial_imaging_data_visium-brain", 'scalefactors_json.json'))

spot.size = 65 # the theoretical spot size (um) in 10X Visium
conversion.factor = spot.size/scalefactors$spot_diameter_fullres
spatial.factors = data.frame(ratio = conversion.factor, tol = spot.size/2)

d.spatial <- computeCellDistance(coordinates = spatial.locs, ratio = spatial.factors$ratio, tol = spatial.factors$tol)
min(d.spatial[d.spatial!=0]) # this value should approximately equal 100um for 10X Visium data
```

Finally, users can create a new CellChat object by taking spatial
coordinates and spatial.factors as inputs:

```
cellchat <- createCellChat(object = data.input, meta = meta, group.by = "labels",
                           datatype = "spatial", coordinates = spatial.locs, spatial.factors = spatial.factors)
```

Alternatively, users can create a new Spatial CellChat object when
runnng CellChat v3:

```
cellchat <- createSpatialCellChat(object = data.input, meta = meta, group.by = "labels",
                           datatype = "spatial", coordinates = spatial.locs, spatial.factors = spatial.factors)
```

# Xenium/visium HD/seqFISH/merFISH/STARmap

For single-cell/subcellular resolution spatial technologies like
Xenium/visium HD/seqFISH/merFISH/STARmap data, the spatial coordinates
are already in micrometers and thus the conversion factor is 1. The
spot.size can be the typical human cell size (i.e., 10um).

```
conversion.factor = 1
spot.size = 10 # use the typical human cell size
spatial.factors = data.frame(ratio = conversion.factor, tol = spot.size/2)
```

# Slide-seq

For Slide-seq data, the spatial coordinates are in pixels and the
conversion factor of converting spatial coordinates from Pixels to
Micrometers is 0.73. The beads used in the current protocol have a
diameter of 10um. Thus, we directly set the `spatial.factors`
as follows:

```
conversion.factor = 0.73; spot.size = 10
spatial.factors = data.frame(ratio = conversion.factor, tol = spot.size/2)
```

# CosMx

On the NanoString blogpost Comparing AtoMx™ SIP flat files with the
legacy format [https://nanostring-biostats.github.io/CosMx-Analysis-Scratch-Space/posts/flat-file-exports/flat-files-compare.html]
they state: “The pixel edge length is 120 nm. Thus, to convert to
microns multiply the pixel value by 0.12028 µm per pixel.”

Thus, for CosMx data, the spatial coordinates are in pixels and the
conversion factor of converting spatial coordinates from Pixels to
Micrometers is 0.12028. Because CosMx does not include the uniform cell
size like 10X Visium and Slide-seq, users can compute the minimum cell
centroid-to-centroid distance via `computeCellDistance` and
use its half value as the tolerance factor. Of note, CellChat does not
need an accurate tolerance factor, which is used for determining whether
considering the cell-pair as spatially proximal if their distance is
greater than `interaction.range` but smaller than
“`interaction.range` + `tol`”.

Thus, we set the `spatial.factors` as follows:

```
conversion.factor = 0.12028
d = computeCellDistance(spatial.locs)
spot.size = min(d)*conversion.factor # converting the distance in Pixels to Micrometers
spatial.factors = data.frame(ratio = conversion.factor, tol = spot.size/2)
```

# Stereo-seq

For Stereo-seq data, please check the the post https://github.com/jinworks/CellChat/issues/6

# Application to a dataset with a small panel of genes

If there are only a few number of L-R pairs from the gene panel of
the spatial dataset that can be found in the CellChat database, users
can modify the step on the identification of over-expressed genes
without performing DE. This can be done as follows

```
cellchat <- identifyOverExpressedGenes(cellchat, do.DE = FALSE, min.cells = 10)
```

# Application to multiple spatial transcriptomics datasets

Please check the key steps of applying
CellChat to multiple spatial transcriptomics datasets for detailed
descriptions.

For comparison analysis of multiple spatial transcriptomics datasets
across different biological conditions (e.g., health vs. diseased),
users still need to create a CellChat object seperately for each
condition. Please check the key steps of applying
CellChat to multiple non-spatial transcriptomics datasets for
detailed descriptions.
