# Supplementary material for: CSNK2B Expression Aligns with a GDF15-Associated Immune Molecular State in Hepatocellular Carcinoma
Source: Biomedicines. 2026 Aug 26;14(9):1909. doi: 10.3390/biomedicines14091909 (PMC13604900; doi:10.3390/biomedicines14091909)
Supplement: Supplementary file 1 [file biomedicines-14-01909-s001.zip › Supplemental file S1/docker_cellchat/CellChat-main-http1.tar.gz!/CellChat-main/tutorial/Interface_with_other_single-cell_analysis_toolkits.html]

Interface with other single-cell analysis toolkits


# Interface with other single-cell analysis toolkits

#### Suoqin Jin

#### 22 February, 2024

- Converting
  between single-cell objects (Seurat, SingleCellExperiment and anndata
  objects)
- TROUBLESHOOTING
  when starting from a AnnData object
- Part
  I: Prepare required input data matrix and meta data for creating a
  CellChat object
  - Required data inputs
  - (A) Starting from a count
    data matrix
  - (B) Starting from a Seurat
    object
  - (C) Starting from
    a SingleCellExperiment object
  - (D) Starting from a AnnData
    object
    - Using anndata R package
    - Using reticulate R package
    - Exporting required
      data directly from Python
- Part
  II: Create a CellChat object directly from Seurat, SingleCellExperiment
  or Scanpy object
  - (A)
    Starting from the digital gene expression matrix and cell label
    information
  - (B) Starting from a Seurat
    object
  - (C) Starting
    from a SingleCellExperiment object
  - (D) Starting from a AnnData
    object
- Part
  III: Interface with other cell-cell communication analysis toolkits
  - Use
    other ligand-receptor interaction databases for CellChat
    analysis
  - Use
    other computed cell-cell interaction scores for CellChat
    analysis

# Converting between single-cell objects (Seurat, SingleCellExperiment and anndata objects)

There are several excellent tools to convert between Seurat object, SingleCellExperiment
object, and anndata
object. Here are two examples: one is Seurat
R package from Satija Lab, another is zellkonverter R
package from Theis Lab, and sceasy.

Below we shows how to extract the CellChat input files as data matrix
from other existing single-cell analysis toolkits, including Seurat and Scanpy.

# TROUBLESHOOTING when starting from a AnnData object

If the RStudio encounters FATAL ERROR when creating a CellChat object
from a AnnData/Scanpy object, users can save the required data files
`data.input` and `meta` in user’s local computer
and then reload them for CellChat analysis.

If the RStudio encounters FATAL ERROR when reading .h5ad file using
zellkonverter::readH5AD, users can first insall the anndata R package
via `install.packages("anndata")`.

# Part I: Prepare required input data matrix and meta data for creating a CellChat object

## Required data inputs

CellChat requires two user inputs: one is the gene expression data of
cells, and the other is the user assigned cell labels.

- *For the gene expression data matrix*, genes should be in
  rows with rownames and cells in columns with colnames. Normalized data
  is required as input for CellChat analysis, e.g., library-size
  normalization and then log-transformed with a pseudocount of 1. If user
  provides count data, we provide a `normalizeData` function to
  account for library size.
- *For the cell group information*, a dataframe with
  rownames is required as input for CellChat.

In addition to taking a count data matrix as an input, we also
provide instructions for how to prepare CellChat input files from other
existing single-cell analysis toolkits, including Seurat and Scanpy.
Please start to prepare the input data by following option A when the
normalized count data and meta data are available, option B when the
Seurat object is available, option C when the SingleCellExperiment
object is available, and option D when the Scanpy object is
available.

## (A) Starting from a count data matrix

```
load("./tutorial/data_humanSkin_CellChat.rda")
data.input = data_humanSkin$data # normalized data matrix
meta = data_humanSkin$meta # a dataframe with rownames containing cell mata data
# Subset the input data for CelChat analysis
cell.use = rownames(meta)[meta$condition == "LS"] # extract the cell names from disease data
data.input = data.input[, cell.use]
meta = meta[cell.use, ]
```

## (B) Starting from a Seurat object

The normalized count data and cell group information can be obtained
from the Seurat object by

```
data.input <- seurat_object[["RNA"]]@data # normalized data matrix
# For Seurat version >= “5.0.0”, get the normalized data via `seurat_object[["RNA"]]$data`
labels <- Idents(seurat_object)
meta <- data.frame(group = labels, row.names = names(labels)) # create a dataframe of the cell labels
```

## (C) Starting from a SingleCellExperiment object

The normalized logcount data and cell group information can be
obtained from the SingleCellExperimen object by

```
data.input <- SingleCellExperiment::logcounts(object) # normalized data matrix
meta <- as.data.frame(SingleCellExperiment::colData(object)) # extract a dataframe of the cell labels
meta$labels <- meta[["sce.clusters"]]
```

Below is a reproducible example:

```
library(SingleCellExperiment)
library(basilisk)
library(scRNAseq)
sce <- SegerstolpePancreasData()
sce
counts <- assay(sce, "counts")
libsizes <- colSums(counts)
size.factors <- libsizes/mean(libsizes)
logcounts(sce) <- log2(t(t(counts)/size.factors) + 1)

data.input <- SingleCellExperiment::logcounts(sce) # normalized data matrix
meta <- as.data.frame(SingleCellExperiment::colData(sce)) # extract a dataframe of the cell labels
meta$labels <- meta[["cell.type"]]
```

## (D) Starting from a AnnData object

anndata provides a python class that can be used to store single-cell
data, which has been widely used in various Python tools such as the
scanpy python package.

### Using anndata R package

anndata for R,
which is a reticulate wrapper for the anndata Python package, was
recently published in CRAN, which makes it much convenient to read from
and write to the h5ad file format in R. We show how to extract the
required data inputs for CellChat analysis using anndata R package.

```
# install.packages("anndata")
# read the data into R using anndata R package
library(anndata)
ad <- read_h5ad("scanpy_object.h5ad")
# access count data matrix
counts <- t(as.matrix(ad$X))
# normalize the count data if the normalized data is not available in the .h5ad file
library.size <- Matrix::colSums(counts)
data.input <- as(log1p(Matrix::t(Matrix::t(counts)/library.size) * 10000), "dgCMatrix")

# access meta data
meta <- ad$obs
meta$labels <- meta[["ad_clusters"]] 
# save the data for CellChat analysis
save(data.input, meta, file = "test_data.RData")
```

### Using reticulate R package

User can also read the data into R using the reticulate package to
import the anndata module.

```
# read the data into R using the reticulate package to import the anndata module
library(reticulate)
ad <- import("anndata", convert = FALSE)
ad_object <- ad$read_h5ad("scanpy_object.h5ad")
# access normalized data matrix
data.input <- t(py_to_r(ad_object$X))
rownames(data.input) <- rownames(py_to_r(ad_object$var))
colnames(data.input) <- rownames(py_to_r(ad_object$obs))
# access meta data
meta.data <- py_to_r(ad_object$obs)
meta <- meta.data
```

Please also check the suggestions by other USERS (https://github.com/sqjin/CellChat/issues/300#issuecomment-1116343516)

### Exporting required data directly from Python

User can also export expression matrix and meta data from h5ad in
Python.

```
pd.DataFrame(ad.var.index).to_csv(os.path.join(destination, "genes.tsv" ),   sep = "\t", index_col = False)
pd.DataFrame(ad.obs.index).to_csv(os.path.join(destination, "barcodes.tsv"), sep = "\t", index_col = False)
ad.obs.to_csv(os.path.join(destination, "metadata.tsv"), sep = "\t", index_col = True)
adata.T.to_df().to_csv('matrix.csv')
```

Please see more discussion on how to export expression matrix and
meta data from h5ad (https://github.com/scverse/scanpy/issues/262)

# Part II: Create a CellChat object directly from Seurat, SingleCellExperiment or Scanpy object

From CellChat version 0.5.0, **USERS can create a new CellChat
object from Seurat or SingleCellExperiment object.** . If input
is a Seurat or SingleCellExperiment object, the meta data in the object
will be used by default and USER must provide `group.by` to
define the cell groups. e.g, group.by = “ident” for the default cell
identities in Seurat object.

Please check the examples in the documentation of
`createCellChat` for details via
`help(createCellChat)`.

**NB: If USERS load previously calculated CellChat object
(version < 0.5.0), please update the object via
`updateCellChat`**

Create a CellChat object by following option A when taking the
digital gene expression matrix and cell label information as input,
option B when taking a Seurat object as input, option C when taking a
SingleCellExperiment object as input, and option D when taking a AnnData
object as input. See details in the subsection of Required input data.
User can initialize a CellChat object using the createCellChat function
as follows:

## (A) Starting from the digital gene expression matrix and cell label information

Upon extracting the required CellChat input files, then create a
CellChat object and start the analysis.

```
library(CellChat)
cellchat <- createCellChat(object = data.input, meta = meta, group.by = "labels")
```

If cell mata information is not added when creating CellChat object,
Users can also add it later using `addMeta`, and set the
default cell identities using `setIdent`.

```
cellchat <- addMeta(cellchat, meta = meta, meta.name = "labels")
cellchat <- setIdent(cellchat, ident.use = "labels") # set "labels" as default cell identity
levels(cellchat@idents) # show factor levels of the cell labels
```

## (B) Starting from a Seurat object

```
library(CellChat)
cellChat <- createCellChat(object = seurat.obj, group.by = "ident", assay = "RNA")
```

## (C) Starting from a SingleCellExperiment object

```
library(CellChat)
cellChat <- createCellChat(object = sce.obj, group.by = "sce.clusters")
```

## (D) Starting from a AnnData object

```
sce <- zellkonverter::readH5AD(file = "adata.h5ad")
sce
# retrieve all the available assays within sce object
assayNames(sce)
# added a new assay entry "logcounts" if not available
counts <- assay(sce, "X") # make sure this is the original count data matrix
library.size <- Matrix::colSums(counts)
logcounts(sce) <- log1p(Matrix::t(Matrix::t(counts)/library.size) * 10000)

# extract a cell meta data
meta <- as.data.frame(SingleCellExperiment::colData(sce)) #
# save the sce object for CellChat analysis
save(sce, file = "sce_object.RData")

# create a CellChat object from the sce object
cellChat <- createCellChat(object = sce, group.by = "clusters.final")
```

# Part III: Interface with other cell-cell communication analysis toolkits

## Use other ligand-receptor interaction databases for CellChat analysis

To use other ligand-receptor interaction databases for CellChat
analysis, users need to convert the source database to the format of
CellChatDB using the function named `updateCellChatDB`.
Please check the tutorial named “Update-CellChatDB.html” (https://htmlpreview.github.io/?https://github.com/jinworks/CellChat/blob/master/tutorial/Update-CellChatDB.html)
for more details on updating CellChatDB by integrating new
ligand-receptor pairs from other resources or utilizing a custom
ligand-receptor interaction database.

## Use other computed cell-cell interaction scores for CellChat analysis

To use CellChat for downstream analysis of cell-cell communication
from other cell-cell communication tools, users can use the function
named `updateCCC_score`, which allows users to provide
customized cell-cell interaction scores. Please check the detailed
documentation of this function via `help(updateCCC_score)`
.
