# Supplementary material for: CSNK2B Expression Aligns with a GDF15-Associated Immune Molecular State in Hepatocellular Carcinoma
Source: Biomedicines. 2026 Aug 26;14(9):1909. doi: 10.3390/biomedicines14091909 (PMC13604900; doi:10.3390/biomedicines14091909)
Supplement: Supplementary file 1 [file biomedicines-14-01909-s001.zip › Supplemental file S1/docker_cellchat/CellChat-main-http1.tar.gz!/CellChat-main/tutorial/Update-CellChatDB.html]

Update CellChatDB by integrating new ligand-receptor pairs from other resources or utilizing a custom ligand-receptor interaction database


# Update CellChatDB by integrating new ligand-receptor pairs from other resources or utilizing a custom ligand-receptor interaction database

#### Suoqin Jin

#### 22 February, 2024

- Part
  I: update CellChatDB using the function
  `updateCellChatDB`
  - Example
    1: using L-R interactions from a simple dataframe
    - Load the required
      libraries
    - Step
      1: Load the customized ligand-receptor pairs and the gene
      information
    - Step
      2: Formulate the input files to be compatible with CellChatDB
    - Step
      3: Update CellChatDB by running the function
      `updateCellChatDB`
    - Step
      4: Use the new database in CellChat analysis or re-build CellChat
      package
  - Example 2:
    using L-R interactions from CellPhoneDB
    - Load the required
      libraries
    - Step 1: Load the database
      files
    - Step
      2: Formulate the input files to be compatible with CellChatDB
    - Step
      3: Update CellChatDB by running the function
      `updateCellChatDB`
    - Step
      4: Use the new database in CellChat analysis or re-build CellChat
      package
- Part
  II: update CellChatDB by manually modifying the required files
  - Load the required
    libraries
  - Step
    1: Access the ligand-receptor interaction information in
    CellChatDB
  - Step
    2: Update the required files by adding users’ curated ligand-receptor
    pairs
  - Step 3: Update CellChatDB
  - Step 4: Re-build
    CellChat package (optional)

# Part I: update CellChatDB using the function `updateCellChatDB`

CellChat v2 provides the function `updateCellChatDB` to
allow users to update CellChatDB by integrating new ligand-receptor
pairs from other resources or utilizing a custom ligand-receptor
interaction database. This function can be used as follows:

```
updateCellChatDB(
  db,
  gene_info = NULL,
  other_info = NULL,
  merged = FALSE,
  species_target = NULL
)
```

The minimum input of this function is a data frame with two columns
named as `ligand` and `receptor`, and other inputs
include classified pathway, complex, cofactor and gene information.
Users can check the documentation of this function for more details of
the required input data by typing `help(updateCellChatDB)` in
RStudio.

## Example 1: using L-R interactions from a simple dataframe

Here we describe how to integrate other resources by taking another
database CellTalkDB as an example. After downloading the CellTalkDB from
https://github.com/ZJUFanLab/CellTalkDB, follow the four
steps as follows.

### Load the required libraries

```
library(CellChat)
options(stringsAsFactors = FALSE)
```

### Step 1: Load the customized ligand-receptor pairs and the gene information

The first input `db` is a data frame with as least two
columns named as `ligand` and `receptor`. We
highly suggest users to provide a column of pathway information named
‘pathway\_name’ associated with each L-R pair. Other optional columns
include ‘interaction\_name’ and ‘interaction\_name\_2’. The default columns
of CellChatDB can be checked via
‘colnames(CellChatDB.human$interaction)’.

**Warning: If no pathway information of each L-R pair is
provided, all pathway-level analysis from CellChat cannot be used, such
as `computeCommunProb`!**

The second input `gene_info` is a data frame with at least
one column named as ‘Symbol’. “When setting gene\_info = NULL, the input
‘species\_target’ should be provided: either ‘human’ or ‘mouse’.

The third input `other_info` is a list consisting of other
information including a dataframe named as ‘complex’ and a dataframe
named as ‘cofactor’. This additional information is not necessary. If
other\_info is provided, the ‘complex’ and ‘cofactor’ are dataframes with
defined rownames.

```
# Load the dataframe consisting of customized ligand-receptor pairs
db.user <- readRDS("./CellTalkDB-master/database/human_lr_pair.rds")
# Load the dataframe consisting of gene information (optional)
gene_info <- readRDS("./CellTalkDB-master/data/human_gene_info.rds")
```

### Step 2: Formulate the input files to be compatible with CellChatDB

Please check above for the basic requirement of the input files. In
addition, users can check the detailed database information in
CellChatDB.

- The main ligand-receptor interaction information is stored in
  `CellChatDB$interaction`. Columns of this dataframe includes
  information of ligands, receptors and co-factors.
- `CellChatDB$geneInfo` contains all gene information in
  mouse or other species and it should have a column named ‘Symbol’, which
  is not necessary to be changed when updating CellChatDB.
- Optional information is stored in `CellChatDB$complex`
  and `CellChatDB$cofactor`, which include any ligand complex,
  receptor complex and cofactors. Users need to make sure that
  user-defined complex/cofactor names are the same in
  `CellChatDB$interaction`.

```
# Modify the colnames because of incompatible with `colnames(CellChatDB.human$interaction)`
colnames(db.user) <- plyr::mapvalues(colnames(db.user), from = c("ligand_gene_symbol","receptor_gene_symbol","lr_pair"), to = c("ligand","receptor","interaction_name"), warn_missing = TRUE)
```

### Step 3: Update CellChatDB by running the function `updateCellChatDB`

```
# Create a new database by typing one of the following commands
# Use user-provided gene information
db.new <- updateCellChatDB(db = db.user, gene_info = gene_info)
# Use built-in gene information of CellChatDB
db.new <- updateCellChatDB(db = db.user, gene_info = NULL, species_target = "human")

# Alternatively, users can integrate the customized L-R pairs into the built-in CellChatDB 
db.new <- updateCellChatDB(db = db.user, merged = TRUE, species_target = "human")
```

**Warning: Becuase no pathway information of each L-R pair is
provided in `db.user` here, all pathway-level analysis from
CellChat cannot be used, such as `computeCommunProb`!**

### Step 4: Use the new database in CellChat analysis or re-build CellChat package

Re-build CellChat package by updating the database as follows

```
# Users can now use this new database in CellChat analysis 
cellchat@DB <- db.new

# Users can save the new database for future use
save(db.new, file = "CellChatDB.human_user.rda")

# Users can also re-build CellChatDB in CellChat package
setwd("/Users/$USERS/Downloads/CellChat-master") # This is the folder of CellChat package downloaded from Github
CellChatDB.mouse <- CellChatDB
usethis::use_data(CellChatDB.mouse, overwrite = TRUE)

# If working on a human dataset, do following:
# CellChatDB.human <- CellChatDB
# usethis::use_data(CellChatDB.human, overwrite = TRUE)
```

## Example 2: using L-R interactions from CellPhoneDB

Since cellphonedb v5, cellphonedb also introduces signalling
directionality (ligand is partner A, receptor partner B) and
classification of signaling pathways, which makes it easiler to make
full use of CellChat’s versatile analysis by taking CellPhoneDB as an
input. Here we describe how to integrate L-R interaction resources from
CellPhoneDB. After downloading the cellphonedb v5 from https://github.com/ventolab/cellphonedb-data, run the
following codes.

### Load the required libraries

```
library(CellChat)
options(stringsAsFactors = FALSE)
```

### Step 1: Load the database files

```
## load the database files
interaction_input <- read.csv(file = './otherDB/cellphonedb-data-master/data/interaction_input.csv')
complex_input <- read.csv(file = './otherDB/cellphonedb-data-master/data/complex_input.csv', row.names = 1)
geneInfo <- read.csv(file = './otherDB/cellphonedb-data-master/data/gene_input.csv')
geneInfo$Symbol <- geneInfo$hgnc_symbol
geneInfo <- select(geneInfo, -c("ensembl"))
geneInfo <- unique(geneInfo)
```

### Step 2: Formulate the input files to be compatible with CellChatDB

```
## prepare interaction_input file
# get the ligand information
idx_partnerA <- match(interaction_input$partner_a, geneInfo$uniprot)
idx.use <- !is.na(idx_partnerA)
interaction_input$ligand <- interaction_input$partner_a
interaction_input$ligand[idx.use] <- geneInfo$hgnc_symbol[idx_partnerA[idx.use]]
# get the receptor information
idx_partnerB <- match(interaction_input$partner_b, geneInfo$uniprot)
idx.use <- !is.na(idx_partnerB)
interaction_input$receptor <- interaction_input$partner_b
interaction_input$receptor[idx.use] <- geneInfo$hgnc_symbol[idx_partnerB[idx.use]]
# get other information
interaction_input$interaction_name <- interaction_input$interactors
interaction_input$interaction_name_2 <- interaction_input$interaction_name
interaction_input$pathway_name <- interaction_input$classification
interaction_input$pathway_name <- gsub(".*by ", "", interaction_input$pathway_name)
interaction_input$annotation <- interaction_input$directionality
interaction_input <- select(interaction_input, -c("partner_a","partner_b","protein_name_a","protein_name_b","interactors","classification","directionality"))

## prepare complex_input file
complexsubunits <- dplyr::select(complex_input, starts_with("uniprot"))
for (i in 1:ncol(complexsubunits)) {
  idx_complex <- match(complex_input[,paste0("uniprot_",i)], geneInfo$uniprot)
  idx.use <- !is.na(idx_complex)
  complex_input[idx.use,paste0("uniprot_",i)] <- geneInfo$hgnc_symbol[idx_complex[idx.use]]
}
colnames(complex_input)[1:ncol(complexsubunits)] <- paste0("subunit_",seq_len(ncol(complexsubunits)))
complex_input <- dplyr::select(complex_input, starts_with("subunit"))

## prepare other information 
other_info <- list(complex = complex_input)
```

### Step 3: Update CellChatDB by running the function `updateCellChatDB`

```
db.new <- updateCellChatDB(db = interaction_input, gene_info = geneInfo, other_info = other_info, trim.pathway = T)
```

### Step 4: Use the new database in CellChat analysis or re-build CellChat package

Re-build CellChat package by updating the database as follows

```
# Users can now use this new database in CellChat analysis 
cellchat@DB <- db.new

# Users can save the new database for future use
save(db.new, file = "CellChatDB.human_user.rda")

# Users can also re-build CellChatDB in CellChat package
setwd("/Users/$USERS/Downloads/CellChat-master") # This is the folder of CellChat package downloaded from Github
CellChatDB.human <- CellChatDB
usethis::use_data(CellChatDB.human, overwrite = TRUE)
```

# Part II: update CellChatDB by manually modifying the required files

Here we outline the steps to update CellChatDB by manually adding
user-defined ligand-receptor pairs. To do so, the format of the users’
lists must be compatible with the input files of CellChatDB.

## Load the required libraries

```
library(CellChat)
options(stringsAsFactors = FALSE)
```

## Step 1: Access the ligand-receptor interaction information in CellChatDB

Extract the database information in CellChatDB and then save them in
a local computer, including four files: ‘geneInfo.csv’,
‘interaction\_input\_CellChat.csv’, ‘complex\_input\_CellChat.csv’, ‘and
cofactor\_input\_CellChat.csv’. Users can do it by running the following
codes in Rstudio:

```
CellChatDB <- CellChatDB.mouse # set CellChatDB <- CellChatDB.human if working on the human dataset
interaction_input <- CellChatDB$interaction
complex_input <- CellChatDB$complex
cofactor_input <- CellChatDB$cofactor
geneInfo <- CellChatDB$geneInfo
write.csv(interaction_input, file = "interaction_input_CellChatDB.csv")
write.csv(complex_input, file = "complex_input_CellChatDB.csv")
write.csv(cofactor_input, file = "cofactor_input_CellChatDB.csv")
write.csv(geneInfo, file = "geneInfo_input_CellChatDB.csv")
```

## Step 2: Update the required files by adding users’ curated ligand-receptor pairs

Update the above four .csv files by adding users’ curated
ligand-receptor pairs.

- The main file is ‘interaction\_input\_CellChatDB.csv’. Users can
  first update the ligands, receptors and co-factors in the corresponding
  columns in ‘interaction\_input\_CellChatDB.csv’.
- Users can then update “complex\_input\_CellChatDB.csv” and
  “cofactor\_input\_CellChatDB.csv” if any ligand complex, receptor complex
  and cofactors are updated. Users need to make sure that user-defined
  complex/cofactor names are the same in
  ‘interaction\_input\_CellChatDB.csv’ and “complex\_input\_CellChatDB.csv”,
  ‘interaction\_input\_CellChatDB.csv’ and ”
  cofactor\_input\_CellChatDB.csv”.
- “geneInfo\_input\_CellChatDB.csv” contains all gene information in
  mouse and it should have a column named ‘Symbol’, which does not need to
  be changed when updating CellChatDB.

## Step 3: Update CellChatDB

Update CellChatDB once updating the four .csv files. Users can do it
by running the following codes in Rstudio:

```
options(stringsAsFactors = FALSE)
interaction_input <- read.csv(file = 'interaction_input_CellChatDB.csv', row.names = 1)
complex_input <- read.csv(file = 'complex_input_CellChatDB.csv', row.names = 1)
cofactor_input <- read.csv(file = 'cofactor_input_CellChatDB.csv', row.names = 1)
geneInfo <- read.csv(file = ' geneInfo_input_CellChatDB.csv', row.names = 1)
CellChatDB <- list()
CellChatDB$interaction <- interaction_input
CellChatDB$complex <- complex_input
CellChatDB$cofactor <- cofactor_input
CellChatDB$geneInfo <- geneInfo
```

## Step 4: Re-build CellChat package (optional)

Re-build CellChat package by updating the database as follows

```
setwd("/Users/$USERS/Downloads/CellChat-master") # This is the folder of CellChat package downloaded from Github
CellChatDB.mouse <- CellChatDB
usethis::use_data(CellChatDB.mouse, overwrite = TRUE)

# If working on a human dataset, do following:
# CellChatDB.human <- CellChatDB
# usethis::use_data(CellChatDB.human, overwrite = TRUE)
```
